# Unsupervised manifold embedding to encode molecular quantum information for supervised learning of chemical data

**DOI:** 10.1038/s42004-024-01217-z

**Published:** 2024-06-11

**Authors:** Tonglei Li, Nicholas J. Huls, Shan Lu, Peng Hou

**Affiliations:** Deparment of Industrial and Molecular Pharmaceutics, Purdue University, West Lafayette, 47907 IN USA

**Keywords:** Theoretical chemistry, Cheminformatics

## Abstract

Molecular representation is critical in chemical machine learning. It governs the complexity of model development and the fulfillment of training data to avoid either over- or under-fitting. As electronic structures and associated attributes are the root cause for molecular interactions and their manifested properties, we have sought to examine the local electron information on a molecular manifold to understand and predict molecular interactions. Our efforts led to the development of a lower-dimensional representation of a molecular manifold, Manifold Embedding of Molecular Surface (MEMS), to embody surface electronic quantities. By treating a molecular surface as a manifold and computing its embeddings, the embedded electronic attributes retain the chemical intuition of molecular interactions. MEMS can be further featurized as input for chemical learning. Our solubility prediction with MEMS demonstrated the feasibility of both shallow and deep learning by neural networks, suggesting that MEMS is expressive and robust against dimensionality reduction.

## Abstract (web summary)

In machine learning of molecular properties, adequate molecular representation is crucial, as minor structural changes result in significant differences in the activity of interest. Here, the authors use a lower-dimensional representation of a molecular manifold to embed electronic attributes and retain the chemical intuition of molecular interactions, invariant to translational and rational degrees of freedom of the molecular surface, to ease model complexities and facilitate generalization even with relatively small datasets.

## Introduction

In chemical learning, a molecule is encoded in a computable format to develop quantitative structure-activity or structure-property relationships (QSAR or QSPR) by a machine learning model. A molecule is often represented as an assembly or set of numerical descriptors, such as molecular weight, dipole moment, and number of single bonds. Moreover, the conventional depiction of a molecule as a graph of nodes and lines signifying atoms and bonds has initiated various description or fingerprinting schemes, such as SMILES^1^, and ECFP^2^. A descriptor is generally of a 1-, 2-, or 3-D feature of a molecule; the elemental composition and chemical connectivity may also be encoded as a fingerprint or alphanumeric string. While benchmarking studies have been conducted to show one representation outperforms another^3,4^, in principle, as long as it could fully differentiate molecules (in a molecular dataset), a set of descriptors, a graph representation, or a fingerprint would assume a one-to-one connection or function with the molecular property of interest, which could be approximated by machine learning. Nonetheless, there remain two interweaved challenges when applying a molecular description in data-driven chemical learning. The first one stems from the so-called Curse of Dimensionality (COD)^5^.

A set of descriptors or a fingerprint bears the dimensionality of its features. As the dimensionality increases, the covering of the chemical space by the same amount of data becomes exponentially reduced. In addition, the distance between any two points in a high-dimensional space is approximately identical, making any distance-based classification or regression prediction ineffective. Effectively reducing the dimensionality of molecular descriptors or chemical features is thus necessitated when developing data-driven prediction models. Multiple steps of dimensionality reduction, nonetheless, cause information degradation and demote the eventual discerning power and resolution of molecules, making machine learning difficult to infer the underlying or true function. The quandary might be well reflective of the observation by Hughes in 1968 that the predicting power of a classification model first increases and then declines as the number of descriptors increases^6^.

The empirical nature of utilizing conventional descriptors presents another challenge in chemical learning. Correlations are common among descriptors, requiring careful examination and removal of those that add little chemical intuition^7,8^. Most of molecular fingerprints or graph representations are extremely sparse, deepening the complexity of developing machine learning models. When molecular features carry no explicit information about molecular interactions, multiple latent functions with differing dimensionalities are expected to bridge the input structure and output property, requiring sophisticated learning architectures and numerous hidden layers of neural networks. However, using a “deep” learning framework could be futile because of the information loss incurred by dimensionality reduction steps to curb the COD. It is thus desired to represent a molecule by quantum mechanically derived quantities that orthogonally preserve the molecule’s chemical information and directly connect with molecular properties.

As molecular interactions are fundamentally described by quantum mechanics, featurization of electronic structures and attributes may overcome the above-mentioned challenges when learning to predict molecular properties. There have been many efforts to capture electronic quantities for machine learning^9^. One general approach is to augment a molecular graph with electronic attributes. The adjacency matrix of a molecule may be weighted by electronic or chemical properties localized to atoms or atomic pairs. One such development is the Coulomb matrix^10^; electron density-weighted connectivity matrix (EDWCM) is another concept^11^, in which the electron density at the bond critical point (BCP) is recorded for each bonded pair^12,13^. With a similar footing of partitioning the electron density, an electron localization-delocalization matrix (LDM) is devised with localized electron values assigned to the diagonal elements (atoms) and de-localized values assigned to the off-diagonal pairs^11^. There are also efforts to integrate electronic quantities that are derived from the second-order perturbation analysis (SOPA) in the context of natural bond orbital (NBO) theory in molecular graphs for machine learning^14,15^. In the recent development of OrbNet-Equi, the molecular representation may be regarded as an adjacency matrix where each element is a concatenated vector of respective parameters of single-electron operators, such as Fock and density matrices, on atomic orbitals^16^. Because of the underpinning of molecular topology, graph neural networks (GNNs)^17^, including convolutional GNNs (CGNNs) and message passing NNs (MPNNs), are often utilized to handle these representations. Alternatively, there are approaches to discretize the space of a molecule by a finite grid to retain the electron density and pertinent attributes for machine learning. Two noteworthy efforts are PIXEL^18^, where the valence-only electron densities are partitioned to voxels, and CoMFA^19^, where interaction energies against a probe atom traversing at the pre-determined grid points are recorded. These representations are however not invariant to rotation or orientation of a molecule, potentially limiting their usage.

Given the premise of representing molecules for chemical learning, we report a new concept of lower-dimensional embeddings of electron densities and local electronic attributes on a molecular surface. The concept of Manifold Embedding of Molecular Surface (MEMS) is aimed to preserve the quantum chemical information of molecular interactions by translation- and rotation-invariant feature vectors residing on a manifold. The conceptualization of MEMS is rooted in our studies of intermolecular interactions^20–26^. We exploited the hard and soft acids and bases principle (HSAB)^27,28^ within the framework of conceptual density functional theory (CDFT)^29–31^ to characterize intermolecular interactions in organic crystals^20–22,24,32,33^. Our studies unveiled that Fukui functions, electrostatic potential (ESP), and other density functional-derived quantities at the interface between two molecules quantitatively determine the locality and strength of intermolecular interactions^25,26^. A crucial finding was that the electronic properties of the single molecule – other than those of the explicitly interacted molecule pair – bear the information of both the strength and locality of intermolecular interactions^25,26^. We have been motivated to explore the intrinsic electronic attributes of a single molecule to study intermolecular interactions, more recently by neural networks.

Treating a molecular surface as a manifold, our concept of MEMS aligns with manifold learning – a manifold assumes a lower-dimensional embedding, which may be computationally derived by dimensionality reduction procedures^34–36^. A molecular surface is not a physical quantity but a chemical perception to partition the electron density of a molecule. It marks the boundary where intermolecular interactions – attraction and repulsion – mostly converge. There have been several efforts reported in the literature that utilize electronic attributes or chemical interaction quantities on a molecular surface to predict molecular properties. One earlier study was the development of self-organizing maps (SOMs) of molecules, where surface points are mapped to a regularly spaced 2-D grid based on neighborhood probabilities^37^. Spatial autocorrelation of electronic properties on a molecular surface was attempted, leading to a number of autocorrelation coefficients to be utilized in QSAR studies^38^. In the COSMO-RS approach, which is widely utilized in predicting a small molecule’s solubility in another solvent, the screening charge densities on a molecular surface are partitioned as a probability distribution profile (so-called σ profile) and employed in the prediction^39^. More recently, electronic attributes and several other chemical and geometric properties on a protein surface were directly featurized by a geodesic convolution approach and used in deep learning of protein interactions^40^. In the study, a patch of neighboring points on the triangulated mesh is aggregated around a surface vertex by applying a Gaussian kernel with trainable parameters defined by local geodesic and polar coordinates. For each vertex, multiple Gaussian kernels may be applied for convoluting surface chemical attributes, leading to a multi-dimensional, trainable fingerprint. A similar effort circumvented the mesh triangulation step and directly conducted geometric convolution on the point cloud of a protein surface^41^. In these geometric deep learning efforts, rotational invariance of the fingerprint is nonetheless numerically handled by attempting multiple instances of the surface orientation in training. Compared with the geodesic convolution efforts, our MEMS is unsupervised learning by manifold embedding of quantum mechanical quantities, requiring no training steps by data.

To generate manifold embeddings, we implemented a non-linear method of stochastic neighbor embedding (SNE)^42^, NeRV (neighbor retrieval visualizer)^43^. The process preserves the local neighborhood of surface points between the manifold and embedding. The neighborhood is defined by pairwise geodesic distances among surface points of the manifold (e.g., Hirshfeld surface^44^ or solvent-exclusion surface^45^). The local electronic attributes on a molecular surface are then mapped to the manifold embedding and further featurized as numerical matrices to encode the quantum information. We then demonstrated utilizing MEMS matrices to predict water solubilities in supervised learning with neural networks.

## Results

### Manifold embedding of molecular surface

**Fig. 1:**
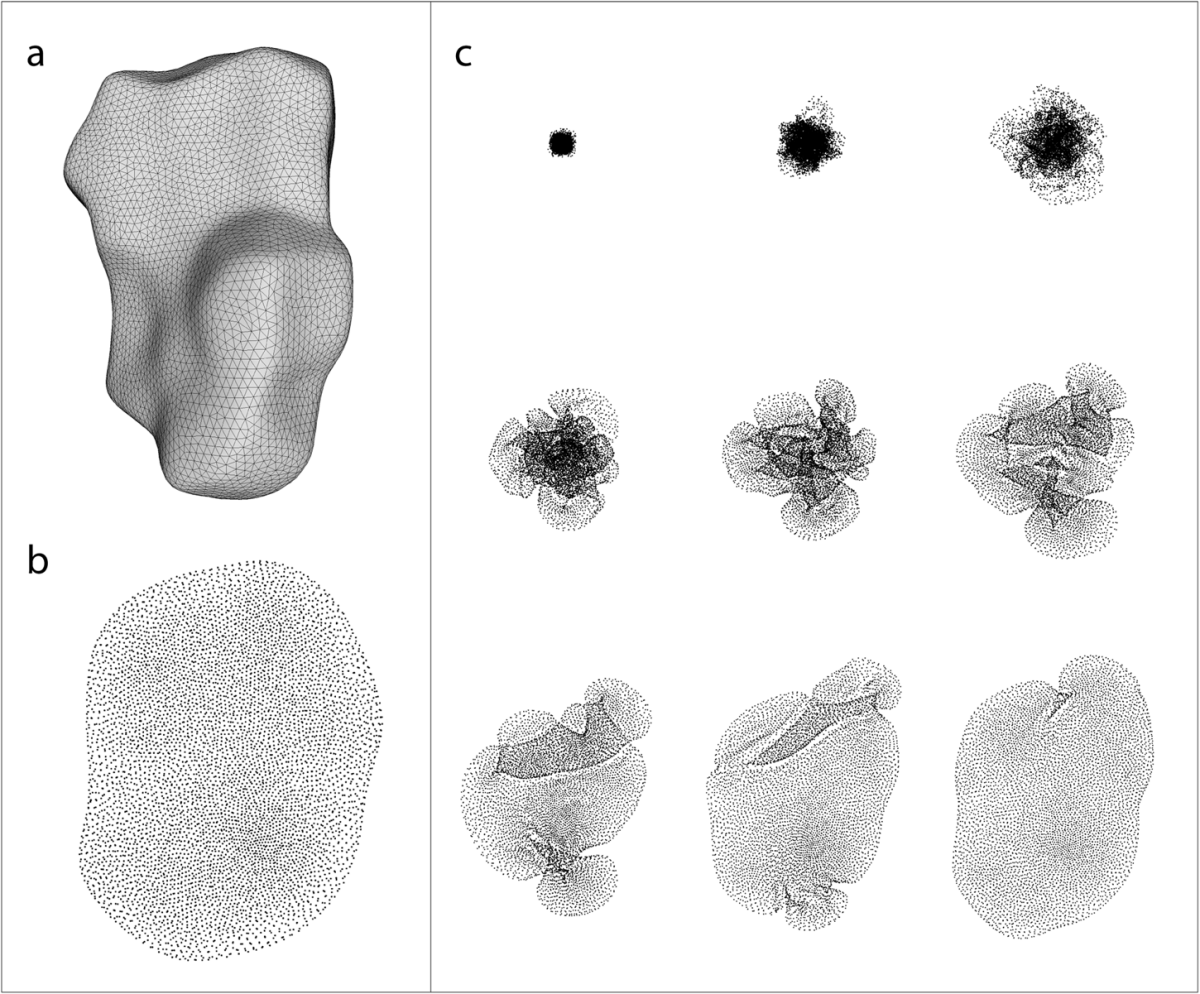
Illustration of dimensionality reduction of molecular surface. Hirshfeld surface of tolfenamic acid (**a**) to its manifold embedding (**b**). Several immediate steps of the optimization process are illustrated in (**c**).

The dimensionality reduction process of a Hirshfeld surface of tolfenamic acid (metastable or Form II)^46^ is illustrated in Fig. 1. The optimization process is demonstrated in Fig. 1c, where the initially randomized points were progressively re-positioned, reaching a local minimum of the cost function by the resultant MEMS.

Because a molecular surface is mathematically enclosed, some surface points fall in a wrong neighborhood on the embedding as false positives (distant neighbors in 3-D put in the same neighborhood on MEMS) or false negatives (near neighbors in 3-D separated on MEMS)^43^. We developed a basic scheme to estimate the percent of false points. By defining the neighborhood radius twice of the shortest inter-vertex distance on the 3-D surface, we assign a point as “outsider” if none of its MEMS neighbors originates from its 3-D neighborhood. From various cases we analyzed, the percentage of outsiders was 20-40%, depending on the geometry of a molecular surface. Thus, MEMS generated by NeRV seems to retain most of the chemical information on a molecular surface.

**Fig. 2:**
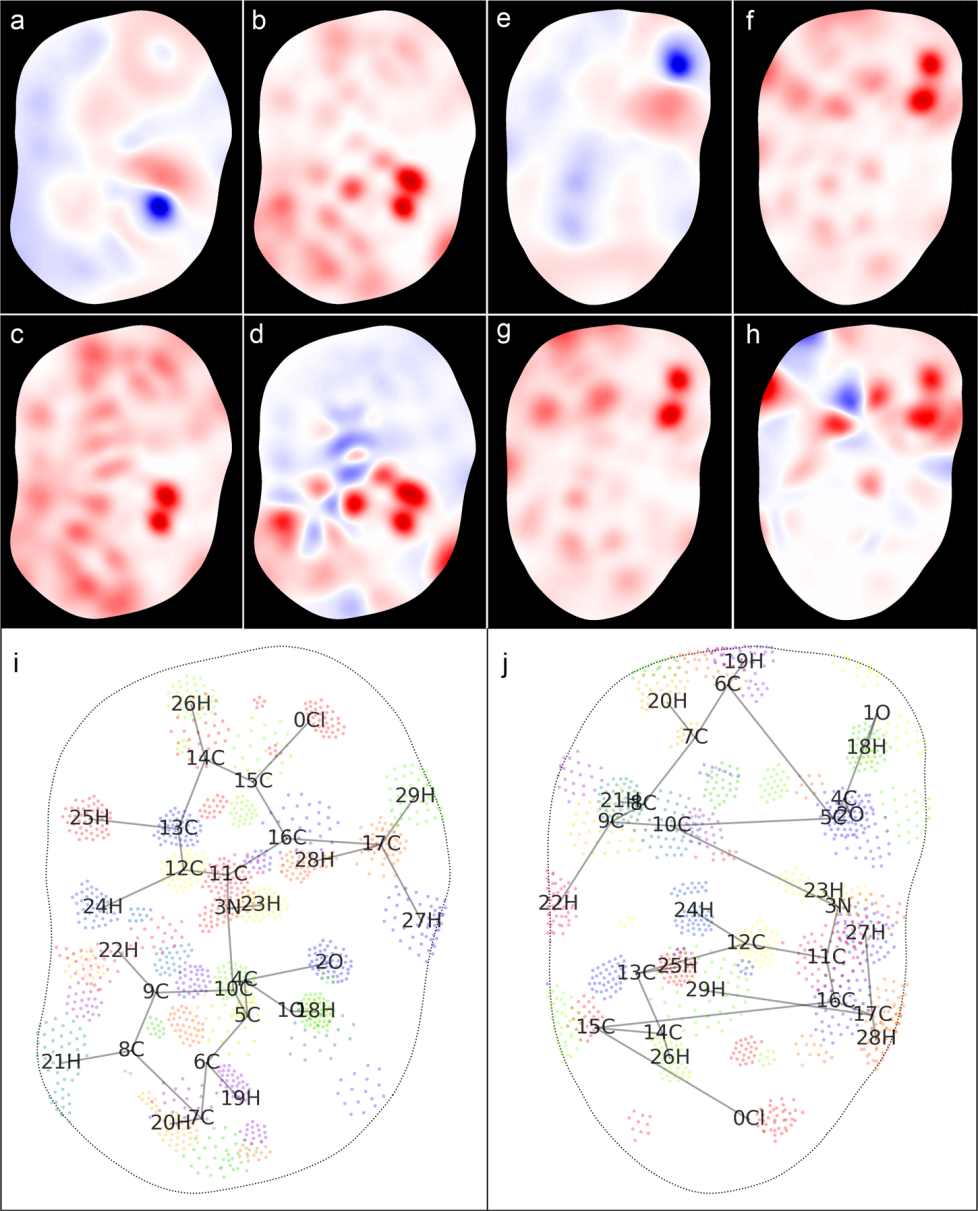
Hirshfeld surface MEMS of tolfenamic acid. The electronic properties on MEMS are ESP (**a** and **e**), F^+^ (**b** and **f**), F^-^ (**c** and **g**), and F^2^ (**d** and **h**). The color scheme varies from blue to white and to red as the value goes from negative to neutral and to positive; the trend is opposite for ESP. Embedding points of the 50 nearest surface vertices to each atom are shown in (**i** and **j**) of the two conformers with atom labels and bondings marked. The left panel (**a**-**d** and **i**) is of Form II and the right panel of Form I.

Figure 2 showcases a few interpolated MEMS that are color-coded with electronic properties on the corresponding manifolds. The electronic properties (electrostatic potential or ESP, nucleophilic Fukui function or F^+^, electrophilic Fukui function or F^-^, and dual descriptor of Fukui function or F^2^) were calculated of the single molecules, whose conformations were extracted from the crystal structures. The two MEMS in Fig. 2 are of the same molecule but different conformations, revealing that major electronic properties and spatial patterns are preserved. The subtle and yet significant differences between the two crystal forms are captured by the 2-D embeddings. Note that the color scale in Fig. 2 is relative to the respective electronic attributes. Each image has its largest value scaled to a full byte with positive numbers assigned to the red channel and negative to the blue of the image (opposite for ESP). The most outstanding region of the electronic properties is of the carboxyl group; its adjacent aromatic ring seems more polarized than the other ring.

**Fig. 3:**
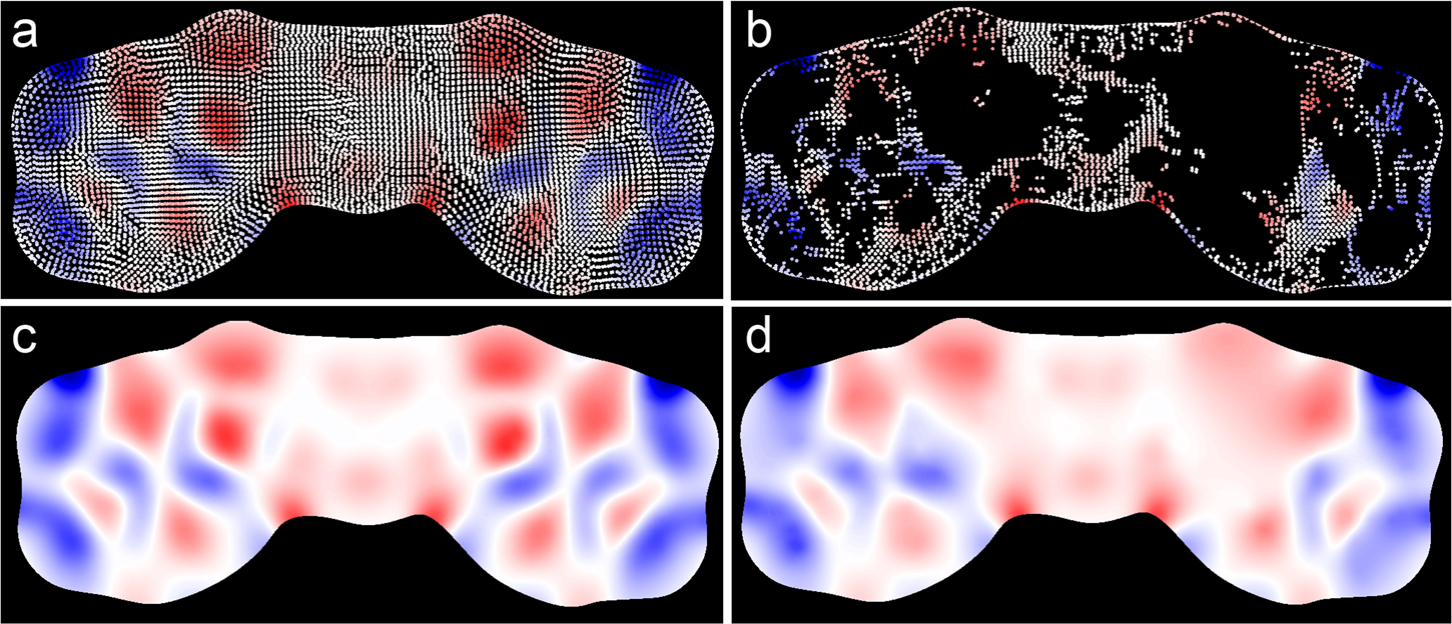
RBF interpolation of MEMS. Interpolated embedding image (**c**) of all embedding points (**a**) and interpolated MEMS (**d**) after the removal of about 64% of embedding points (**b**). The molecule is N,N’-trimethylenebis(3,4-dihydroxybenzamide).

Interpolation of electronic values on the MEMS in Fig. 2 was conducted by Gaussian-based radial basis functions (RBFs). Because of Gaussian kernels, the interpolation preserves dominant electronic attributes but smooths out minor features on the embeddings. In our case, most false positives and negatives were averaged out by the interpolation process. Figure 3 demonstrates one extreme case where 3247 points were retained out of 9011 total embedding points, while the RBF interpolation still led to the preservation of major electronic patterns. We thus did not remove any false points before conducting RBF interpolation of electronic features in our studies. Importantly, the interpolation suggests that the underlying dimensionality of electronic attributes on MEMS is much smaller than that of MEMS itself (as a matrix or RGB image) and likely in the same order as the number of atoms. This is not intuitively surprising, as electronic features on a molecular surface spread over domains comparable with the size of an atom.

**Fig. 4:**
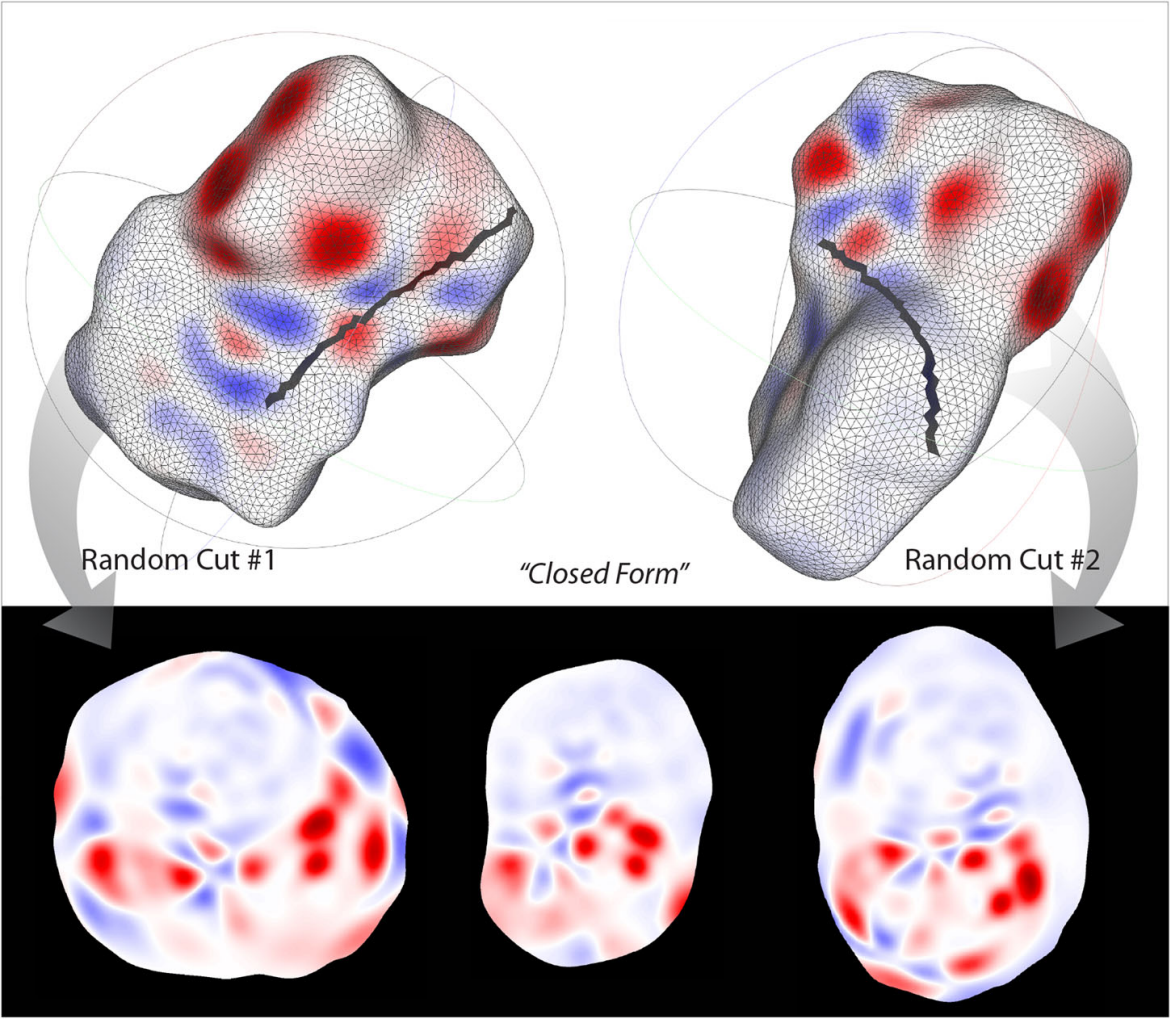
MEMS from cut and closed molecular surface. Two embeddings (F^2^) of the same surface manifold of tolfenamic acid are being cut. The middle embedding is of the uncut manifold.

Nonetheless, to minimize the information loss due to false positives and negatives, we attempted to cut a molecular surface by removing the connectivity between surface vertices along the geodesic between two vertices on the surface. The vertices along the cutting line are forced to become the boundary points on the embedding. Figure 4 shows MEMS of the same Hirshfeld surface (F^2^) of tolfenamic acid that is randomly cut; both cut MEMS have no embedding point in a wrong neighborhood (with respect to the cut surfaces). In comparison, the MEMS of the uncut surface (“closed”) has about 40% false points. Nonetheless, false negativity of the points along the cutting line is entailed. We thus posit that two or more cut MEMS be combined to mitigate ambiguity and truthfully represent a molecule. Note that for the molecules in the solubility prediction, cutting was done not randomly but between two surface points intercepted by principal axes of mesh points.

The matrix or image format of MEMS may be directly utilized in a machine learning model, e.g., by convolutional NNs (CNNs). However, the true dimensionality of a MEMS is much smaller than that of the embedding itself, as implied by Fig. 3. While MEMS is translation- and rotation-invariant, its orientation when placed on 2-D is random but should be orientation-invariant. We thus sought ways to further featurize and reduce the dimensionality of MEMS, among which the shape context used in computer vision^47^ was adopted and discussed herein.

### Shape-context featurization of MEMS

**Fig. 5:**
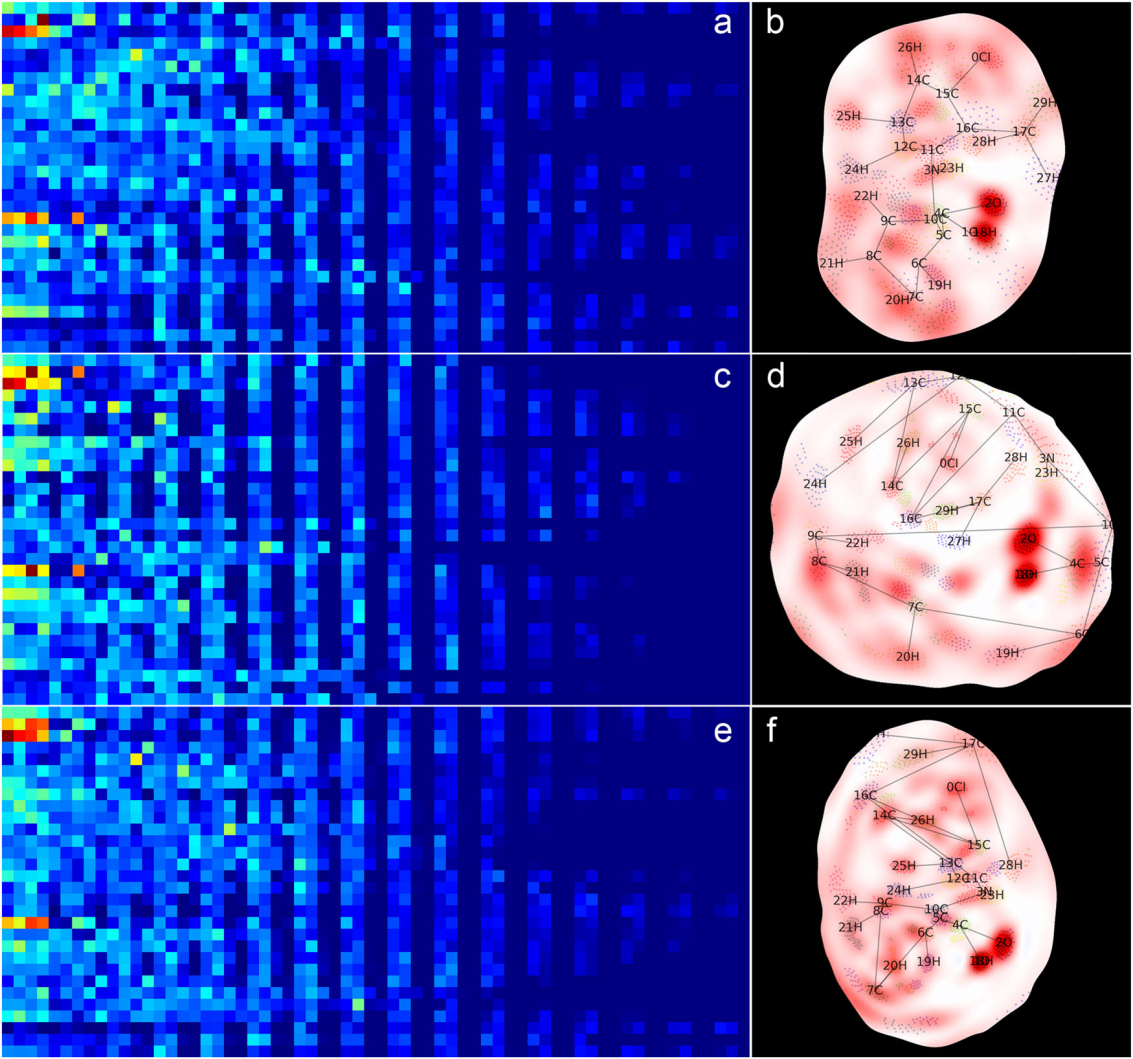
Shape-context matrices of MEMS derived from the same surface manifold. Embeddings (**a**, **c**, and **e**) are derived from closed (**b**), and randomly cut (**d** and **f**) molecular surfaces, respectively. Key points are marked on the MEMS. The color scheme of the matrix plots varies from blue to green and to red as the bin value increases. The electronic values are electrophilic Fukui functions.

Shown in Fig. 5, a shape-context matrix consists of rows of key points, which are chosen as the closest surface vertices to the respective atoms of the molecule. When used in calculation, the absolute values of the respective electronic properties of the bins are processed. The three MEMS (F^-^) and corresponding shape-context matrices in Fig. 5 are derived from the same molecular surface (Fig. 4; with a different electronic property). Despite the MEMS being derived differently, their shape-context matrices appear highly similar. The similarity distances calculated by the Earth Mover’s Distance (EMD) algorithm^48^ are 1.44 (between Fig. 5a, c), 1.18 (between a and e), and 1.35 (between c and e). In comparison, the EMD between Fig. 2c, g is 1.70. The similarity suggests that shape context saliently captures the spatial distribution of electronic attributes on a molecular surface.

**Fig. 6:**
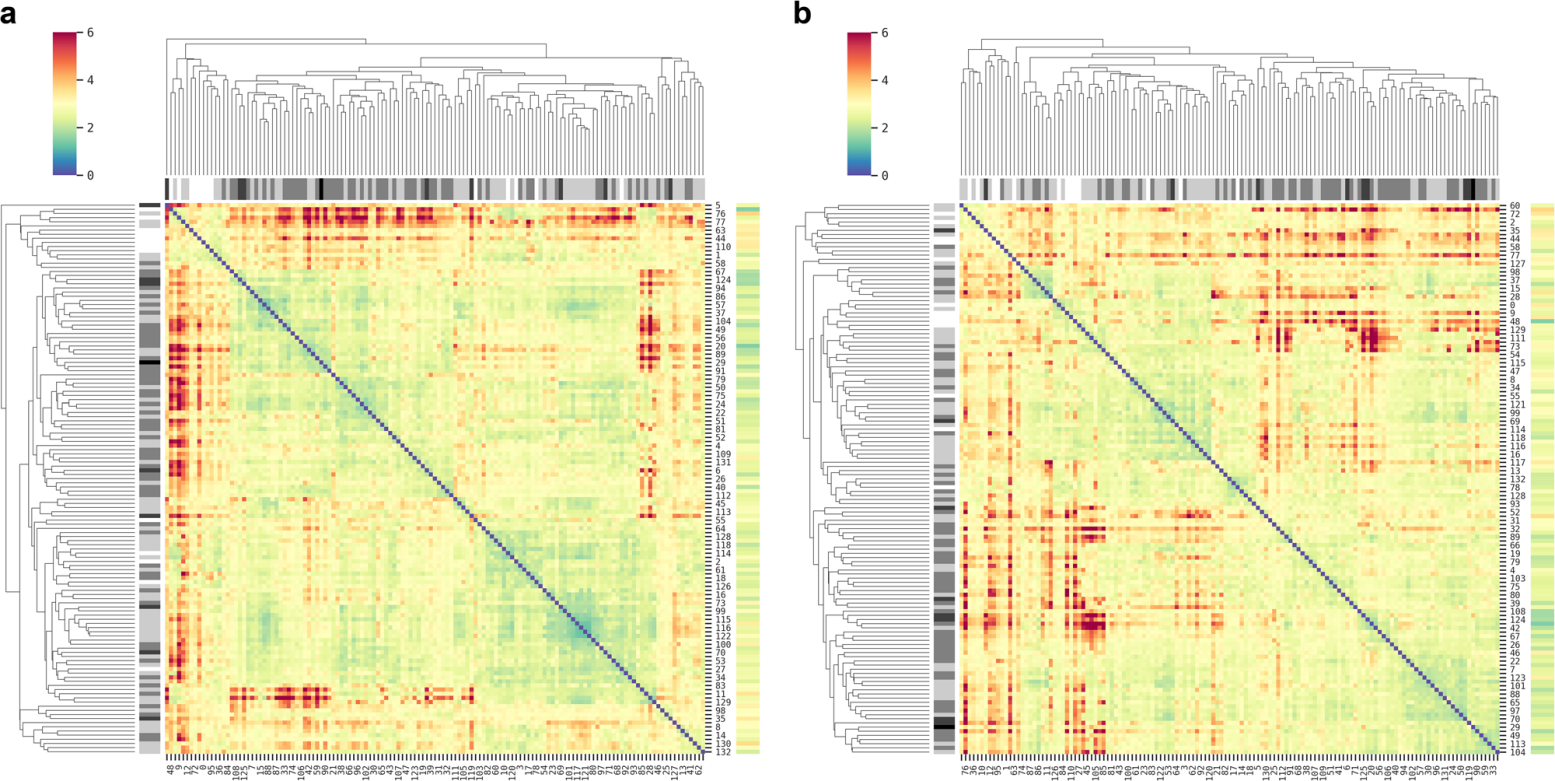
Heatmap and cluster analysis of EMD values between shape-context matrices. MEMS are of the negative ESP on *closed* (**a**) or *cut* (**b**) Hirshfeld surfaces of 133 molecules utilized in the deep-learning prediction of water solubility. Evenly indexed molecules are marked on the right and oddly indexed ones are marked on the bottom. The indices can be found in Table S1. The gray bar under each dendrogram is of solubility with white, light gray, mid-gray, dark gray, and black marking LogS > −2.0, > −4.0 and <= -2.0, > −6.0 and <= -4.0, >−8.0 and <= −6.0, and <−8.0, respectively. The color bar on the far right is of the averaged EMD of each molecule among close and its four cut MEMS, corresponding to the respective molecules by the rows.

To further examine manifold cutting on shape-context featurization, similarities between the feature matrices of Hirshfeld surfaces of the 133 molecules used in the solubility prediction were calculated. Figure 6 shows two comparing heatmaps of EMD values along with clustering dendrograms of the similarities. Additional heatmaps of positive ESP and Fukui functions can be found in Supporting Information.

**Fig. 7:**
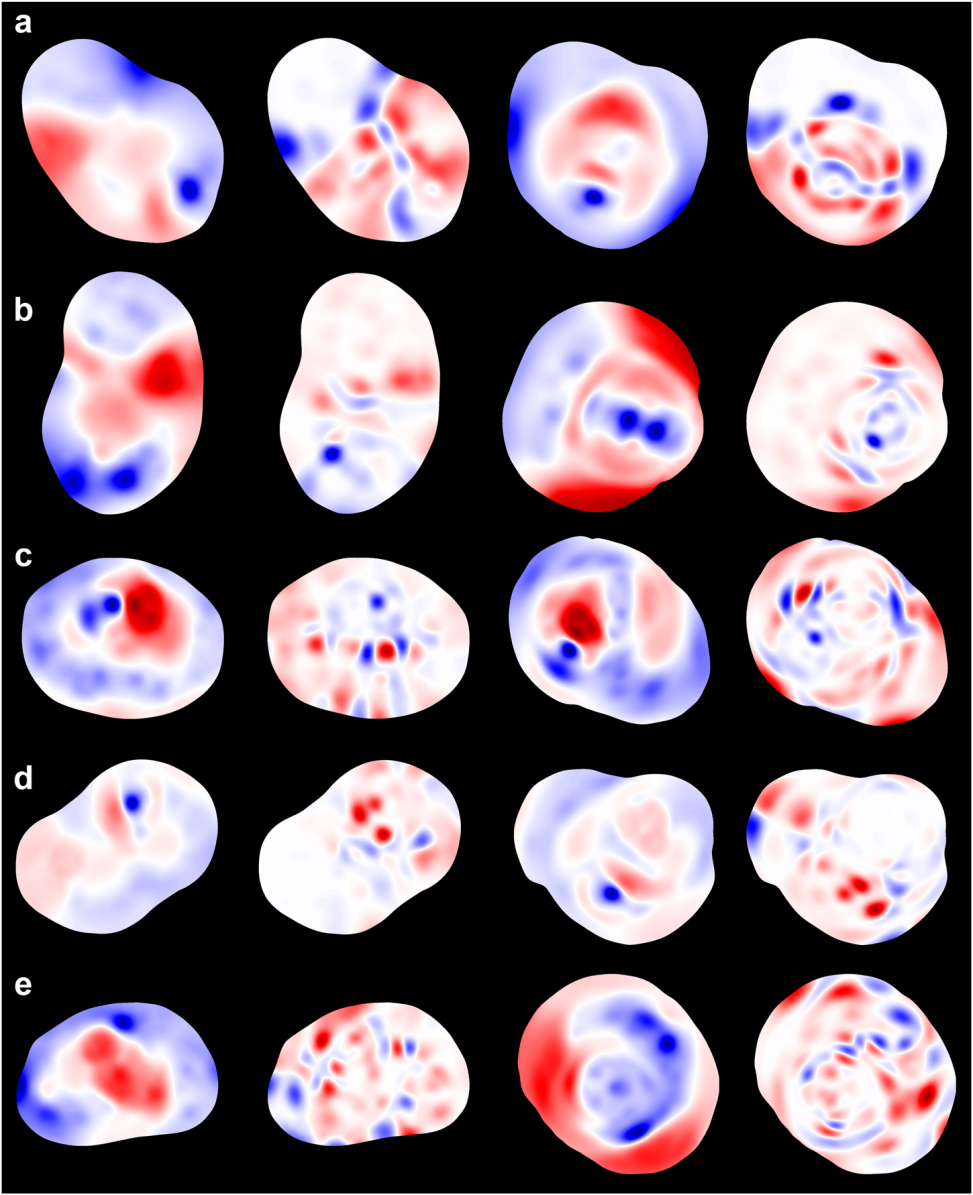
MEMS of selected molecules. ESP and F^2^ MEMS of four selected molecules used in the deep-learning prediction, acetaminophen (**a**), benzocaine (**b**), carbamazepine (**c**), flufenamic acid (**d**), and sulfisoxazole (**e**). Of each molecule, the first two are of closed (ESP and F^2^) and the other two of cut MEMS. The color scheme is the same as that in Fig. 2.

Several observations may be made by perusing the similarity maps. The averaged EMD values between the closed and four cuts of the same molecules are generally smaller than the values between molecules. The two heatmaps share similar patterns in general, suggesting that manifold cutting does not alter the overall differences among the molecules or introduce significant falsehood (i.e., false negatives on MEMS). Similarity values of Fukui functions (Figs. S3–6) are smaller than those of ESP, indicating that MEMS of Fukui functions are less dissimilar. However, given the much localized and richer patterns of Fukui functions as compared to ESP (e.g., Fig. 7), the setup to calculate EMD with 4 angular bins (and 12 radial ones) might not be discerning enough for Fukui functions, warranting further studies. As the molecules are largely differentiated and consistently distributed on the maps, the featurization scheme by shape context seems capable of retaining the electronic properties on the molecular surfaces. Interestingly, the clustering apparently bears no correlation with the respective solubility values. This may not be surprising as the EMD or similarity values can be regarded as low-dimensional (non-linear) projections of the MEMS. The full shape-context features need to be considered to predict the solubility.

### Solubility Prediction by MEMS

Solubility is one of the essential physicochemical properties of molecules. Being a grand challenge in chemistry, predicting a molecule’s solubility has been attempted in many studies, ranging from empirical and data-driven models to thermodynamic evaluations and to computer simulations. Solubility is a property of solid state, determined by intermolecular interactions among the solute and solvent. Two solubility challenges were recently held with enthusiastic participations^49–52^. Various degrees of performance were achieved, but the space to improve still remains widely open^50^. Considering experimental errors in obtaining solubility data, one log unit between experimental and predictive values has been a widely-regarded bar of evaluation. Still, larger experimental errors and inter-laboratory variabilities are expected, compounding the difficulties in solubility prediction. We thus took advantage the four well-curated datasets from the two challenge calls^51,52^, and developed a neural network framework to evaluate the applicability of MEMS and shape-context matrices for solubility prediction. As solubility is a property of crystal, our initial attempt reported here was based on the manifold embeddings that were calculated of the drug crystals (i.e., Hirshfeld surfaces). We obtained crystal structures of 133 molecules from the datasets (Table S1) and conducted the deep learning with 13 hidden layers. Several representative MEMS are shown in Fig. 7; the full list of the MEMS can be found in Table S2.

The calculated MEMS (ESP and F^2^) shown in Fig. 7 corroborate that the embeddings preserve the essential electronic information, both spatial distributions and numerical scales, on a particular molecular surface. Compared with the closed counterparts, the cut MEMS retain extra details of electronic values from a molecular surface but still share the major electronic features. The red spots on the ESP MEMS mark electron-concentrated and the blue indicate electron-deprived regions. Additionally, the red spots on the F^2^ MEMS are electron-hungry and the blue electron-donating. A local region with larger ESP or F^2^, either red or blue, is associated with greater contributions to intermolecular interactions, including hydrogen bonding and aromatic stacking^25,26^. A hotspot of ESP is typically echoed by larger values of Fukui functions, which are more localized and featured. The electronic attributes on a molecular surface and thus MEMS not only determine the strength of interacting with another molecule, but the local, spatial variations also control the locality of molecular interactions. From the machine-learning perspective, the distinctive patterns of the MEMS, especially with combination of both ESP and Fukui functions, contain the discerning power to represent different molecules.

**Fig. 8:**
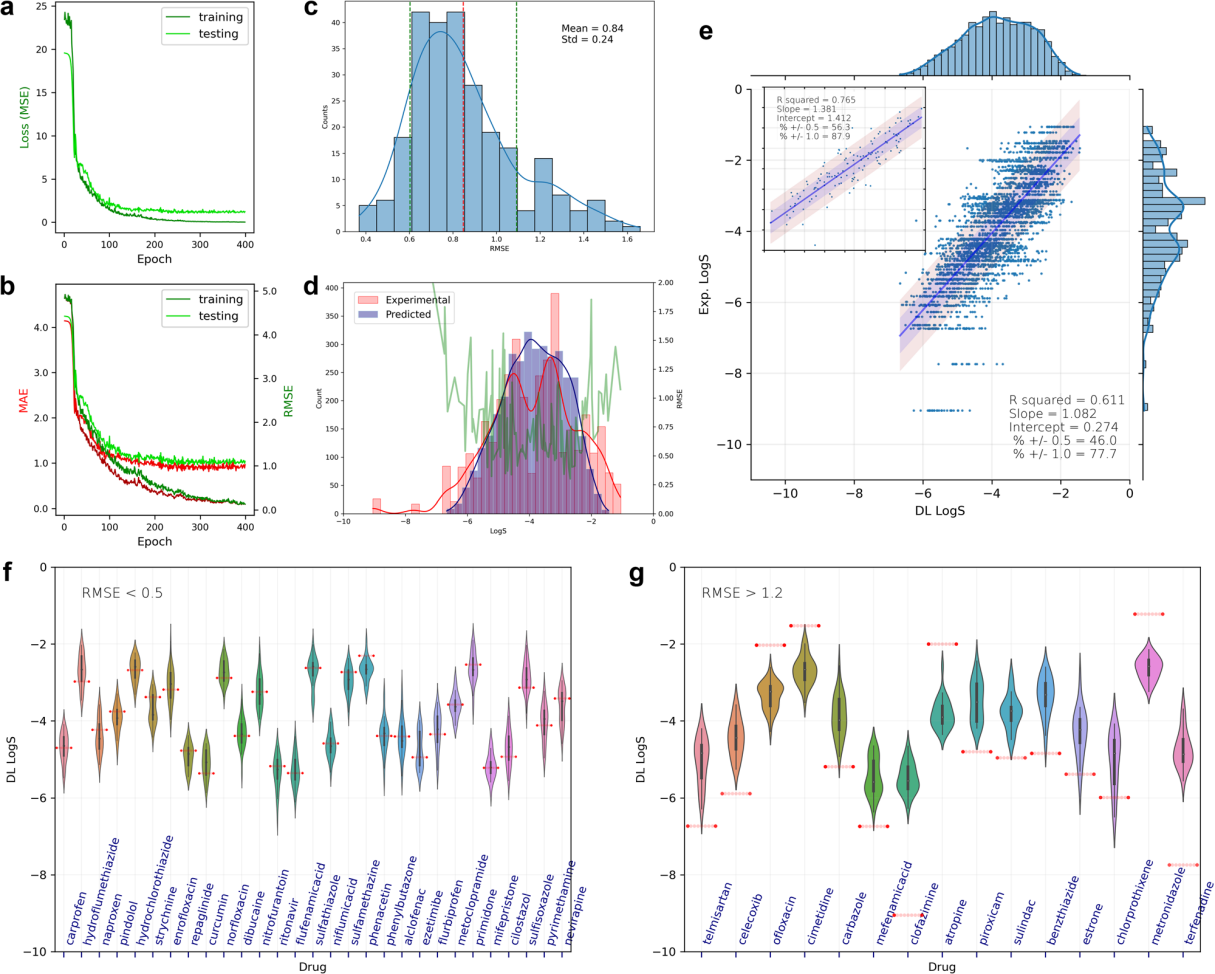
Deep-learning results by cut Hirshfeld surface MEMS of 133 molecules. The results include prediction MSE (mean-squared error), MAE (mean absolute error), and RMSE (root-mean-squared error) between the predicted and experimental values of a representative learning run (**a** and **b**), RMSE distribution of cross validations (CVs) (**c**), distributions of predicted and experimental values and RMSE values of each molecule (**d**), linear fitting of predicted vs experimental values along with their respective distributions and **e** and average predicted vs experimental values (e; inset), best-predicted molecules (RMSE < 0.5) with violin plots of predicted values and red dots marking corresponding experimental values (**f**), and worst-predicted molecules (RMSE > 1.2; **g**). Solubility is in logarithm unit.

With shape-context matrices of four cut MEMS of each molecule, one set of deep-learning results by 9:1 splitting of the 133 molecules as training and testing datasets is shown in Fig. 8. The training and testing losses of a representative run (Fig. 8a, b) illustrate a typical deep learning process seen among other runs in this study. Out of the 256 CVs, each molecule was predicted at least 16 and at most 44 times, shown in Fig. 8e. The R-squared value (R^2^) is 0.61 and about 46.0% and 77.7% of the predicted values fall within half and one logarithm unit from their respective experimental values. When the predicted values from the CVs are averaged of each molecule and plotted against the experimental data (Fig. 8e; inset), R^2^ increases to 0.76. The distribution of RMSE by the CVs is shown in Fig. 8c with the average of 0.84 between two extremes around 0.4 and 1.6. RMSE of each molecule predicted out of the CVs is shown in Fig. 8d, superimposed with the distributions of the predicted and experimental values (which are also showed in Fig. 8e on the top and right axes, respectively). The results suggest that the prediction performance depends on which molecules were included in the testing dataset (and, reciprocally, in the training). As shown in Fig. 8f, the “best” molecules typically have their experimental values between −2.0 and −6.0 where most training data points reside (also shown in Fig. 8d). At the two tails of the data distribution (> −2.0 or < −6.0), fewer experimental points were available; only two molecules have solubility smaller than −7, clofazimine (−9.05) and terfenadine (-7.74). These two molecules were poorly predicted (Fig. 8e, g). Among the 15 “worst” molecules, 10 seem to have their solubility values outside or bordering the −2.0 and −6.0 range. Note that there are two datasets in the 2019 Solubility Challenge and the second one has much larger experimental uncertainties (>0.6 as compared to <0.2 of the first dataset)^50,51^. We had 16 molecules taken from the second dataset (Table S1) and 4 of them had poor predictability (Fig. 8g), including clofazimine, terfenadine, chlorprothixene (−5.99), and telmisartan (−6.73), which all reside at the insoluble end of the solubility distribution.

The relatively wide distributions of predictive RMSE values imply the sensitive nature of the deep learning model to the small size of training data. We further took 95% or 126 of 133 molecules as the training set and conducted deep learning (Figures S8a–c). The average RMSE of the CVs decreased from 0.84 to 0.60; R^2^ of the linear correlation increased from 0.61 to 0.78 with 62.9% of the molecules within half logarithmic unit. In addition, the RMSE values of the molecules show similar connections with the distribution of the training data (Figure S8b). Again, the two most insoluble molecules, clofazimine and terfenadine, had the worst predicted RMSE (>2.0). We thus took another attempt with the same configuration but removed clofazimine and terfenadine from the whole dataset (Figures S8d–f). The average RMSE from the CVs decreased to 0.57 and R^2^ of the linear correlation increased to 0.79. By further excluding all 16 molecules that were taken from the second set in the 2019 Solubility Challenge, which have experimental uncertainties of 0.6 or greater^50,51^, we utilized 95% of the 117 molecules for deep learning (Figures S8g–i). The averaged RMSE is improved to 0.55 and R^2^ becomes 0.80 with 66.0% of molecules within half logarithmic unit respectively from their experimental values. When we tested using the 117 molecules as the training and the 16 molecules as the testing dataset, the linear correlation between the predicted and experimental values is poor, indicated by R^2^ of 0.04 (Figure S9). RMSE of 7 molecules are greater than 1.0 with the average RMSE of the 16 molecules being 1.92. The two most insoluble molecules have the largest prediction errors. The results suggest that prediction performance by deep learning is greatly affected by the distribution of the training data. In our case, there was no molecule with solubility < −7.0 in the training set. The large experimental uncertainties (>0.6) of the 16 molecules also made the prediction difficult to evaluate. Moreover, we utilized the closed MEMS by the same deep learning model that had 13 hidden layers. Interestingly, the prediction performance is almost identical to the results obtained by using four cut MEMS (Figure S10a, b vs. 8e and S8c), indicating that the salient electronic information pertinent to solubility is still largely retained by closed MEMS.

In the above exercises, Hirshfeld surface was used as molecular manifold, which is generated from the partitioning of electron densities in the crystal structure of a molecule^44^. We then employed the electron-density iso-surface of a single molecule to compute MEMS and derive shape-context matrices for solubility prediction. The conformer of each molecule remained the same as that extracted from the respective crystal structure (with only hydrogen atoms optimized). The iso-surface MEMS of the same molecules from Fig. 7 show great similarities with the Hirshfeld surface counterparts (Figure S7). The predicted outcomes are almost identical to the results of using Hirshfeld surfaces. When using four cut MEMS derived from the iso-surface of each molecule, R^2^ is 0.63 with 49.1% and 79.9% of the molecules within half and one logarithmic unit of experimental values (Figure S10c), slightly better than the Hirshfeld model (Fig. 8e). When the data splitting went from 90:10 to 95:5 between training and testing, the R^2^ value became 0.78 with 69.2% and 92.2% of the molecules within half and one logarithmic unit of experimental values (Figure S10d). MEMS of closed iso-surfaces were tested as well and similar solubility prediction results were obtained (Fig. S10e, f). Lastly, we tested fully optimized single molecules for generating iso-surface MEMS and deep learning of solubility. Prediction results of the fully optimized 133 molecules (Fig. S11) resemble those by Hirshfeld surfaces or electron-density iso-surfaces that are generated from the conformations extracted from respective crystal structures. Table 1 summarizes the deep-learning predictions.

**Table 1:** Deep Learning Performance of the Solubility Challenges Dataset by MEMS

MEMS	Data Size & Splitting	Conformer	MAE	RMSE	R^2^	% ± 0.5 & ± 1.0	R^2^ (average)
HS; 4 cuts	133; 90%	Partial	0.67 ± 0.18	0.84 ± 0.24	0.61	46.0; 77.7	0.76
HS; 4 cuts	133; 95%	Partial	0.49 ± 0.26	0.60 ± 0.33	0.78	62.9; 89.6	0.88
HS; 4 cuts	131; 95%	Partial	0.47 ± 0.18	0.57 ± 0.21	0.79	62.8; 91.1	0.89
HS; 4 cuts	117; 95%	Partial	0.45 ± 0.21	0.55 ± 0.24	0.80	66.0; 90.9	0.90
HS; closed	133; 90%	Partial	0.68 ± 0.17	0.86 ± 0.23	0.61	45.5; 75.9	0.76
HS; closed	133; 95%	Partial	0.51 ± 0.25	0.64 ± 0.32	0.76	61.1; 88.1	0.87
ISO; 4 cuts	133; 90%	Partial	0.64 ± 0.19	0.81 ± 0.27	0.63	49.1; 79.9	0.78
ISO; 4 cuts	133; 95%	Partial	0.44 ± 0.27	0.57 ± 0.38	0.78	69.2; 92.2	0.88
ISO; closed	133; 90%	Partial	0.68 ± 0.18	0.87 ± 0.25	0.61	46.3; 77.2	0.73
ISO; closed	133; 95%	Partial	0.52 ± 0.26	0.67 ± 0.34	0.75	62.2; 86.6	0.85
ISO; 4 cuts	133; 90%	Full	0.67 ± 0.18	0.85 ± 0.26	0.61	46.4; 77.9	0.75
ISO; 4 cuts	133; 95%	Full	0.50 ± 0.26	0.63 ± 0.37	0.77	63.8; 90.7	0.85
ISO; closed	133; 90%	Full	0.69 ± 0.19	0.87 ± 0.25	0.60	45.2; 75.6	0.74
ISO; closed	133; 95%	Full	0.52 ± 0.23	0.64 ± 0.31	0.77	60.7; 88.6	0.86
ISO; 4 cuts	200; 90%	Full	0.74 ± 0.15	0.95 ± 0.19	0.56	42.7; 72.2	0.73
ISO; 4 cuts	200; 95%	Full	0.58 ± 0.19	0.72 ± 0.24	0.72	53.2; 83.8	0.84
ISO; closed	200; 90%	Full	0.73 ± 0.13	0.91 ± 0.16	0.60	41.6; 71.9	0.73
ISO; closed	200; 95%	Full	0.61 ± 0.18	0.77 ± 0.23	0.71	51.1; 80.9	0.81

MEMS were derived from cut or closed Hirshfeld surface (HS) or electron-density iso-surface (ISO) of single molecules that were partially optimized for hydrogen atoms based on the conformers extracted from respective crystal structures or fully optimized. In the deep learning, 13 hidden layers were utilized with the dropout rate of 0.2. Two data splitting ratios were tested to divide the dataset. The prediction metrics were collected by conducting 256 cross validations.

Overall, equivalent results of solubility prediction of the same molecules were obtained by the data-driven learning of their MEMS, regardless of whether Hirshfeld surface or iso-surface was used, whether the surface was cut or closed, and whether the molecules were fully, or partial optimized. We further tested 200 fully optimized molecules combined out of the two Solubility Challenges and utilized their iso-surfaces for deep learning. As shown in Table 1, compared with learning the smaller dataset of the 133 molecules, the prediction was slightly weakened (Figure S12). For instance, R^2^ by utilizing four cut MEMS with 90% or 95% of molecules used in training is 0.56 or 0.72; the corresponding values by the 133 molecules are 0.61 and 0.77. Based on the distributions of experimental values (Figure S13), the larger dataset seems to disproportionately have a few more data points at the poor solubility tail, which may contribute to the slight decrease in prediction performance.In the aforementioned predictions (Table 1), the same deep learning model was deployed, in which 13 hidden DeepSets layers were constituted. It resulted in about 20 million training parameters in the neural networks. Because MEMS fully encodes the quantum information of a molecule pertinent to molecular interactions, it might be feasible to use fewer hidden layers and approximate the latent function between MEMS and solubility. We thereby attempted using two and one hidden layer for learning the four cut and one closed MEMS, respectively, of the same molecules from the Solubility Challenges (Figures S14–17). The prediction metrics, summarized in Table 2, are highly similar to and even slightly better than those by the deep-learning model (Table 1). For example, the best RMSE are 0.57 and 0.72 when predicting the 133 and 200 molecules, respectively, by the deep learning. Correspondingly, the values become 0.53 and 0.67 by the shallow learning. The differences in the prediction metrices between using 4 cut and one closed MEMS are trivial, again similar to the differences observed in the deep learning. It is worth noting that the dropout rate was 0.75 in the shallow learning, compared to 0.2 in the deep learning. The number of training weights was significantly reduced. When one hidden layer was used to handle closed MEMS of iso-surfaces, 72,480 parameters were employed. Even when two hidden layers were used to process 4 cut MEMS, the number of weights was 683,380, significantly reduced from the 20 million by 13 hidden layers.

**Table 2:** Shallow Learning Performance of the Solubility Challenges Dataset by MEMS

MEMS	Data Size & Splitting	Conformer	MAE	RMSE	R^2^	% ± 0.5 & ± 1.0	R^2^ (average)
HS; 4 cuts	133; 90%	Partial	0.68 ± 0.18	0.84 ± 0.24	0.62	46.3; 77.9	0.77
HS; 4 cuts	133; 95%	Partial	0.48 ± 0.24	0.59 ± 0.32	0.80	65.3; 90.2	0.88
HS; closed	133; 90%	Partial	0.66 ± 0.16	0.83 ± 0.19	0.64	46.5; 77.6	0.79
HS; closed	133; 95%	Partial	0.46 ± 0.20	0.56 ± 0.24	0.82	64.2; 90.8	0.91
ISO; 4 cuts	133; 90%	Partial	0.61 ± 0.17	0.77 ± 0.24	0.69	50.7; 81.9	0.82
ISO; 4 cuts	133; 95%	Partial	0.43 ± 0.24	0.53 ± 0.32	0.82	69.5; 93.0	0.90
ISO; closed	133; 90%	Partial	0.64 ± 0.16	0.81 ± 0.23	0.65	48.6; 79.4	0.79
ISO; closed	133; 95%	Partial	0.44 ± 0.20	0.54 ± 0.25	0.82	67.1; 90.8	0.90
ISO; 4 cuts	133; 90%	Full	0.65 ± 0.16	0.80 ± 0.21	0.67	47.3; 79.1	0.81
ISO; 4 cuts	133; 95%	Full	0.47 ± 0.21	0.57 ± 0.27	0.82	64.7; 91.1	0.91
ISO; closed	133; 90%	Full	0.64 ± 0.16	0.79 ± 0.20	0.66	47.5; 79.8	0.81
ISO; closed	133; 95%	Full	0.46 ± 0.22	0.56 ± 0.27	0.82	67.4; 90.2	0.92
ISO; 4 cuts	200; 90%	Full	0.71 ± 0.14	0.89 ± 0.18	0.62	43.4; 75.1	0.76
ISO; 4 cuts	200; 95%	Full	0.54 ± 0.18	0.67 ± 0.24	0.77	56.7; 86.4	0.88
ISO; closed	200; 90%	Full	0.75 ± 0.13	0.92 ± 0.16	0.59	39.1; 70.9	0.73
ISO; closed	200; 95%	Full	0.57 ± 0.16	0.69 ± 0.19	0.76	52.3; 84.1	0.88

MEMS were derived from cut or closed Hirshfeld surface (HS) or electron-density iso-surface (ISO) of single molecules that were partially optimized for hydrogen atoms based on the conformers extracted from respective crystal structures or fully optimized. In the deep learning, 2 hidden layers were utilized for using 4 cut MEMS and 1 hidden layer was utilized for using closed MEMS with the dropout rate of 0.75. Two data splitting ratios were tested to divide the dataset. The prediction metrics were collected by conducting 256 cross validations.

**Fig. 9:**
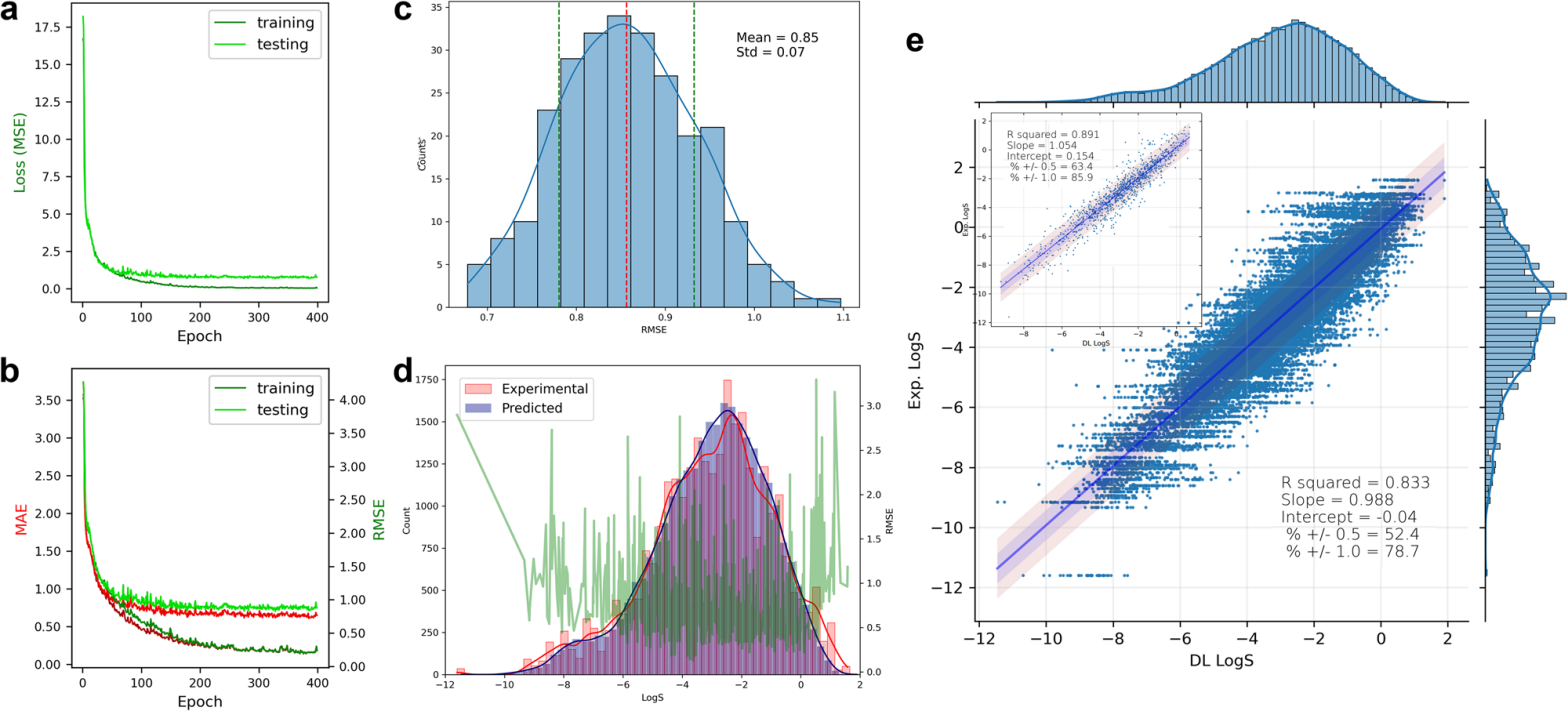
Deep-learning results by cut Hirshfeld surface MEMS of ESOL. The results include prediction MSE (mean-squared error), MAE (mean absolute error), and RMSE (root-mean-squared error) between the predicted and experimental values of a representative learning run (**a** and **b**), RMSE distribution of cross validations (CVs) (**c**), distributions of predicted and experimental values and RMSE values of each molecule (**d**), linear fitting of predicted vs. experimental values along with their respective distributions and **e** and average predicted vs experimental values (e; inset).

Finally, we conducted both shallow and deep learning of solubility prediction using the ESOL dataset^53^. It has experimental values of 1128 molecules and is often used as a benchmarking dataset in machine learning studies. Figure 9 shows deep-learning prediction results of using four cut MEMS derived from the electron-density iso-surface of each molecule at 90:10 split of the dataset. In a typical run, the training metrics vs epoch (Fig. 9a, b) suggest that the model converged quickly and the correlation linearity between predicted and experimental values (Fig. 9e) was attained reasonably well. Interestingly, the RMSE of individual molecules (Fig. 9d) were not as much affected by the distribution of the experimental data as what is shown in the prediction of Solubility Challenges (e.g., Fig. 8d). Using closed MEMS and splitting the data at 95:5 were also attempted (Figures S18–S24). Prediction results of the deep- and shallow-learning are summarized in Table 3. Our best RMSE value averaged over 256 CVs is 0.73 by 13 hidden layers and 0.72 by one hidden layer. The R^2^ of the predicted versus experimental values is 0.88 (by both deep and shallow) and that by the averaged predicted values is 0.91 (deep) or 0.93 (shallow). Overall, both the deep- and shallow-learning approaches achieved almost identical performance.

**Table 3:** Deep and Shallow Learning Performance of the ESOL Dataset by MEMS with Comparison of the State-of-the-Art (SOTA) Metrics

MEMS	Hidden Layers	Data Splitting	MAE	RMSE	R^2^	% ± 0.5 & ± 1.0	R^2^ (average)
ISO; 4 cuts	13	90%	0.63 ± 0.05	0.85 ± 0.07	0.83	52.4; 78.7	0.89
ISO; 4 cuts	13	95%	0.58 ± 0.07	0.78 ± 0.10	0.86	55.0; 81.8	0.91
ISO; closed	13	90%	0.60 ± 0.06	0.81 ± 0.09	0.85	53.7; 80.7	0.89
ISO; closed	13	95%	0.55 ± 0.07	0.73 ± 0.10	0.88	56.2; 83.8	0.91
ISO; 4 cuts	2	90%	0.63 ± 0.05	0.83 ± 0.07	0.84	51.6; 79.5	0.89
ISO; 4 cuts	2	95%	0.58 ± 0.08	0.76 ± 0.10	0.86	53.8; 82.2	0.91
ISO; closed	1	90%	0.61 ± 0.04	0.81 ± 0.07	0.85	52.4; 80.2	0.90
ISO; closed	1	95%	0.55 ± 0.07	0.72 ± 0.10	0.88	56.5; 84.5	0.93
D-MPNN (Directed Message Passing NN)^4^				0.97			
GeoGNN (Geometry-based GNN)^59^				0.80			

MEMS were derived from electron-density iso-surface (ISO) of fully optimized single molecules. In deep learning, 13 hidden layers were utilized, and in shallow learning, 2 and 1 hidden layer were utilized when using 4 cut and closed MEMS, respectively. Two data splitting ratios were tested to divide the dataset. The prediction metrics were collected by conducting 256 cross validations.

## Discussion

Chemical learning requires several traits to forge a robust prediction model that can approximate the latent function intended to capture. Ideally, the number of data points in the training set is sufficiently large to cover the sub-chemical space where the function resides. The quality of the experimental data used in training should be sound and well curated. The learning architecture needs to be cleverly designed to weed out noises and uncover the salient connections among input features, facilitated by the cost function and backpropagation. More importantly, the input features that describe or represent a molecule should carry expressive and discerning information that utilizes the output data to guide the approximation of the latent function.

Many schemes of molecular representation are developed from the conventional ball-and-stick notion of a molecule. In principle, if such a description scheme can fully differentiate each molecule (by uniquely projecting in an orthogonal hyperspace), the latent function between the input features and intended property should exist and the one-to-one relationship may be inferred by fitting training data. Given the underpinning nature of molecular interactions that is governed by the electronic structures of interacting molecules, using conventional molecular descriptions in a machine learning exercise may result in a causal function that is too complex to develop with a suitable machine learning model, as well as too high-dimensional to infer, exacerbating the COD and over-fitting the training data. Remediation requires multiple dimensionality reductions steps^54^, which, nonetheless, coarse-grains the molecular input and downgrades the model to discern molecules for prediction.

Facing the challenges, one feasible solution is to encode the quantum chemical information of a molecule as input to predict molecular interactions and pertinent properties. Ostensibly, this could ease the complexity by molecular descriptors. As the electronic structure and attributes of a molecule are well-defined and readily computable at various accuracy levels by quantum mechanical methods, it is viable to deploy electronic features as molecular representation for machine learning. Yet, it is difficult to directly employ electron densities and associated quantities as they are dispersed, un-structured, and dependent on rotation and translation of the molecule. Most of current efforts center on augmenting molecular graphs with electronic quantities partitioned to individual atoms or chemical bonds, or both. Graph neural networks and variants are subsequently utilized as the supervised learning architecture to numerically infer the connection between an input graph and its conforming value of property.

Taking a different route, MEMS aims to encode quantum mechanical information on a molecular surface as molecular input in chemical learning. It is conceptualized to capture the inherent electronic attributes of a molecule that govern the strength and locality of intermolecular interactions it forms. Because electron densities and associated quantities are locally distributed around the nuclei of a molecule, the electronic properties on a molecular surface or manifold are routinely utilized in understanding molecular interactions, including the local hardness and softness concepts within the framework of CDFT^25,26^. To reduce the dimensionality of the electronic attributes on a surface and, equally important, to eliminate the degrees of freedom due to the positioning of the surface manifold, we resorted to manifold embedding to preserve the electronic quantities in a lower dimension by a stochastic neighbor embedding method^43^.

As illustrated in Figs. 2 and 7, the MEMS of electronic attributes maintain both values and their original spatial relationships on the molecular surfaces. The same can be said about the iso-surface MEMS (Figure S7). The 2-D embeddings are visually expressive and authentic; they encode the totality of the quantum chemical information of a molecule pertinent to its interactions. As our eyes are more perceptive to 2-D imageries, MEMS provides readily understandable cues of the electronic features enveloping the whole molecule. Critically, MEMS is independent of the orientation or rotation of a molecule; the invariance results from the distance-based neighborhood embedding of the surface manifold. Because a molecular surface is enclosed, a number of false positives and negatives with regard to the manifold neighborhood are generated on the resultant MEMS. To mitigate the loss of information, manifold cutting was attempted, leading to MEMS that have no false information in light of the cut manifold, as demonstrated in Fig. 4. While the manually introduced boundary along the cutting line is correctly reproduced on the cut MEMS, false negativity is inherited from the artificial boundary points of the cut surface. We posited that by using two or more cut MEMS of the same molecular surface, the inherent electron information of a molecule could be largely recovered especially by deep learning. As discussed below, nonetheless, even shallow learning of closed MEMS achieved a similar performance of solubility prediction to that by using multiple cut MEMS.

**Fig. 10:**
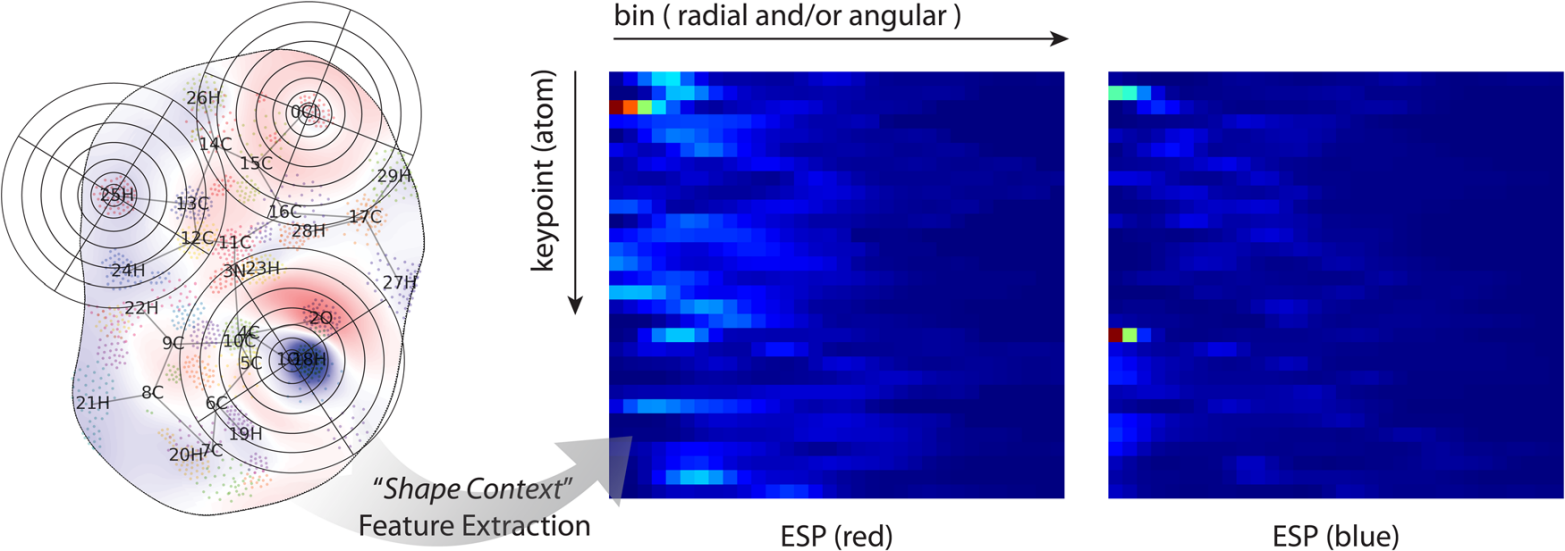
Shape context featurization scheme. Context bins are highlighted on three keypoints on MEMS (ESP; closed form) and the derived context matrices (1 angular and 32 radial bins) are shown.

The true dimensionality of the electronic attributes on MEMS is thus much smaller than that of the manifold embedding (when presented as image), comparable to the number of atoms (of a molecule). Our current attempt to seek the true dimensionality and thus featurize MEMS was enabled by the numerical shape context algorithm^47^. A 4 × 16 scheme is demonstrated in Fig. 5, where 4 angular and 16 radial bins are evaluated around a key point. The number of key points equates to the number of atoms and positioning of the key points is assigned by the closest surface points to the respective nuclei. A 1 × 32 scheme is also illustrated in Fig. 10. Compared to the dimensionality of an image (width × height × pixel depth), the dimensionality of a shape-context feature matrix (e.g., Fig. 5a) is significantly reduced and in line with the number of atoms. Additionally, a feature matrix is independent of positioning of MEMS (see Methods), further ensuring the electron information captured by the manifold embedding invariant of a molecule’s positioning or rotational degrees of freedom.

We applied the 4 × 16 shape-context matrices of a small but well-curated set of 133 molecules in a neural-network model of solubility prediction. DeepSets was chosen as the architecture to enable the permutation invariance of an input matrix^55^. The MEMS of each molecule embed several electronic properties, including ESP and Fukui functions. The prediction results (Table 1) support the feasibility by MEMS to encode and represent a molecule in machine learning. The prediction accuracy (e.g., RMSE of each CV or RMSE of each molecule) was determined by the data distribution of training molecules. Most of the 133 molecules have logarithmic solubility values between -2.0 and -6.0, which yielded the smallest RMSE. There are only two insolubility molecules (clofazimine and terfenadine) of solubility < −7.0. Their predicted values showed the largest errors. The quality of experimental values also affected the prediction performance, demonstrated by the prediction of the 16 molecules with experimental uncertainties > 0.6 (Figure S9). While the dataset used in this study is relatively small, the close matching between the distributions of experimental data and prediction accuracy (e.g., Fig. 8d), which is seen in every deep learning exercise conducted in this study, indicates the data-driven nature of machine learning. The sensible robustness of the prediction accuracy to the data distribution likely results from the inherent, quantitative connection by MEMS to solubility. The observation echoes the non-parametric nature of neural networks, which might be analogous to Gaussian Process (GP)^56^. The variance of testing data by GPs is governed by not only the variance of training data but also the covariance between the testing and training data^57^. This might explain the significant improvement in prediction when the relative portion of testing data became smaller (i.e., 95:5 vs. 90:10 of data splitting).

The prediction results even with such a small set of training data seem to support our aforesaid argument of the “domain distance” between a molecular representation and the property of interest. Because MEMS retains the local electronic values of a molecule and their spatial relationship, the causal function between MEMS and solubility is assumed straightforward and much simpler to infer by neural-network models. Comparable prediction outcomes were achieved between MEMS generated from the Hirshfeld surfaces or electron-density iso-surfaces of the molecules (Table 1). This suggests that a particular form of molecular surface may be irrelevant; it is the local electronic values and their spatial distribution uniquely defined by a molecule that matter. This argument is echoed by the similar prediction results when the fully optimized molecules were used to generate iso-surface MEMS for the solubility prediction. Note that the insensitivity of the conformational variations (i.e., partially vs fully optimized) to solubility prediction does not suggest that the property is independent of conformation but rather due to the lack of such training data (in our study). Additionally, while a larger training set could improve prediction accuracy, the improvement was not apparent when 200 fully optimized molecules were utilized, compared with the trials of the 133 molecules. The slight decrease in prediction outcome could result from a heavier tail on the poor solubility side of the experimental data distribution (Figure S13).

Our deep-learning model consisted of 13 DeepSets layers, which implement the self-attention mechanism to derive salient features from MSMS and tie with solubility. The layers also served to implicitly reduce the dimensionalities of the learned features under the guidance of training data. Using four cut MEMS of a molecule achieved no better prediction than using one closed MEMS, implying that a closed embedding encodes sufficient quantum information for generalizing the chemistry of solubility. More interestingly, the prediction results by using one or two hidden layers (Table 2) show almost identical or even slightly better performance metrics by using the same MEMS in learning the same dataset. Drastically fewer training weights were needed by the shallow learning models – e.g., 72,480 in inputting closed MEMS, compared to 19,199,812 in the deep learning. What is even more interesting is that a much higher weight dropout rate had to be used, 0.75, in the shallow learning to avoid immediate data over-fitting, whilst 0.2 was chosen for the deep-layer model. The equality in prediction and distinction in model configuration of the shallow- and deep-learning models have several implications. The foremost is that MEMS is highly expressive in capturing the electronic attributes on a molecular surface, allowing even one (hidden) neural network layer to infer the latent function of solubility prediction. The large dropout rate used by the shallow-learning models also suggests that the shape-context features are strongly correlated. This may not be surprising as electronic values in the angular and radial bins of a key point are mutually retained in the bins of another point as well. Such information redundance seems robust against the extensive dimensionality reduction by the 13 hidden layers in the deep-learning model, still enabling the generalization of the training data.

Although direct comparison may not be objective due to different methods and training datasets being used, our predicted results seem to outperform the three dozen attempts reported in the two Solubility Challenges^49,50^. Various machine learning models were employed, including Random Forest, Supporting Vector Machine, Gaussian Process, and neural networks. The size of training datasets ranged from 81 to 10,237 molecules. Among the top performers (Table S3), one participant trained with more than 2,000 molecules by RBF (radial basis function) had the best RMSE of 0.78 and R^2^ of 0.62 with 54% of molecules within half logarithmic unit from respective experimental values. Conversely, one of our best efforts achieved the RMSE of 0.67 with R^2^ of 0.77 by shallow learning of 4 cut MEMS of the 200 fully optimized molecules from the two Solubility Challenges (Table 2). While this comparison may superficial, the molecular representations used by the contestants were mostly based on structural features of molecules, which might contribute to the broad variations in prediction performance and difficulties of developing effective machine learning models, as we alluded earlier.

For our prediction of a much larger dataset, ESOL, our results seem to be competitive, if not the best, when compared with the current state-of-the-art predictions. The best RMSE reported in MoleculeNet is 0.58, which is produced by a graph convolution model, MPNN (message passing neural network)^58^. A more recent study that critically evaluates molecular representations, including fingerprints, descriptors, and graph neural networks, on several datasets of molecular properties achieved a better RMSE, 0.56, on ESOL by D-MPNN (directed MPNN)^4^. The remarkable RMSE likely results from data over-fitting because of the random split of the dataset used in training, which, subsequently, permits the same or similar structural scaffolds shared between the training and testing data^4^. By utilizing the scaffold split of the dataset to minimize the overlap of molecular scaffolds, the same study reports its best RMSE of 0.97^4^. The best RMSE on ESOL that is reported to date, 0.80, is achieved by a geometry-based GNN model and scaffold split of the dataset^59^. Noted in these GNN studies is the dataset typically split just three times, either randomly or based on scaffold. As shown in Table 3, both of our shallow and deep learning achieved better RMSE of 0.72 and 0.73, respectively. Our supervised learning is unaffected by the issue of random vs. scaffold splitting, which is inherent in graph representation of molecules. Interestingly, the RMSE of individual molecules remain largely insensible to the distribution of experimental data (e.g., Fig. 9d), much different from what is illustrated in our Solubility Challenge predictions, where more precise prediction is enabled around the peak of the data distribution (e.g., Fig. 8d). Under the lens of Gaussian Process^56^, the RMSE vs. data distribution suggests that the solubility data in ESOL likely bears much larger experimental errors than the data in Solubility Challenges. One possible cause could be the solubility values of many weak acids or bases in ESOL not corrected for pH^60^. Another interesting finding from the ESOL prediction is that the slopes of the prediction versus experimental values are close to 1.0 (Fig. 9e and S18-24), suggesting that our learning models of MEMS work equally well along the solubility range, albeit limited by the quality of the experimental data. The same finding can also be made from the Solubility Challenge predictions.

To conclude, we developed a new concept of molecular representation to preserve quantum information of electronic attributes in a lower-dimensional embedding. The idea originated from our earlier studies of evaluating molecular interactions with local hardness and softness quantities within the CDFT framework^25,26^. The electronic features extracted from MEMS seem to capture the totality of a molecule’s inherent capability to interact with another molecule, both the strength and locality. What we learned from our exercise of solubility prediction hints that MEMS is highly expressive in encoding quantum information of a molecule, as well as highly compressive under extensive dimensionality reduction without losing major electronic information. Furthermore, because MEMS carries no direct information of the underlying molecular structure but local electronic quantities at the boundary of a molecule, the concept could overcome the so-called issue of activity cliffs in predictive and generative learning, where a minor structural change results in a significant difference in the activity of interest^61^. As it undertakes the un-supervised learning of manifold embedding and quantum mechanical evaluation of electronic quantities within the framework of the HSAB principle and CDFT, MEMS is expected to ease the development of supervised chemical learning and lessen the challenges due to the COD and limited availability of chemical data.

## Methods

### Manifold embedding of molecular surface

While other types of molecular surfaces are processed similarly, Hirshfeld surfaces are mainly illustrated in this report. A triangulated Hirshfeld surface was generated by Tonto^62^ and the vertices were further optimized by isotropic meshing in MeshLab^63^. The mesh vertices were input to a C++ program developed in-house to produce 2-D points of MEMS. To generate an embedding, we implemented Neighbor Retrieval Visualizer (NeRV)^43^. The process optimizes the distances among embedding points to preserve the local neighborhood of surface vertices. Specifically, it is evaluated as the probability of vertex *j* in the neighborhood of vertex *i*^43^:

$${p}_{j{{{{{\rm{|}}}}}}i}=\frac{\exp (-{g}_{{ij}}^{2}/{\sigma }_{i}^{2})}{{\sum }_{k\ne i}\exp (-{g}_{{ik}}^{2}/{\sigma }_{i}^{2})}$$

where *g*_*ij*_ is the geodesic distance and *σ*_*i*_ is a hyperparameter to determine the neighborhood coverage for *i*. A similar probability is defined by the Euclidean distance between the points *i* and *j* on the lower-dimensional embedding:

$${q}_{j{{{{{\rm{|}}}}}}i}=\frac{\exp (-{d}_{{ij}}^{2}/{\sigma }_{i}^{2})}{{\sum }_{k\ne i}\exp (-{d}_{{ik}}^{2}/{\sigma }_{i}^{2})}$$

The cost functions consists of two weighted Kullback-Leibler (KL) divergences between the two probability distributions in order to balance false positive and negatives^43^:

$$\lambda {\sum }_{i=1}^{N}{\sum }_{\begin{array}{c}j=1\\ j\ne i\end{array}}^{N}{p}_{j{{{{{\rm{|}}}}}}i}\log \frac{{p}_{j{{{{{\rm{|}}}}}}i}}{{q}_{j{{{{{\rm{|}}}}}}i}}+(1-\lambda ){\sum }_{i=1}^{N}{\sum }_{\begin{array}{c}j=1\\ j\ne i\end{array}}^{N}{q}_{j{{{{{\rm{|}}}}}}i}\log \frac{{q}_{j{{{{{\rm{|}}}}}}i}}{{p}_{j{{{{{\rm{|}}}}}}i}}$$

The hyperparameter, $\lambda$, is to weight the two KL divergences; we found a value of 0.95 works well in our cases. In addition, *σ*_*i*_ is dynamically adjusted based on the input data (i.e., surface vertices) and the data density around each point, and compared to a “perplexity” hyperparameter^42,64^, which was identified to be 30 in our study.

Electronic properties on the molecule surface are then pointwisely transformed to the MEMS. The properties of single molecules, including electrostatic potential (ESP), nucleophilic Fukui function (F^+^), electrophilic Fukui function (F^-^), and dual descriptor of Fukui function (F^2^), were calculated by Gaussian 09 (Gaussian, Inc., Wallingford CT) at the level of B3LYP/6-31G(d’,p’).

In addition to Hirshfeld surface, iso-surfaces of electron densities were also utilized to generate MEMS and for deep learning. Based on the volumetric data of electron densities of a molecule that were computed to derive Fukui functions, an iso-surface was calculated at 0.002 a.u. by the marching cubes algorithm in scikit-image^65^.

### Shape-context featurization of MEMS

To featurize MEMS for chemical learning, we developed a numerical method based on the shape context concept^47^. Shown in Fig. 10, a feature matrix consists of rows of key points, which are the closest surface vertices to the respective atoms of the molecule in 3-D (denoted by atom indices on the figure). The intensities surrounding a key point on a MEMS image are spatially separated in predetermined bins along the radial direction. Each radial bin may be further divided into angular bins, where the angular direction is calculated against the geometric center to allow the rotational invariance of the feature matrix. When used in deep learning, it is the originally calculated numbers of the respective electronic properties that are processed. In Fig. 5, each row in the feature matrices comprises 16 radial bins, each of which has 4 angular bins; on the other hand, there are 32 radial bins in Fig. 10.

### Solubility prediction by neural networks

Our shallow- and deep-learning effort selected 133 molecules out of 218 ones combined out of the First and Second Solubility Challenges^51,52^. Selection of the molecules was first limited to those with one molecule in the asymmetric unit (i.e., Z’ = 1), as well as availabilities of crystal structures. The selected molecules and respective crystal REFCODEs are listed in Table S1. Hirshfeld surfaces of the crystal structures of these molecules were calculated, and further dimensionality-reduced to manifold embeddings. Respective electronic properties (electron density, ESP, Fukui functions, and Fukui potentials) were evaluated of the single molecules with the conformations extracted from respective crystals, on which only the positions of hydrogen atoms were optimized. Electron-density iso-surfaces of the 133 molecules were also generated from the same conformations and, separately, from the fully optimized single molecules. Finally, 200 out of the 218 single molecules were fully optimized and utilized to generate iso-surfaces and electronic attributes. The excluded 18 molecules either had no reliable solubility values (10 molecules) or encountered difficulties in optimization (including 3 iodine compounds). In addition to predicting the molecules from the Solubility Challenges, a larger dataset, ESOL^53^, was evaluated as well. Of the 1128 molecules, 20 were excluded due to quantum mechanical computation difficulties by the basis set used in molecule optimization. The 20 molecules included 16 with iodine and 4 with sulfur atom. In general, evaluation of one molecule, including electronic calculation, MEMS derivation, and shape-context featurization, took about 10 min by a 20-core Intel 64-bit CPU.

Feature matrices were then derived by the shape context approach and used as the input for deep learning. The input of each molecule consisted of several feature matrices. DeepSets was adopted as the architecture of deep learning^55^; self-attention was used as the sum-decomposition that is demonstrated in Set2Graph^66^.

**Fig. 11:**
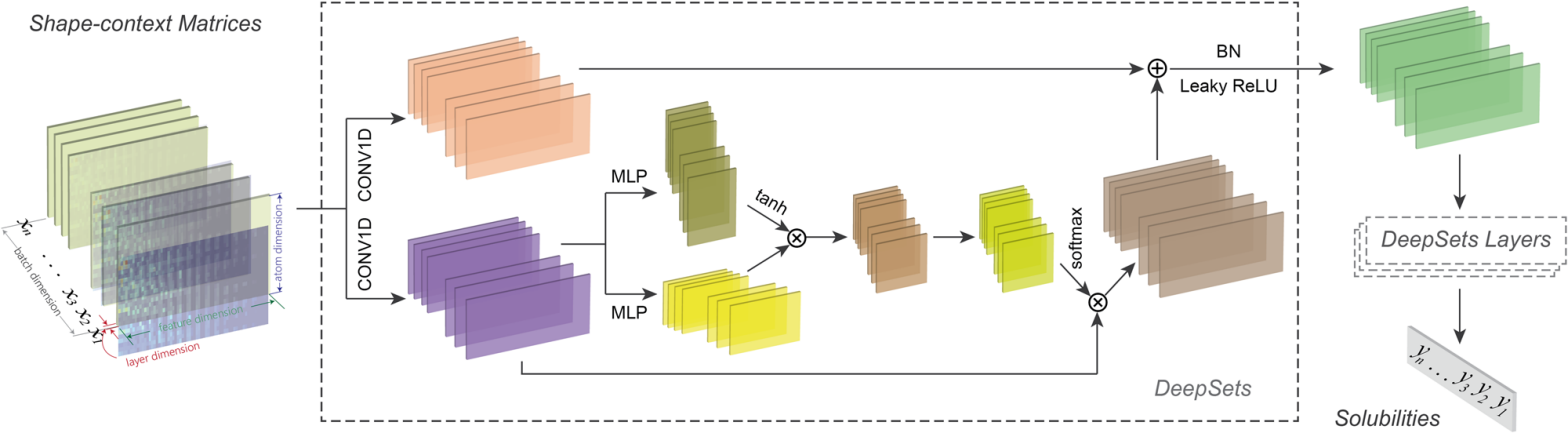
Deep learning model of solubility prediction with MEMS features. MEMS shape-context matrices are as input and DeepSets is adopted as the deep learning architecture. The input consists of a batch of molecules, each of which combines several MEMS feature matrices of electronic properties. Multiple DeepSets layers are utilized, leading to the output.

Illustrated in Fig. 11, the attention architecture is described as follows:

$$\Sigma (X)={{{{{\rm{softmax}}}}}}(\frac{\tanh ({f}_{1}\left(X\right))\cdot {{f}_{2}\left(X\right)}^{T}}{{d}^{1/2}})\cdot X$$

where *X* is the input set of MEMS features, *d* is the feature dimension of *X* divided by a predetermined number (typically 10), and ${f}_{1}$ and ${f}_{2}$ are the query and key functions of self-attention, which are implemented by MLP or multilayer perceptron. Notably, the self-attention mechanism is permutation invariant and is widely used to capture the intra-correlations of the input features. Additionally, regularization of each DeepSets was done by batch normalization (BN) and Leaky ReLU; weight decay and dropout (typically set at 0.2) were also considered in the PyTorch optimizer (Adam) to further mitigate model overfitting. The learning rate was set at 0.0001. MSE loss was chosen as the cost function.

As the total number of molecules in our solubility dataset was small, 256-fold cross-validations (CVs) were conducted to extensively evaluate the performance of our supervised learning methods. In each CV, the molecules were randomly split by 90:10 or 95:5 as training and testing datasets and each training was repeated 64 times with re-initialized training parameters. The repetition was necessitated as gradient descent used in the backpropagation could only lead to a local minimum. The prediction values by the repetition with the lowest loss were then recorded. Each molecule had 5 layers of electronic properties (electron density, positive and negative ESP, nucleophilic and electrophilic Fukui functions) and each layer had 64 shape-context bins per atom. Note that the electron density shape-context matrix was excluded from the input to the neural networks when iso-surface MEMS was utilized. Combining the electronic layers led to 1,280 (for Hirshfeld surface) or 1,024 (iso-surface) input features per atom. When four cuts of MEMS per molecule were combined as input, 13 DeepSets layers were used with the number of features as [1792, 1792, 1280, 1280, 640, 640, 320, 160, 80, 40, 20, 10, 4], resulting in a total of 22,910,474 training parameters (Hirshfeld surface) or 21,943,284 (iso-surface). Closed MEMS were also utilized as input, and the number of input features became 320 (Hirshfeld surface) or 256 (iso-surface) per atom. The same 13 DeepSets layers were adopted as well, with a total number of 19,342,394 training parameters (Hirshfeld surface) or 19,109,812 (iso-surface). On average, it took about 3-4 h to complete one CV with 64 repeats on an Nvidia A10 or A100 GPU.

Moreover, shallow learning with fewer DeepSets layers was conducted. For handling four cuts of MEMS, two hidden layers, [256, 128], were utilized, leading to a total number of 864,138 training parameters (Hirshfeld surface) or 683,380 (iso-surface). When closed MEMS were input, one hidden layer, [128], was employed with a total number of 92,070 training parameters (Hirshfeld surface) or 72,480 (iso-surface). A much greater dropout rate, 0.75, was used in the shallow learning to avoid over-fitting of the training data. On average, it took about 10–20 min to finish one CV.

### Supplementary information

Supporting Information

## Data Availability

The experimental solubility data used in this study were obtained from respective research papers cited in this work. The data that support the findings of this study, including input and output files of electronic calculations, manifold embeddings and shape-context matrices, and deep-learning output files, are available on request from the corresponding author (TL). Our data are digitally stored locally and by a centralized storage facility in Purdue Rosen Center for Advanced Computing.

## References

[1] Weininger D (1988). Smiles, a chemical language and information-system .1. Introduction to methodology and encoding rules. J. Chem. Inf. Comput. Sci..

[2] Rogers D, Hahn M (2010). Extended-connectivity fingerprints. J. Chem. Inf. Model.

[3] Jiang D (2021). Could graph neural networks learn better molecular representation for drug discovery? A comparison study of descriptor-based and graph-based models. J. Cheminform..

[4] Yang K (2019). Analyzing learned molecular representations for property prediction. J. Chem. Inf. Model.

[5] Bellman R. E. *Adaptive Control Processes*. Princeton University Press (1961).

[6] Hughes GF (1968). On mean accuracy of statistical pattern recognizers. IEEE Trans. Inf. Theory.

[7] Randic M (1991). Orthogonal molecular descriptors. N. J. Chem..

[8] Racz A, Bajusz D, Heberger K (2019). Intercorrelation limits in molecular descriptor preselection for Qsar/Qspr. Mol. Inf..

[9] Thanikaivelan P, Subramanian V, Rao JR, Nair BU (2000). Application of Quantum chemical descriptor in quantitative structure activity and structure property relationship. Chem. Phys. Lett..

[10] Rupp M, Tkatchenko A, Muller KR, von Lilienfeld OA (2012). Fast and accurate modeling of molecular atomization energies with machine learning. Phys. Rev. Lett..

[11] Matta CF (2018). Molecules as networks: a localization-delocalization matrices approach. Comput Theor. Chem..

[12] Bader RFW (1985). Atoms in molecules. Acc. Chem. Res.

[13] Bader RFW (1998). A bond path: a universal indicator of bonded interactions. J. Phys. Chem. A.

[14] Kneiding H (2023). Deep learning metal complex properties with natural quantum graphs. Digital Discov..

[15] Glendening ED, Landis CR, Weinhold F (2012). Natural bond orbital methods. Wiley Interdiscip. Rev. Comput Mol. Sci..

[16] Qiao Z (2022). Informing geometric deep learning with electronic interactions to accelerate quantum chemistry. Proc. Natl Acad. Sci. USA.

[17] Wu ZH (2021). A comprehensive survey on graph neural networks. IEEE Trans. Neural Netw. Learn Syst..

[18] Gavezzotti A (2005). Calculation of lattice energies of organic crystals: the pixel integration method in comparison with more traditional methods. Z. Krist..

[19] Cramer RD, Patterson DE, Bunce JD (1988). Comparative Molecular-Field Analysis (Comfa) .1. Effect of shape on binding of steroids to carrier proteins. J. Am. Chem. Soc..

[20] Li TL, Liu SB, Feng SX, Aubrey CE (2005). Face-integrated Fukui function: understanding wettability anisotropy of molecular crystals from density functional theory. J. Am. Chem. Soc..

[21] Li TL (2006). Understanding the large librational motion of the Methyl Group in Aspirin and Acetaminophen crystals: insights from density functional theory. Cryst. Growth Des..

[22] Li TL, Ayers PW, Liu SB, Swadley MJ, Aubrey-Medendorp C (2009). Crystallization force-a density functional theory concept for revealing intermolecular interactions and molecular packing in organic crystals. Chem. Eur. J..

[23] Mattei A, Li TL (2011). Interplay between molecular conformation and intermolecular interactions in conformational polymorphism: a molecular perspective from electronic calculations of Tolfenamic acid. Int J. Pharm..

[24] Zhou PP, Ayers PW, Liu SB, Li TL (2012). Natural Orbital Fukui function and application in understanding cycloaddition reaction mechanisms. Phys. Chem. Chem. Phys..

[25] Zhang MT, Li TL (2014). Intermolecular interactions in organic crystals: gaining insight from electronic structure analysis by density functional theory. Crystengcomm.

[26] Bhattacharjee R, Verma K, Zhang M, Li TL (2019). Locality and strength of intermolecular interactions in organic crystals: using Conceptual Density Functional Theory (CDFT) to characterize a highly polymorphic system. Theor. Chem. Acc..

[27] Pearson RG (1963). Hard and soft acids and bases. J. Am. Chem. Soc..

[28] Pearson RG (1966). Acids and bases. Science.

[29] Parr RG, Donnelly RA, Levy M, Palke WE (1978). Electronegativity - density functional viewpoint. J. Chem. Phys..

[30] Chattaraj PK, Lee H, Parr RG (1991). HSAB principle. J. Am. Chem. Soc..

[31] Geerlings P, De Proft F, Langenaeker W (2003). Conceptual density functional theory. Chem. Rev..

[32] Ayers PW, Liu SB, Li TL (2009). Chargephilicity and Chargephobicity: two new reactivity indicators for external potential changes from density functional reactivity theory. Chem. Phys. Lett..

[33] Liu SB, Li TL, Ayers PW (2009). Potentialphilicity and Potentialphobicity: Reactivity indicators for external potential changes from density functional reactivity theory. J. Chem. Phys..

[34] Tenenbaum JB, de Silva V, Langford JC (2000). A global geometric framework for nonlinear dimensionality reduction. Science.

[35] Law MHC, Jain AK (2006). Incremental nonlinear dimensionality reduction by manifold learning. IEEE PAMI.

[36] Lin T, Zha HB (2008). Riemannian manifold learning. IEEE PAMI.

[37] Barlow TW (1995). Self-organizing maps and molecular similarity. J. Mol. Graph.

[38] Wagener M, Sadowski J, Gasteiger J (1995). Autocorrelation of molecular surface properties for modeling corticosteroid binding globulin and cytosolic ah receptor activity by neural networks. J. Am. Chem. Soc..

[39] Klamt A (1995). Conductor-like screening model for real solvents: a new approach to the quantitative calculation of Solvation phenomena. J. Phys. Chem..

[40] Gainza P (2020). Deciphering interaction fingerprints from protein molecular surfaces using geometric deep learning. Nat. Methods.

[41] Sverrisson F., Feydy J., Correia B. E., Bronstein M. M. Fast end-to-end learning on protein surfaces. In: *IEEE/CVF Conference on Computer Vision and Pattern Recognition (CVPR)*). IEEE (2021).

[42] van der Maaten L, Hinton G (2008). Visualizing data using T-SNE. J. Mach. Learn Res.

[43] Venna J, Peltonen J, Nybo K, Aidos H, Kaski S (2010). Information retrieval perspective to nonlinear dimensionality reduction for data visualization. J. Mach. Learn. Res..

[44] Spackman MA, Jayatilaka D (2009). Hirshfeld surface analysis. Crystengcomm.

[45] Richards FM (1977). Areas, volumes, packing, and protein-structure. Annu Rev. Biophys. Bioeng..

[46] Mattei A, Li T (2014). Nucleation of conformational polymorphs: a computational study of Tolfenamic acid by explicit solvation. Cryst. Growth Des..

[47] Belongie S., Mori G., Malik J. Matching with shape contexts. *Stat Anal Shapes*, 81–105. https://link.springer.com/chapter/10.1007/0-8176-4481-4_4 (2006).

[48] Rubner Y, Tomasi C, Guibas LJ (2000). The earth mover’s distance as a metric for image retrieval. Int J. Comput Vis..

[49] Hopfinger AJ, Esposito EX, Llinas A, Glen RC, Goodman JM (2009). Findings of the challenge to predict aqueous solubility. J. Chem. Inf. Model.

[50] Llinas A, Oprisiu I, Avdeef A (2020). Findings of the second challenge to predict aqueous solubility. J. Chem. Inf. Model.

[51] Llinas A, Avdeef A (2019). Solubility challenge revisited after ten years, with multilab shake-flask data, using tight (Sd Similar to 0.17 Log) and loose (Sd Similar to 0.62 Log) test sets. J. Chem. Inf. Model.

[52] Llinas A, Glen RC, Goodman JM (2008). Solubility challenge: can you predict solubilities of 32 molecules using a database of 100 reliable measurements?. J. Chem. Inf. Model.

[53] Delaney JS (2004). ESOL: Estimating aqueous solubility directly from molecular structure. J. Chem. Inf. Comput Sci..

[54] Hinton GE, Salakhutdinov RR (2006). Reducing the dimensionality of data with neural networks. Science.

[55] Zaheer M. et al. In: *NIPS'17: Proceedings of the 31st International Conference on Neural Information Processing Systems*) (2017).

[56] Lee J. et al. Deep Neural Networks as Gaussian Processes. In: *International Conference on Learning Representations*) (2018).

[57] Williams C. K. I., Rasmussen C. E. Gaussian Processes for Regression. In: *NIPS'95*: *Proceedings of the 9th Annual Conference on Neural Information Processing Systems*) (1995).

[58] Wu Z (2018). Moleculenet: A benchmark for molecular machine learning. Chem. Sci..

[59] Fang X (2022). Geometry-enhanced molecular representation learning for property prediction. Nat. Mach. Intell..

[60] Abraham MH, Le J (1999). The correlation and prediction of the solubility of compounds in water using an amended solvation energy relationship. J. Pharm. Sci..

[61] van Tilborg D, Alenicheva A, Grisoni F (2022). Exposing the limitations of molecular machine learning with activity cliffs. J. Chem. Inf. Model.

[62] Jayatilaka D., Grimwood D. J. Tonto: A Fortran based object-oriented system for quantum chemistry and crystallography. In: *Computational Science - Iccs 2003, Pt Iv, Proceedings* (eds Sloot P. M. A., Abramson D., Bogdanov A. V., Dongarra J. J., Zomaya A. Y., Gorbachev Y. E.) (2003).

[63] Cignoni P. et al. Meshlab: An Open-Source Mesh Processing Tool. In: *Sixth Eurographics Italian Chapter Conference*) (2008).

[64] Xiao C., Hong S. & Huang W. D. Optimizing graph layout by T-SNE perplexity estimation. *Int. J. Data Sci. Anal* **15**, 159–171 (2023).

[65] Walt Svd (2014). Scikit-Image: image processing in Python. PeerJ.

[66] Vaswani A. et al. Attention Is All You Need. In: *NIPS'17: Proceedings of the 31st International Conference on Neural Information Processing Systems*) (2017).

