# Supplementary material for: Unsupervised manifold embedding to encode molecular quantum information for supervised learning of chemical data
Source: Commun Chem. 2024 Jun 11;7:133. doi: 10.1038/s42004-024-01217-z (PMC11166954; doi:10.1038/s42004-024-01217-z)
Supplement: Supplementary file 1 — Supporting Information [file 42004_2024_1217_MOESM1_ESM.pdf]

Unsupervised Manifold Embedding to Encode Molecular Quantum Information for Supervised Learning of Chemical Data

Tonglei Li,[*] Nicholas J. Huls, and Shan Lu, and Peng Hou

Department of Industrial and Physical Pharmacy, Purdue University

West Lafayette, Indiana 47907, U.S.A.

Supporting Information

Table S1. Molecules Used in the Deep Learning of Drug Solubilities to Generate Hirshfeld Surfaces and Electron-density Iso-surfaces.

Compound	*Ref Code*	*Data Source*	*LogS*	*Index*
acetaminophen	HXACAN04	2008 Set1	-1.06	0
atropine	BIHWEX	2008 Set1	-2	7
azathioprine	CIPWUT01	2008 Set1	-3.21	8
benzylimidazole	EYIRIN01	2008 Set1	-2.26	12
bromogramine	EVIDIY	2008 Set1	-4.06	14
bupivacaine	KEFFUZ	2008 Set1	-3.22	15
carprofen	UQOBIM	2008 Set1	-4.7	19
carvedilol	GIVJUQ	2008 Set1	-4.26	20
chlorpromazine	CPROMZ	2008 Set1	-5.07	22
diphenylhydantoin	PHYDAN03	2008 Set1	-3.86	43
enrofloxacin	XICPAC	2008 Set1	-3.18	45
famotidine	FOGVIG01	2008 Set1	-2.65	50
flufenamic acid	FPAMCA11	2008 Set1	-5.35	52
guanine	KEMDOW	2008 Set1	-4.43	58
hexobarbital	DMCYBA	2008 Set1	-2.67	60
hydroflumethiazide	EWUHAF	2008 Set1	-2.97	62
hydroxybenzoic acid	JOZZIH01	2008 Set1	-1.46	63
ibuprofen	IBPRAC	2008 Set1	-3.59	64
mefenamic acid	XYANAC	2008 Set1	-6.74	70
metoclopramide	METPRA	2008 Set1	-3.57	71
metronidazole	MNIMET	2008 Set1	-1.22	72
nalidixic acid	NALIDX	2008 Set1	-3.61	76
naphthol	NAPHOL01	2008 Set1	-1.98	78
niflumic acid	NIFLUM11	2008 Set1	-4.58	82
nitrofurantoin	LABJON	2008 Set1	-3.24	84
oxytetracycline	OXYTET	2008 Set1	-3.09	89
phenobarbital	PHBARB	2008 Set1	-2.29	93
phenylbutazone	JAJLIQ	2008 Set1	-4.39	94
phthalic acid	PHTHAC06	2008 Set1	-1.61	95
piroxicam	BIYSEH	2008 Set1	-4.8	97
propranolol	PROPRA10	2008 Set1	-3.49	102
ranitidine	POPFAC	2008 Set1	-2.5	105
sparfloxacin	JEKMOB	2008 Set1	-3.37	111
sulfacetamide	SLFNMG01	2008 Set1	-1.52	114
sulfamethazine	SLFNMD11	2008 Set1	-2.73	117
thymol	IPMEPL	2008 Set1	-2.19	128
trichloromethiazide	KIKCUD	2008 Set1	-3.53	130
trimethoprim	AMXBPM12	2008 Set1	-2.95	131
tryptamine	XUDTOF	2008 Set1	-3.3	132
benzocaine	QQQAXG02	2008 Set2	-2.33	10
clozapine	NDNHCL10	2008 Set2	-3.24	30
dibucaine	WADTIG	2008 Set2	-4.39	37
diethylstilbestrol	ESTILO02	2008 Set2	-4.43	39

furosemide	FURSEM14	2008 Set2	-4.23	54
hydrochlorothiazide	HCSBTZ	2008 Set2	-2.68	61
ketoprofen	KEMRUP	2008 Set2	-3.21	68
meclofenamic acid	MECLOF12	2008 Set2	-6.27	69
naphthoic acid	NAPHAC	2008 Set2	-3.77	77
pyrimethamine	MUFMAB01	2008 Set2	-4.11	103
salicylic acid	SALIAC	2008 Set2	-1.93	110
sulfamerazine	SLFNMA01	2008 Set2	-3.12	116
sulfamethizole	AMUTOT	2008 Set2	-2.78	118
tolbutamide	ZZZPUS02	2008 Set2	-3.46	129
acetazolamide	ATDZSA	2019 Set1	-2.38	1
acetylsalicylic acid	ACSALA	2019 Set1	-1.67	2
alclofenac	FICJAC	2019 Set1	-4.4	3
ambroxol	FIYBEV01	2019 Set1	-3.87	4
aripiprazole	MELFIT01	2019 Set1	-6.64	5
atovaquone	UMOMAL02	2019 Set1	-6.07	6
baclofen	AQEKUE	2019 Set1	-1.78	9
benzthiazide	AMEVAP	2019 Set1	-4.84	11
bromazepam	CAGWOW	2019 Set1	-3.39	13
carbamazepine	CBMZPN10	2019 Set1	-3.22	16
carbazole	CRBZOL01	2019 Set1	-5.19	17
carbendazim	SEDZUW01	2019 Set1	-4.56	18
celecoxib	DIBBUL	2019 Set1	-5.89	21
chlorpropamide	BEDMIG	2019 Set1	-3.17	23
cholic acid	EDEBOE	2019 Set1	-4.62	25
cilostazol	XOSGUH	2019 Set1	-4.93	26
cimetidine	CIMETD	2019 Set1	-1.52	27
ciprofloxacin	UHITOV	2019 Set1	-3.57	28
corticosterone	CORTIC	2019 Set1	-3.29	31
cortisoneacetate	ACPRET	2019 Set1	-4.22	32
daidzein	XEKCUO	2019 Set1	-5.23	34
diazoxide	DIAZOX	2019 Set1	-3.43	36
diclofenac	SIKLIH	2019 Set1	-5.34	38
diflorasone diacetate	IHAPOX	2019 Set1	-4.82	40
diltiazem	GAJKAG	2019 Set1	-3.02	42
DOPA	LDOPAS11	2019 Set1	-1.76	44
estradiol	HXESDO	2019 Set1	-5	46
estrone	ESTRON10	2019 Set1	-5.38	47
eucalyptol	MOFPAY	2019 Set1	-1.66	48
flurbiprofen	FLUBIP	2019 Set1	-4.34	53
ganciclovir	UGIVAI	2019 Set1	-1.78	55
glipizide	SAXFED	2019 Set1	-5.61	56
griseofulvin	GRISFL	2019 Set1	-4.52	57
haloperidol	HALDOL01	2019 Set1	-5.71	59
indomethacin	INDMET	2019 Set1	-5.48	65
indoprofen	LEKMET	2019 Set1	-4.65	66

ketoconazole	KCONAZ	2019 Set1	-5.47	67
nabumetone	XOCXUI02	2019 Set1	-4.4	75
naproxen	COYRUD	2019 Set1	-4.23	79
nevirapine	PABHIJ	2019 Set1	-3.41	80
nifedipine	BICCIZ	2019 Set1	-4.71	81
nimesulide	WINWUL	2019 Set1	-4.74	83
norfloxacin	VETVOG	2019 Set1	-2.88	85
noscapine	BOVPUA	2019 Set1	-4.48	86
ofloxacin	CUYCEF	2019 Set1	-2.03	87
papaverine	MVERIQ01	2019 Set1	-4.33	90
perphenazine	PERPAZ	2019 Set1	-4.48	91
phenacetin	PYRAZB21	2019 Set1	-2.3	92
pindolol	PINDOL	2019 Set1	-3.75	96
prednisolone, methyl-	MTHPRG	2019 Set1	-3.33	98
primidone	EPHPMO	2019 Set1	-2.53	99
probenecid	QQQBSS03	2019 Set1	-4.83	100
repaglinide	JOHKUM	2019 Set1	-4.77	106
resveratrol	DALGON	2019 Set1	-3.75	107
ritonavir	YIGPIO03	2019 Set1	-5.17	108
rofecoxib	CAXMUJ	2019 Set1	-4.61	109
spironolactone	ATPRCL10	2019 Set1	-4.21	112
strychnine	ZZZUEE01	2019 Set1	-3.38	113
sulfasalazine	QIJZOY	2019 Set1	-6.41	119
sulfathiazole	SUTHAZ	2019 Set1	-2.62	120
sulfisomidine	SLFSMD	2019 Set1	-2.16	121
sulfisoxazole	SLFSXZ10	2019 Set1	-3.13	122
sulindac	DOHREX	2019 Set1	-4.96	123
thiacetazone	GANGEH	2019 Set1	-3.5	127
chlorprothixene	CMAPTX	2019 Set2	-5.99	24
clofazimine	DAKXUI	2019 Set2	-9.05	29
curcumin	BINMEQ02	2019 Set2	-5.36	33
danazol	YAPZEU01	2019 Set2	-6.1	35
diflunisal	FAFWIS01	2019 Set2	-4.99	41
ezetimibe	QUWYIR	2019 Set2	-4.94	49
fentiazac	PCPTZA	2019 Set2	-5.84	51
miconazole	PAVPIP	2019 Set2	-5.82	73
mifepristone	ZIDLED	2019 Set2	-5.22	74
omeprazole	VAYXOI	2019 Set2	-3.7	88
procaine	BEWYIL01	2019 Set2	-2.3	101
quinine	BOMDUC	2019 Set2	-3.06	104
sulfadimethoxine	SFDMOX	2019 Set2	-3.74	115
telmisartan	XUYHOO	2019 Set2	-6.73	124
terfenadine	EWEMIF	2019 Set2	-7.74	125
thiabendazole	THBDAZ10	2019 Set2	-3.97	126

Table S2. MEMS of Molecules Used in the Solubility Prediction that are Derived from Hirshfeld Surfaces.

Compound	*MEMS (ESP and F^2 of close and 4 cuts)*
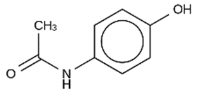 acetaminophen	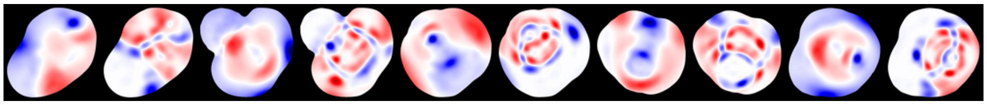
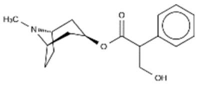 atropine	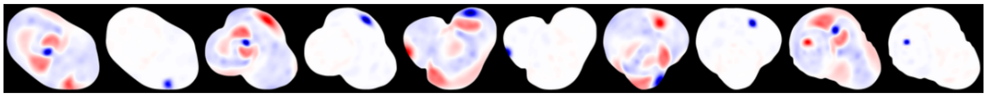
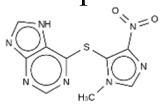 azathioprine	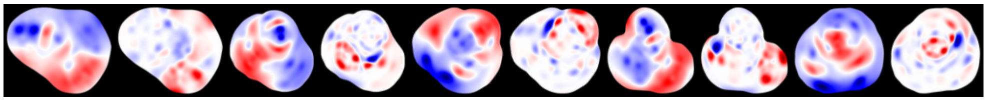
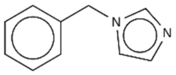 benzylimidazole	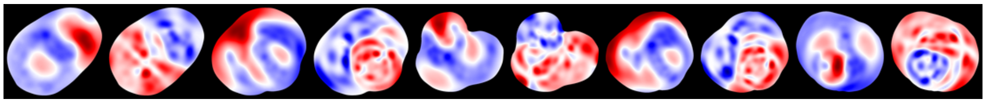
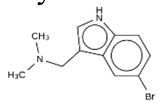 bromogramine	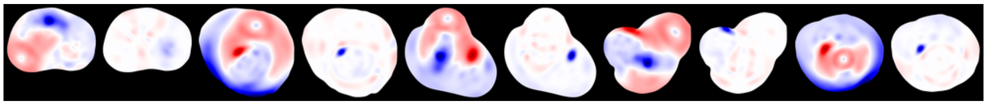
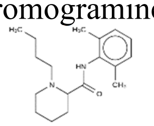 bupivacaine	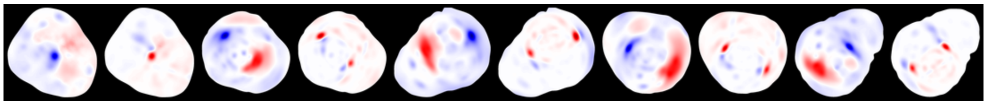
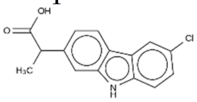 carprofen	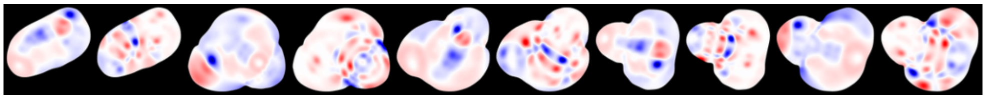
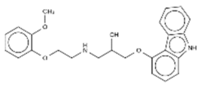 carvedilol	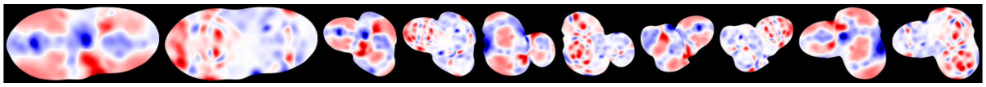
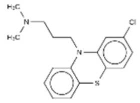 chlorpromazine	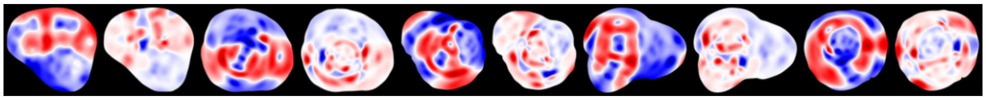
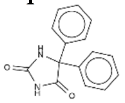 diphenylhydantoin	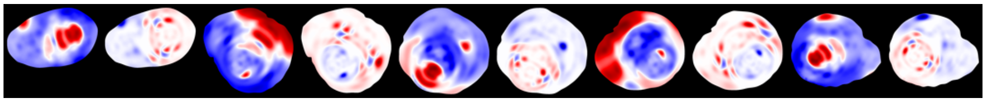
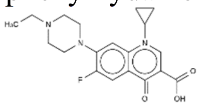 enrofloxacin	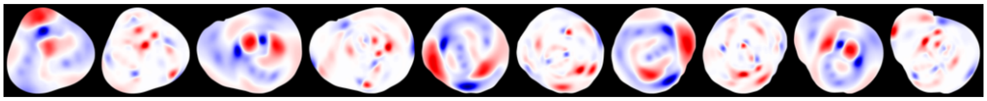

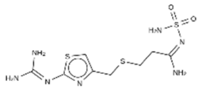
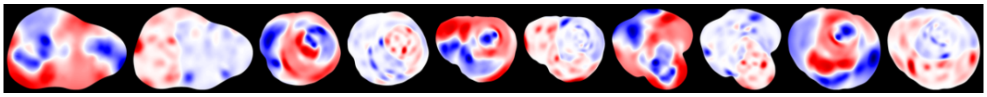

famotidine

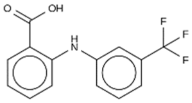
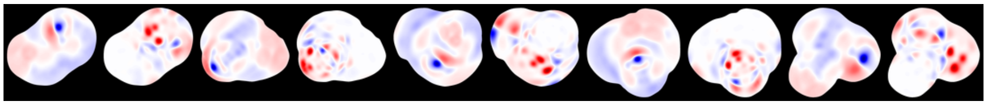

flufenamic acid

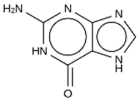
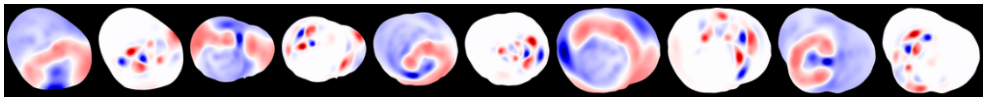

guanine

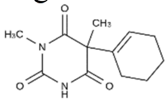
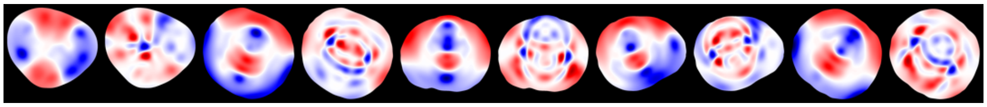

hexobarbital

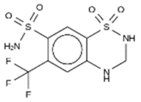
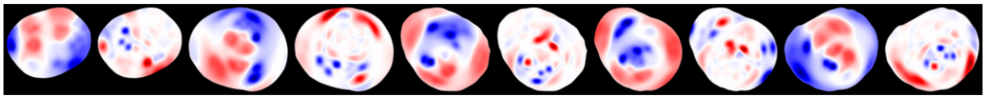

hydroflumethiazide

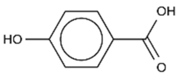
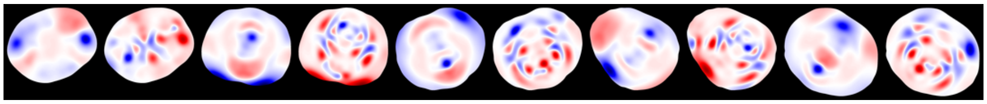

hydroxybenzoic acid

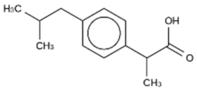
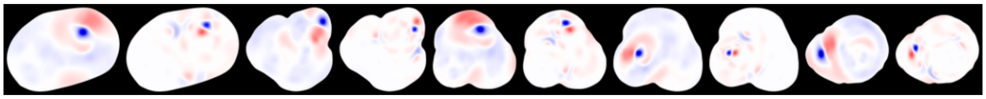

ibuprofen

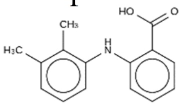
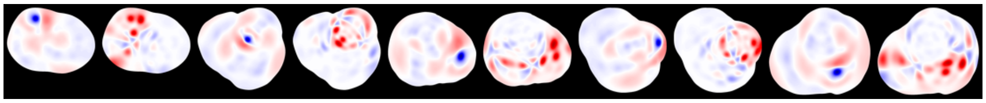

mefenamic acid

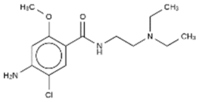
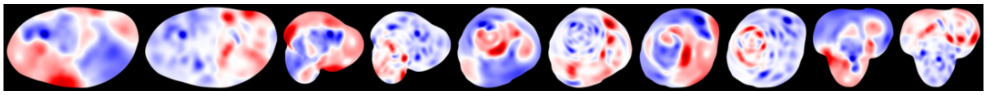

metoclopramide

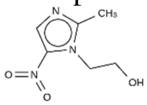
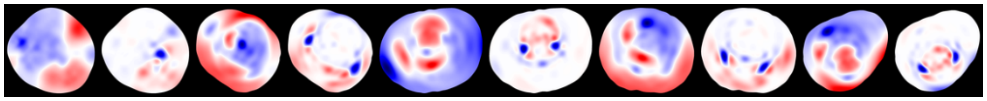

metronidazole

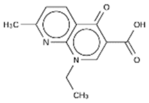
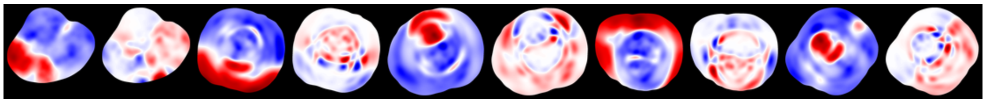

nalidixic acid

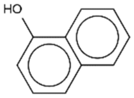
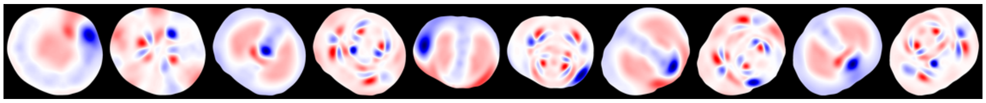

naphthol

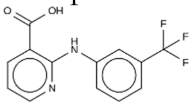
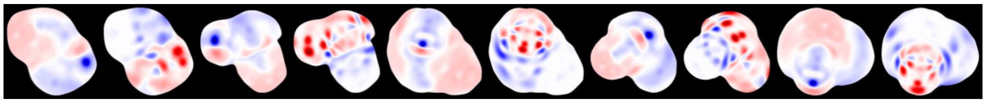

niflumic acid

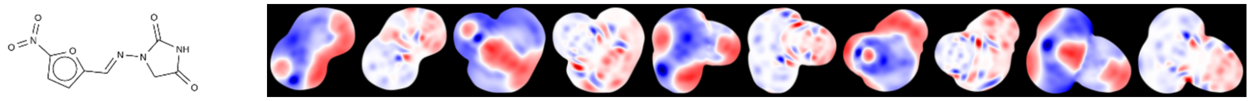
nitrofurantoin

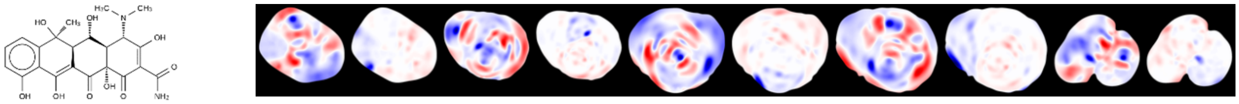
oxytetracycline

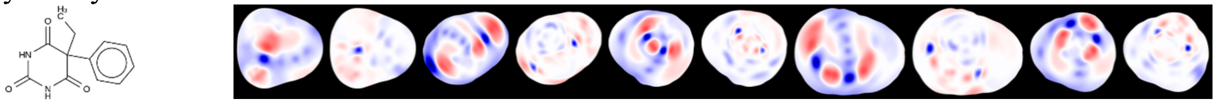
phenobarbital

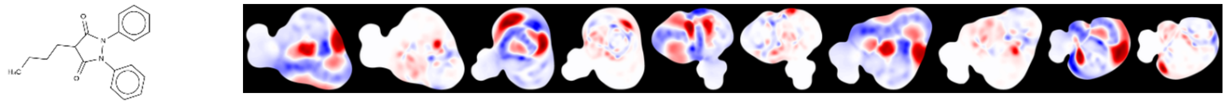
phenylbutazone

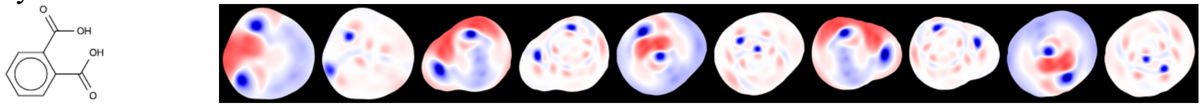
phthalic acid

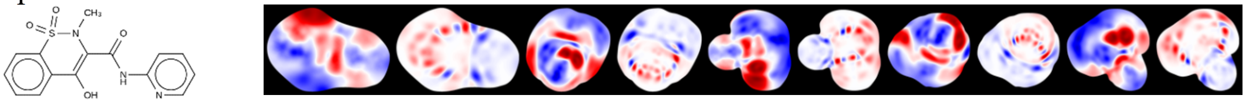
piroxicam

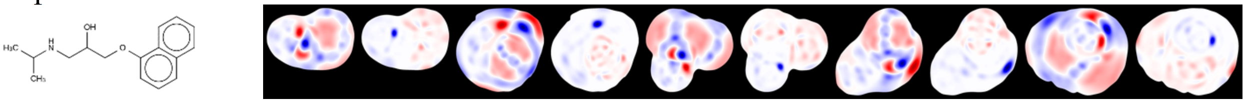
propranolol

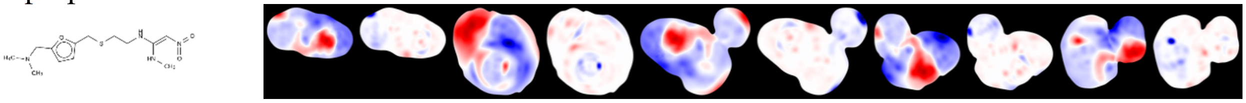
ranitidine

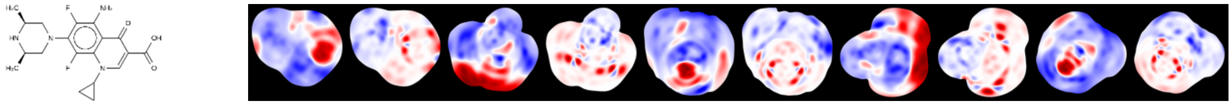
sparfloxacin

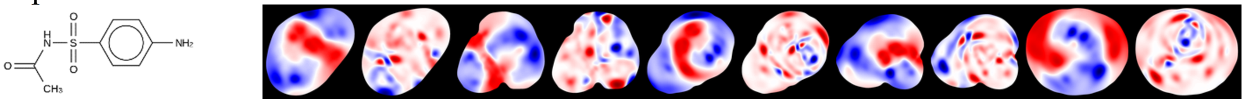
sulfacetamide

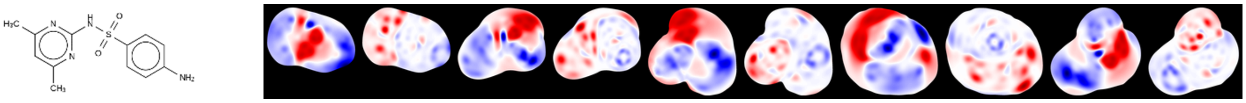
sulfamethazine

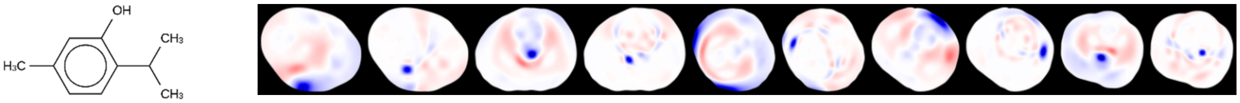
thymol

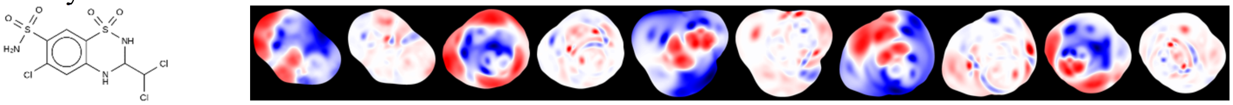
trichloromethiazide

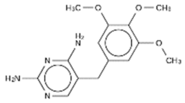
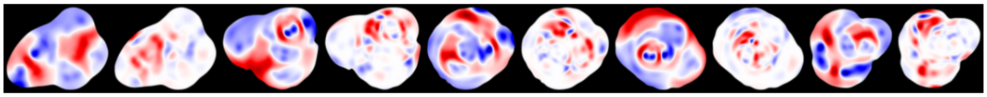

trimethoprim

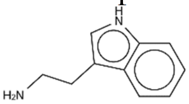
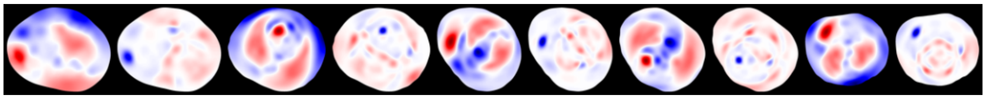

tryptamine

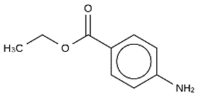
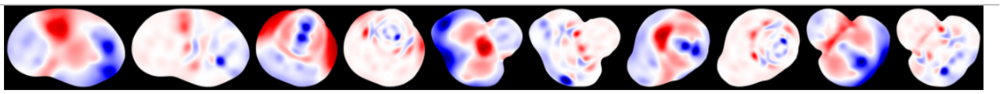

benzocaine

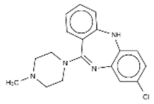
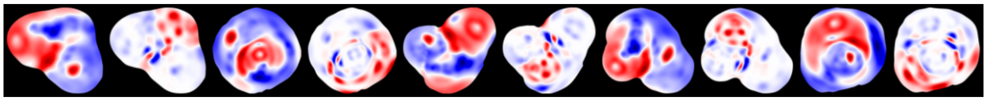

clozapine

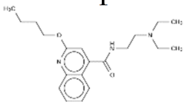
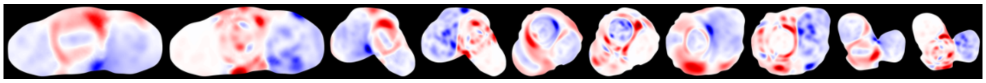

dibucaine

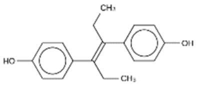
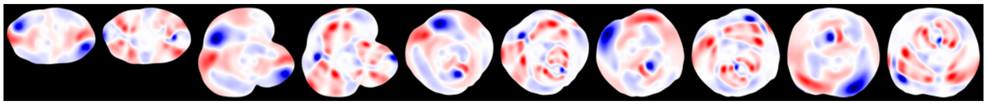

diethylstilbestrol

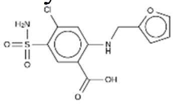
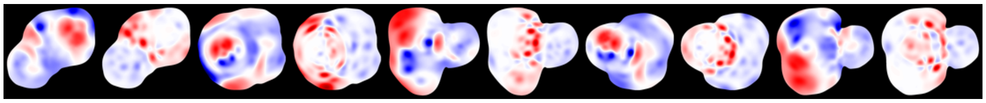

furosemide

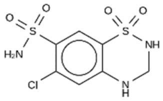
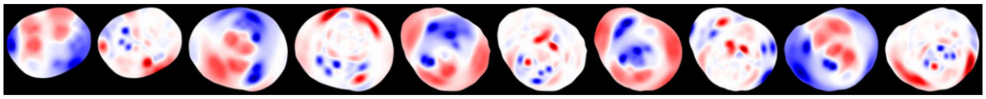

hydrochlorothiazide

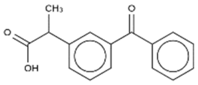
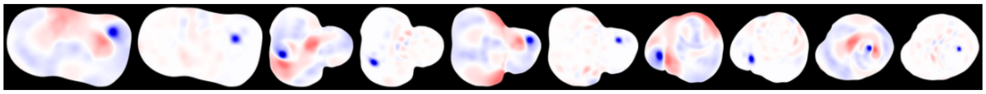

ketoprofen

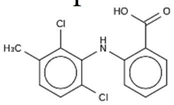
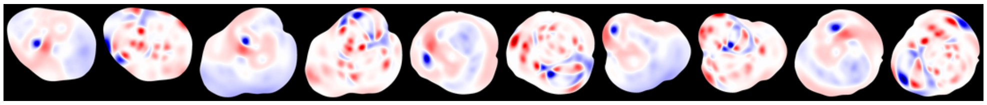

meclofenamic acid

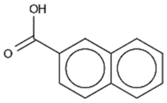
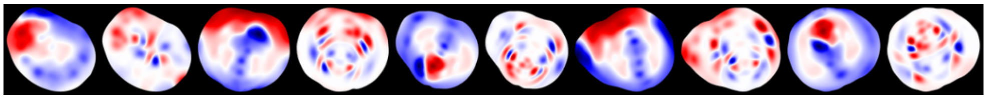

naphthoic acid

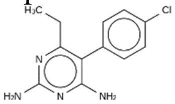
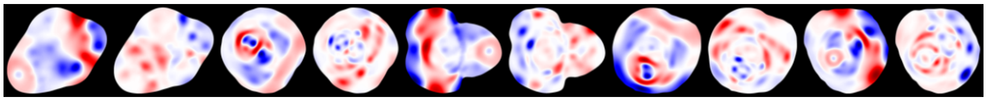

pyrimethamine

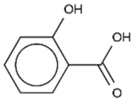
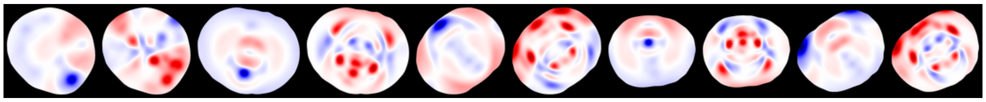

salicylic acid

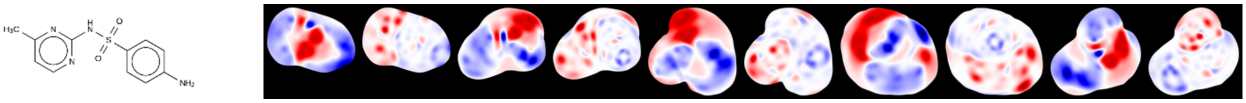

sulfamerazine

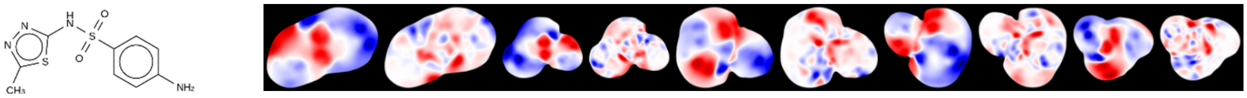

sulfamethizole

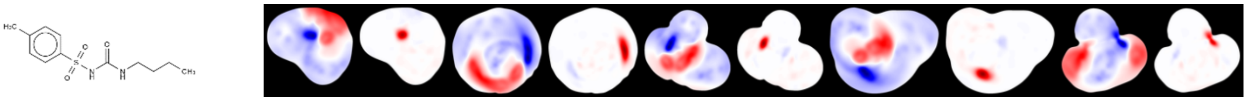

tolbutamide

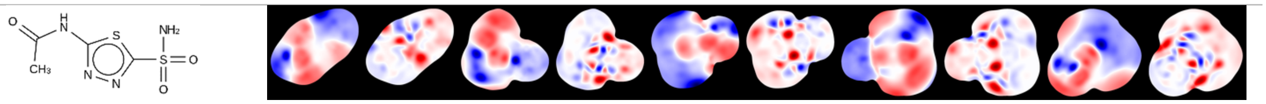

acetazolamide

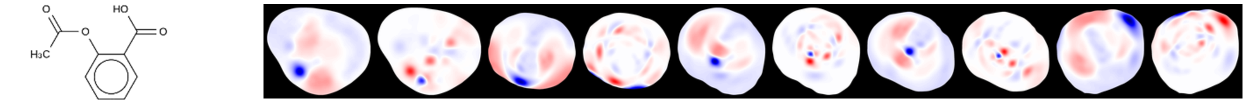

acetylsalicylic acid

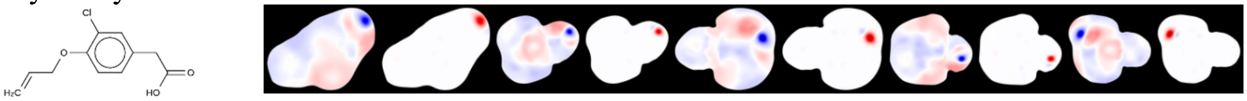

alclofenac

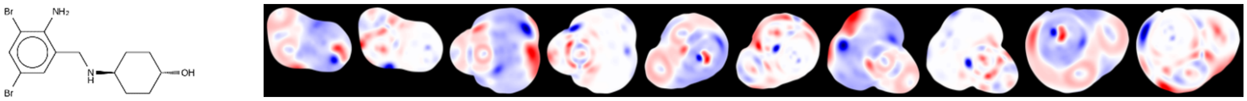

ambroxol

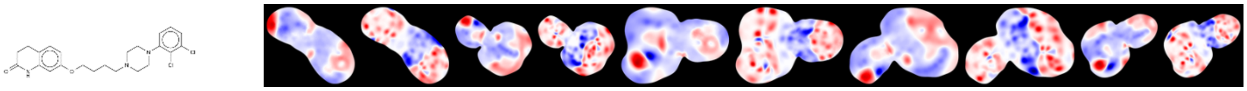

aripiprazole

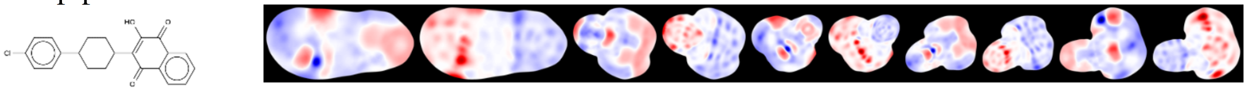

atovaquone

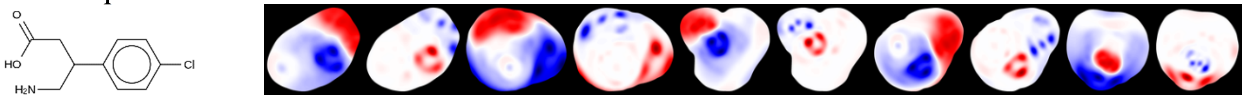

baclofen

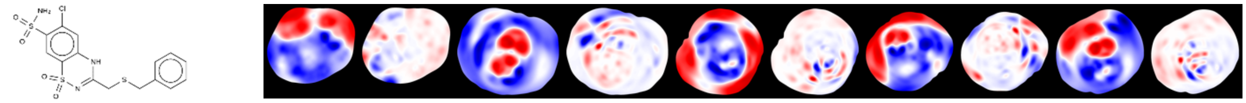

benzthiazide

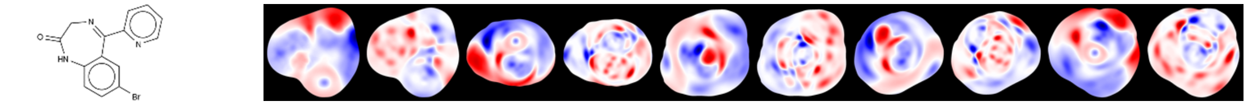

bromazepam

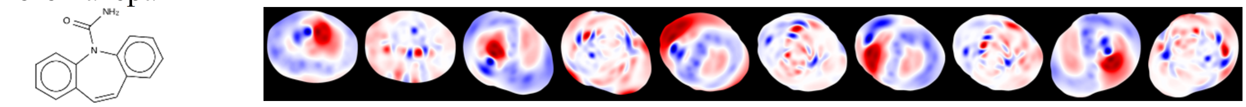

carbamazepine

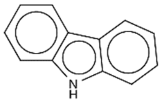
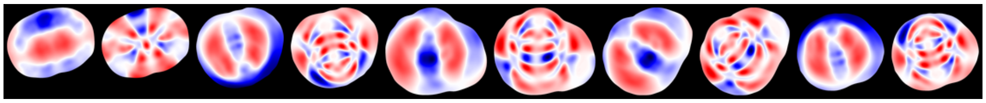

carbazole

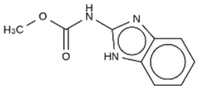
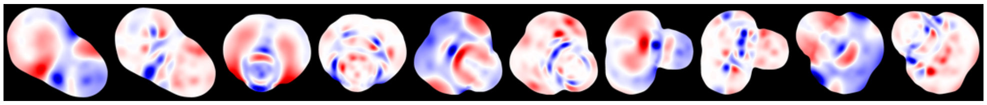

carbendazim

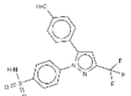
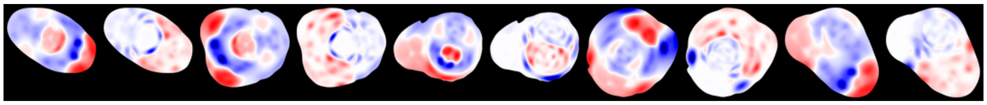

celecoxib

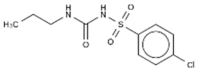
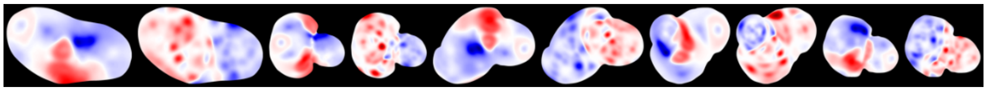

chlorpropamide

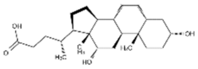
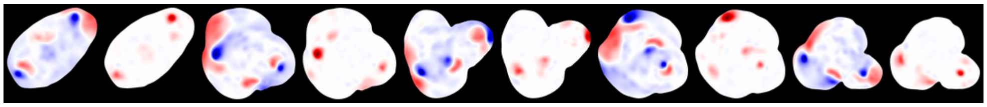

cholic acid

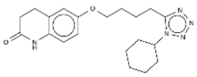
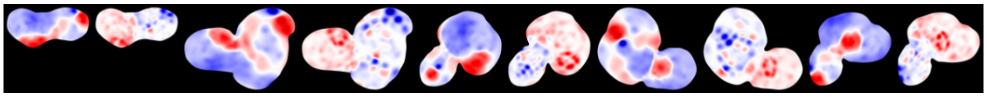

cilostazol

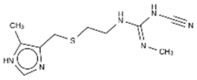
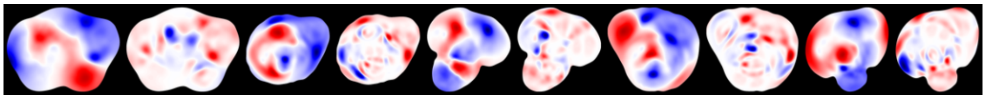

cimetidine

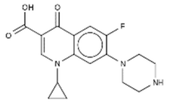
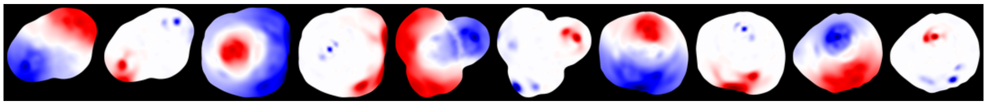

ciprofloxacin

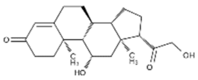
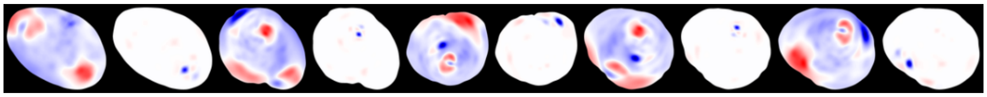

corticosterone

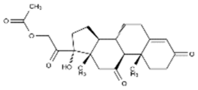
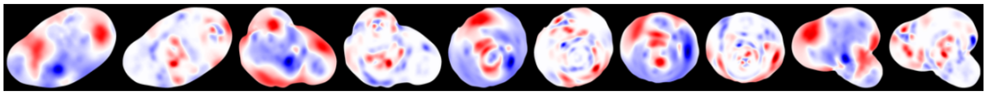

cortisone acetate

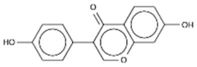
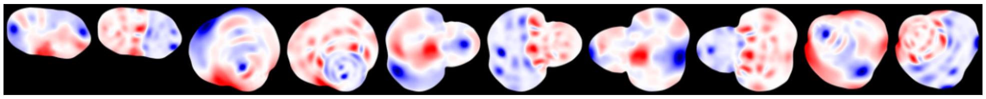

daidzein

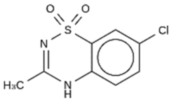
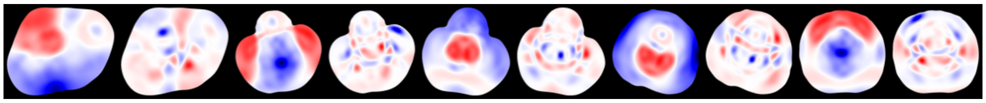

diazoxide

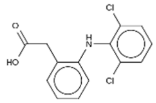
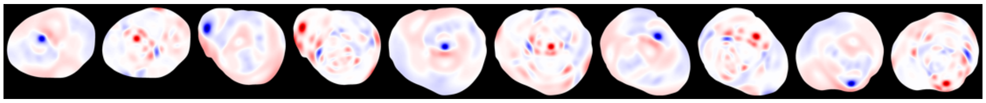

diclofenac

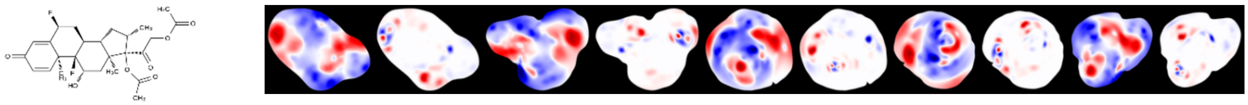

diflorasone diacetate

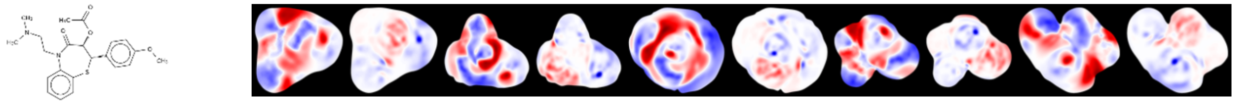

diltiazem

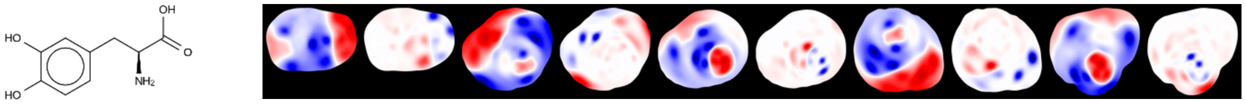

DOPA

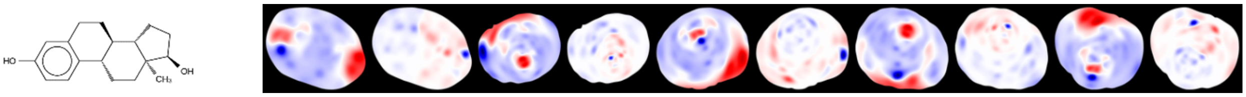

estradiol

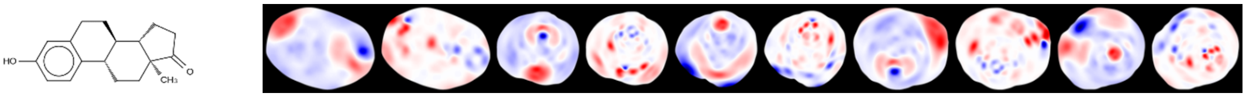

estrone

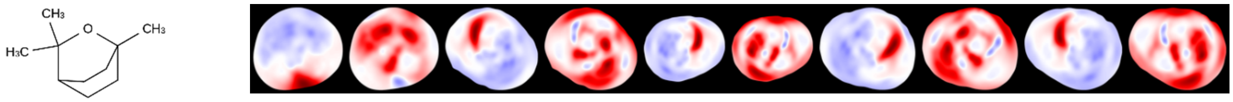

eucalyptol

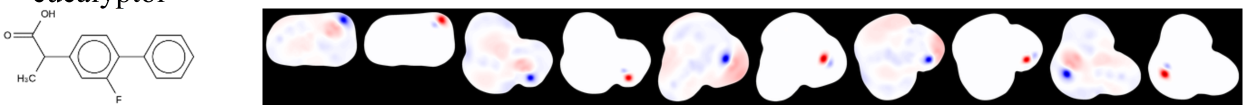

flurbiprofen

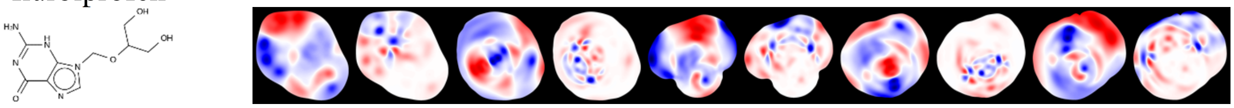

ganciclovir

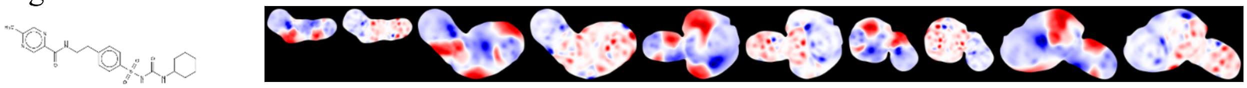

glipizide

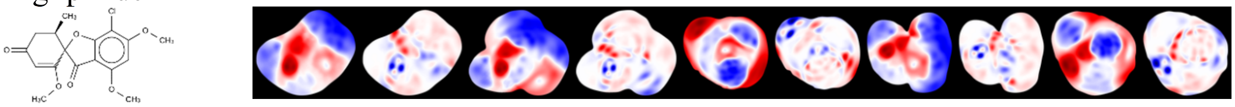

griseofulvin

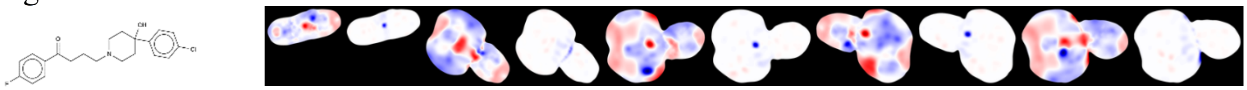

haloperidol

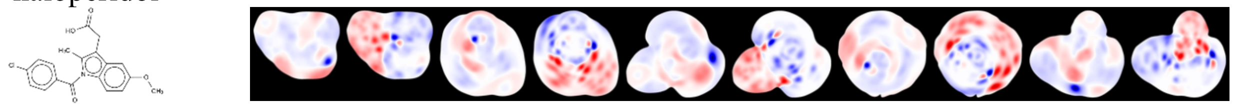

indomethacin

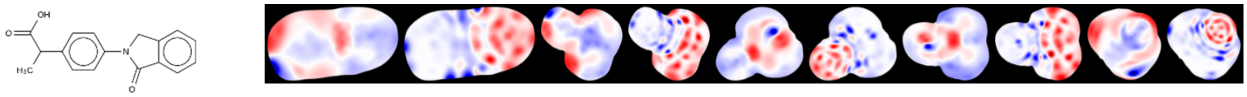

indoprofen

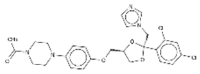

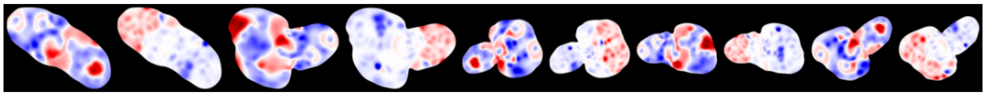

ketoconazole

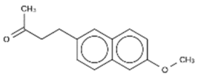

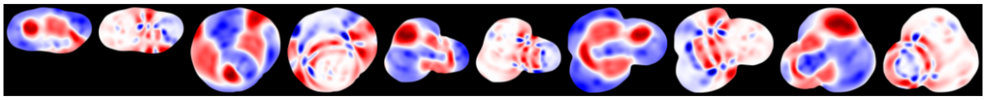

nabumetone

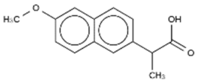

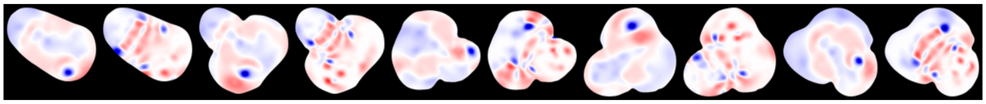

naproxen

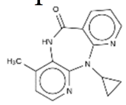

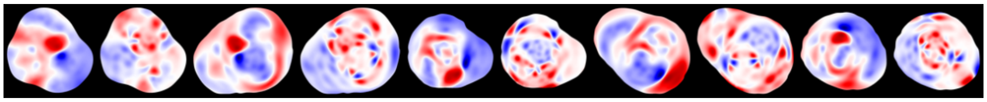

nevirapine

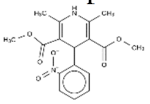

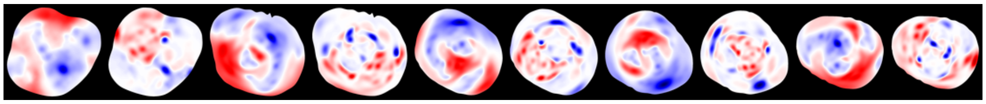

nifedipine

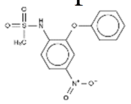

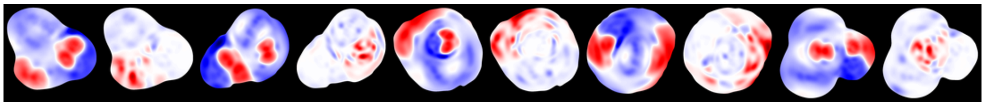

nimesulide

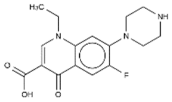

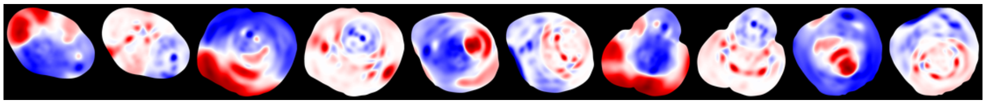

norfloxacin

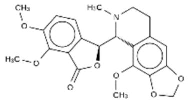

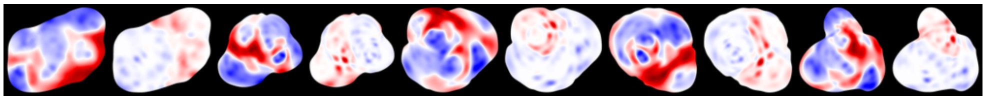

noscapine

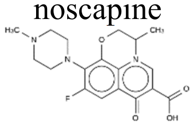

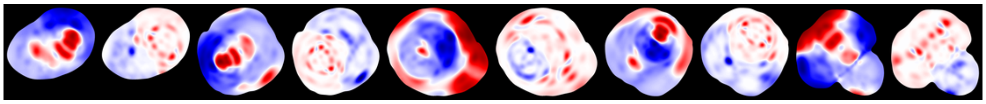

ofloxacin

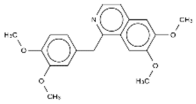

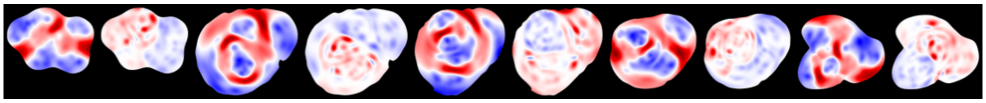

papaverine

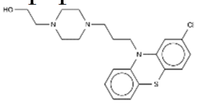

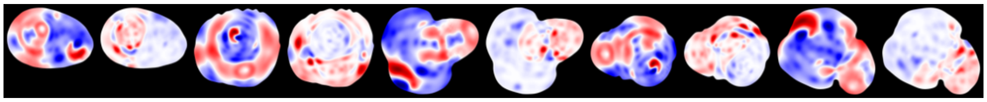

perphenazine

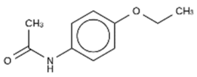

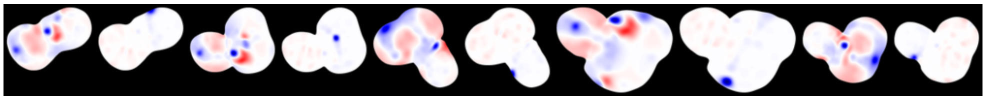

phenacetin

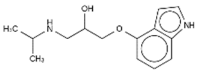

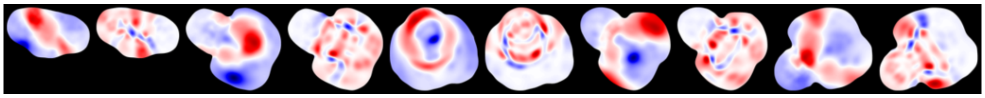

pindolol

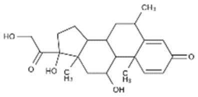

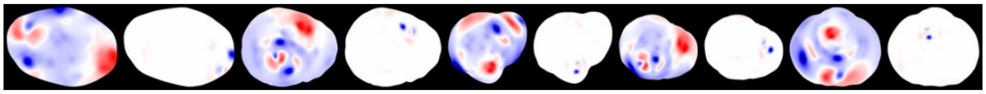

prednisolone, methyl-

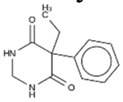

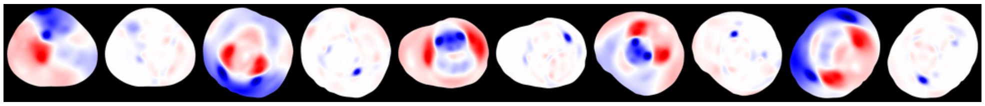

primidone

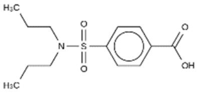

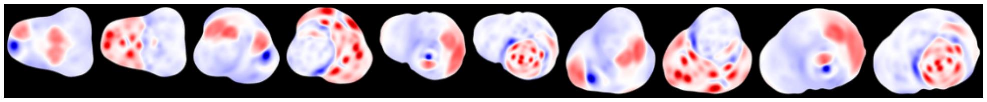

probenecid

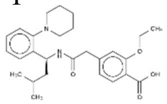

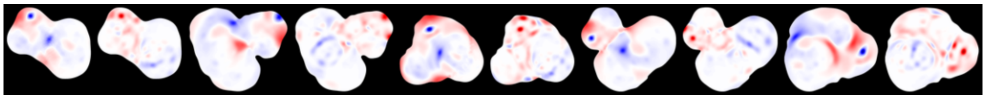

repaglinide

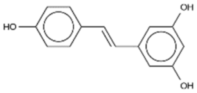

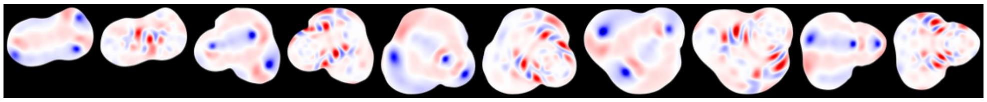

resveratrol

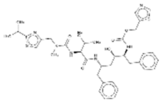

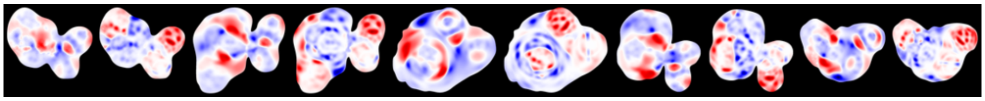

ritonavir

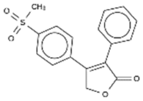

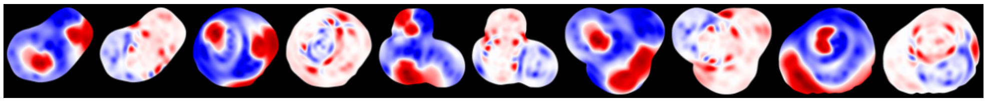

rofecoxib

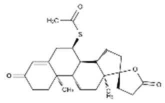

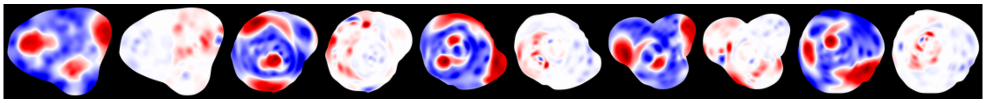

spironolactone

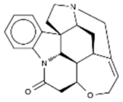

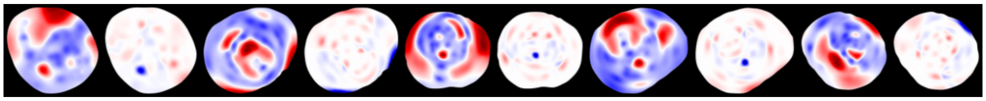

strychnine

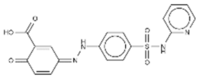

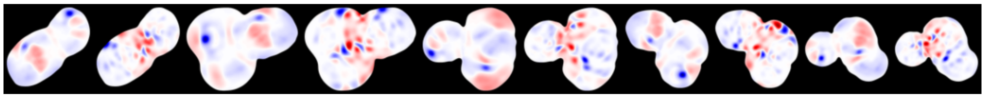

sulfasalazine

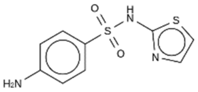

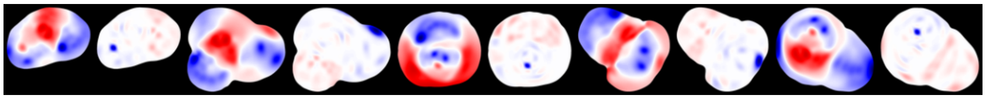

sulfathiazole

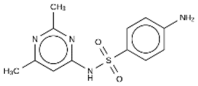

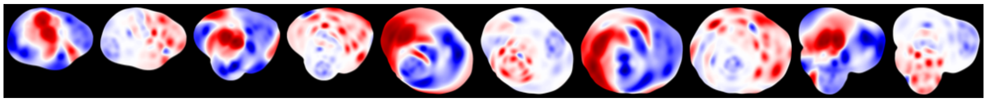

sulfisomidine

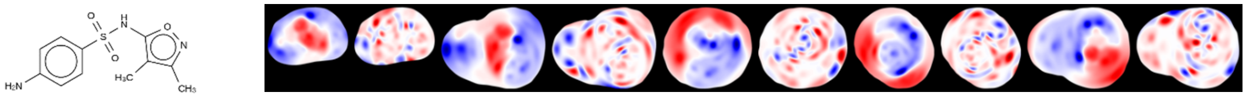

sulfisoxazole

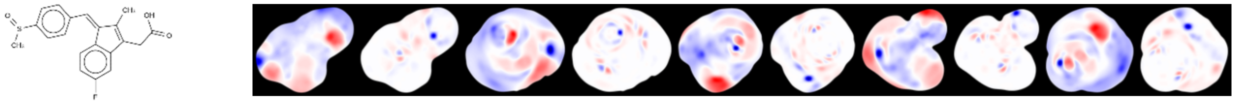

sulindac

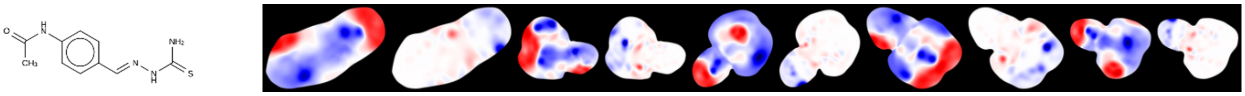

thiacetazone

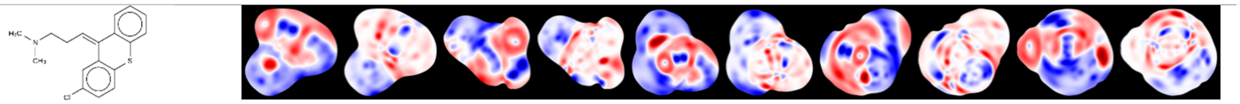

chlorprothixene

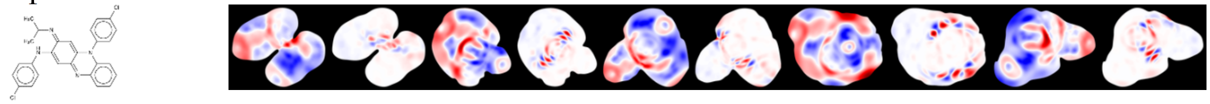

clofazimine

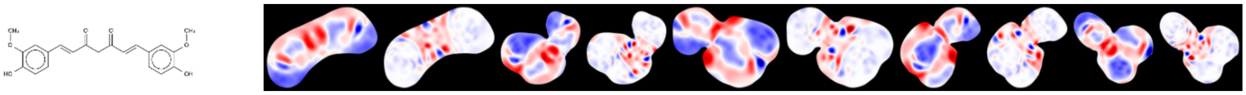

curcumin

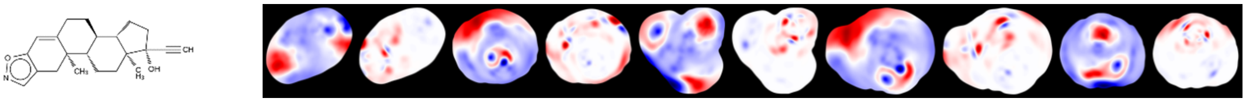

danazol

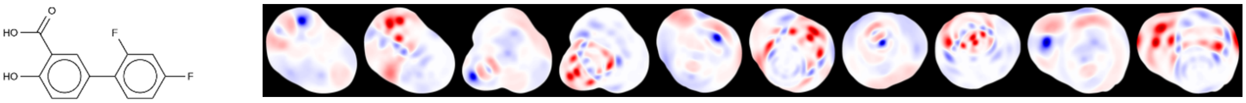

diflunisal

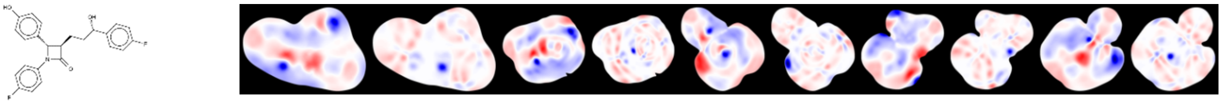

ezetimibe

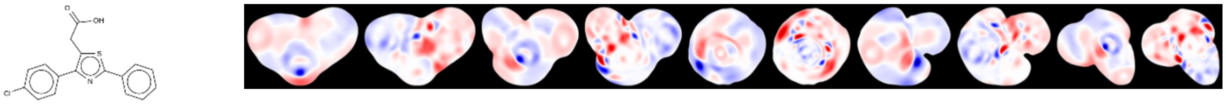

fentiazac

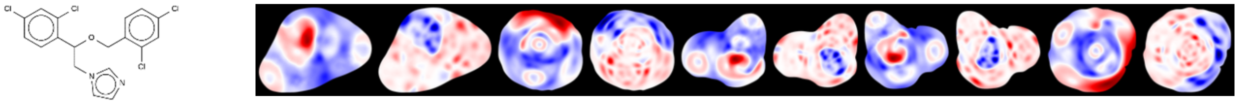

miconazole

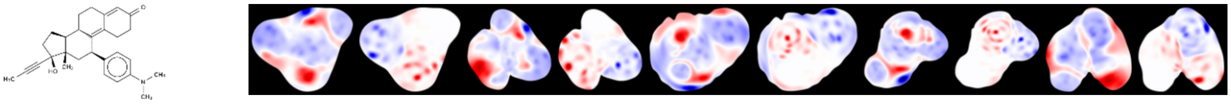

mifepristone

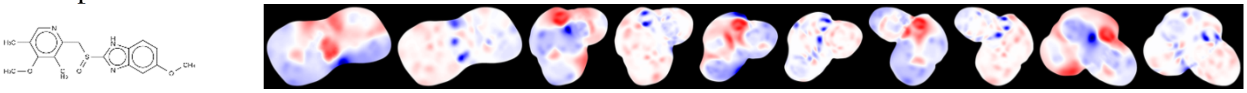

omeprazole

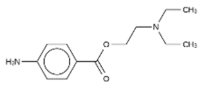 procaine	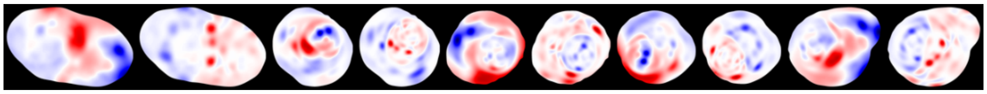
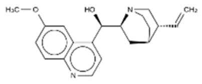 quinine	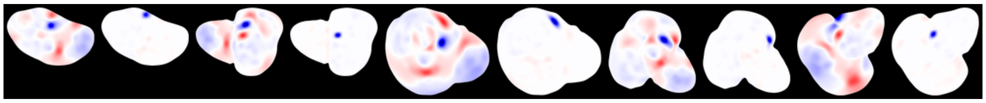
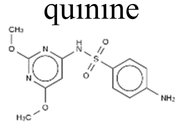 sulfadimethoxine	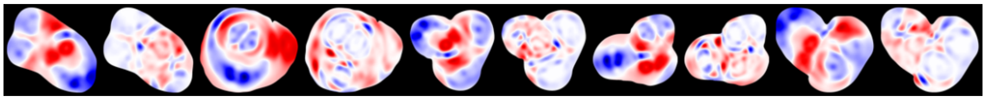
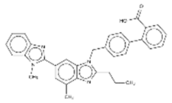 telmisartan	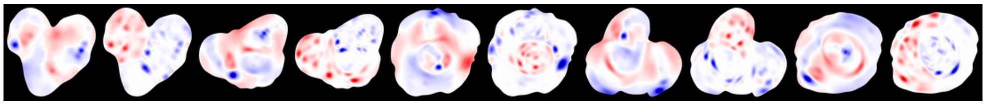
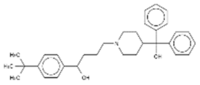 terfenadine	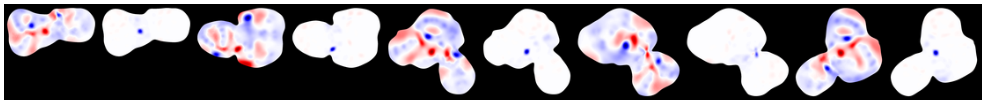
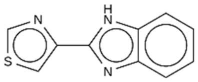 thiabendazole	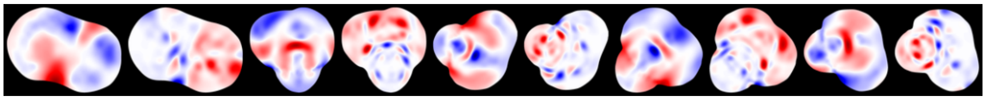

Table S3. Prediction Metrics by Top Performers Reported in the 2nd Solubility Challenge.[50]

Participant	*RMSE (Set 1 & 2)[a]*	*R^2 (Set 1 & 2)*	*%±0.5log (Set 1 & 2)*	*Method [b]*	*Molecular Representation*
PMSA_B	0.86; 1.13	0.54; 0.72	39; 38	RBF	StarDrop molecular descriptors (N=167)
PMSA_C	0.78; 1.27	0.62; 0.65	54; 41	RBF	StarDrop molecular descriptors (N=167)
MLKC_C	0.80; 1.33	0.60; 0.61	44; 44	lightGBM	Dragon 6.0 all descriptors; RDKit fingerprints
JHUNC_A	0.83; 1.79	0.57; 0.30	61; 28	ANN	Concatenated Morgan, hashed atom pair, hashed topological torsion fingerprints with MACCS keys
SGURV	1.07; 1.16	0.28; 0.71	56; 34	ANN	Mordred molecular descriptors
JCSU_A	0.92; 1.39	0.48; 0.58	39; 38	RFR	MOE 2D and 3D descriptors (N=269)
UMUT_B	0.94; 1.32	0.45; 0.62	42; 34	MLR	RDKit descriptors (MolLogP, TPSA)
UMUT_C	0.99; 1.06	0.38; 0.75	42; 44	MLR	XLogS; PaDEL descriptors (SpMax1_Bhp, SHBd)
This work [c]	0.53; 0.67	0.82; 0.77	69.5; 56.7	ANN	MEMS

[a] The two datasets in the 2nd Solubility Challenge have different standard deviations of logS due to inter-laboratory measurements, 0.17 (Set 1; 100 molecules) and 0.62 (Set 2; 32 molecules).

[b] The methods are RBF (radial basis function), MLR (multiple linear regression), ANN (artificial neural network), RFR (random forest regression), and light GBM (light gradient boosting model).

[c] The two sets of metrics are of the shallow learning of 133 (partially optimized) and 200 (fully optimized) molecules by 4 cut ISO MEMS at 95:5 split of the datasets, respectively. See Table 2.

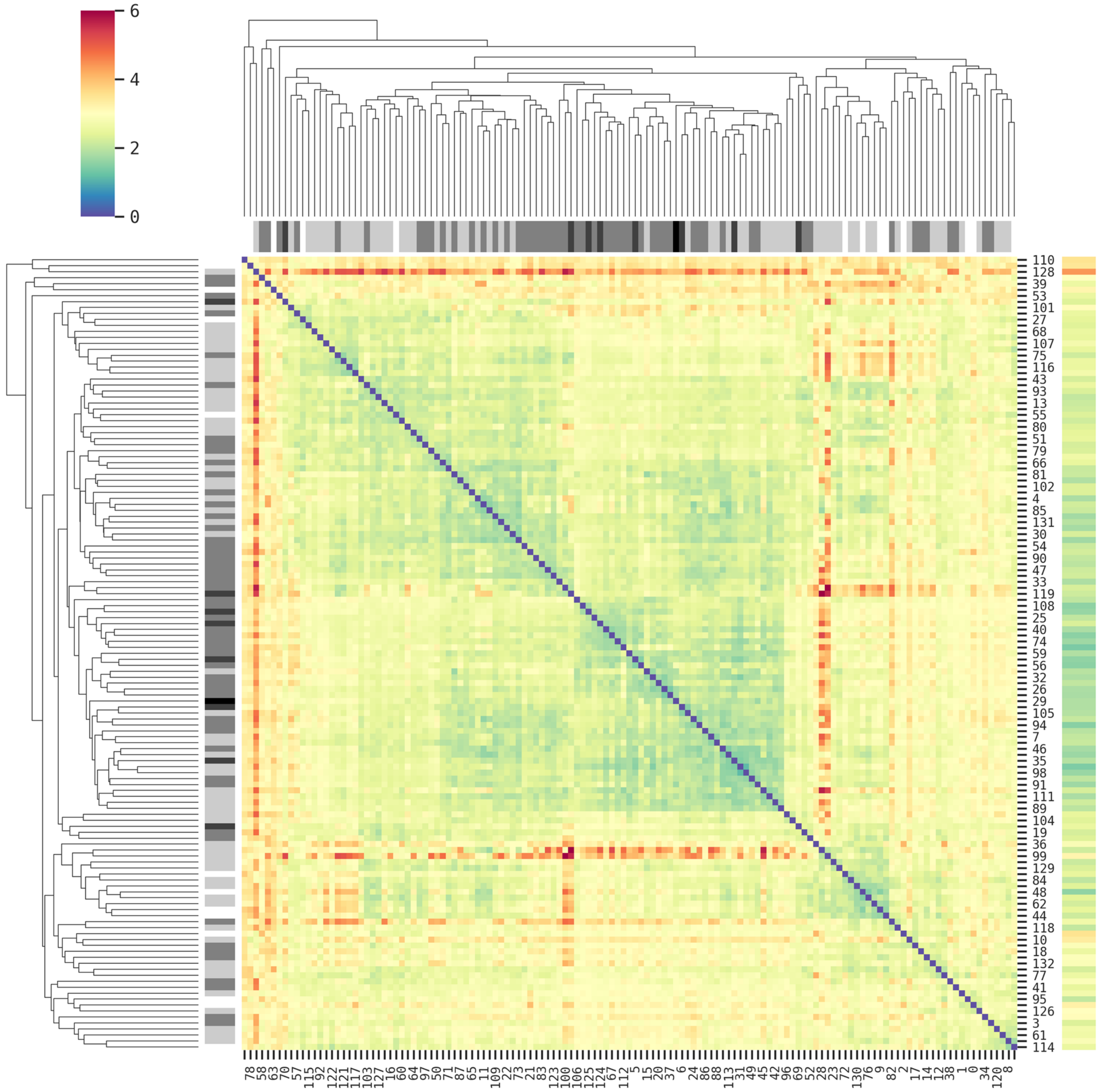

Figure S1. Heatmap and cluster analysis of EMD values between shape-context matrices of the positive electrostatic potentials on *closed* MEMS calculated of Hirshfeld surfaces of 133 molecules utilized in the deep-learning prediction of water solubility. Evenly indexed molecules are marked on the right and oddly indexed ones are marked on the bottom. The indices can be found in Table S1. The gray bar under each dendrogram is of solubility with white, light gray, mid-gray, dark gray, and black marking LogS > -2.0, > -4.0 and <= -2.0, > -6.0 and <= -4.0, >-8.0 and <= -6.0, and <-8.0, respectively. The color bar on the far right is of the averaged EMD of each molecule among close and its four cut MEMS, corresponding to the respective molecules by the rows.

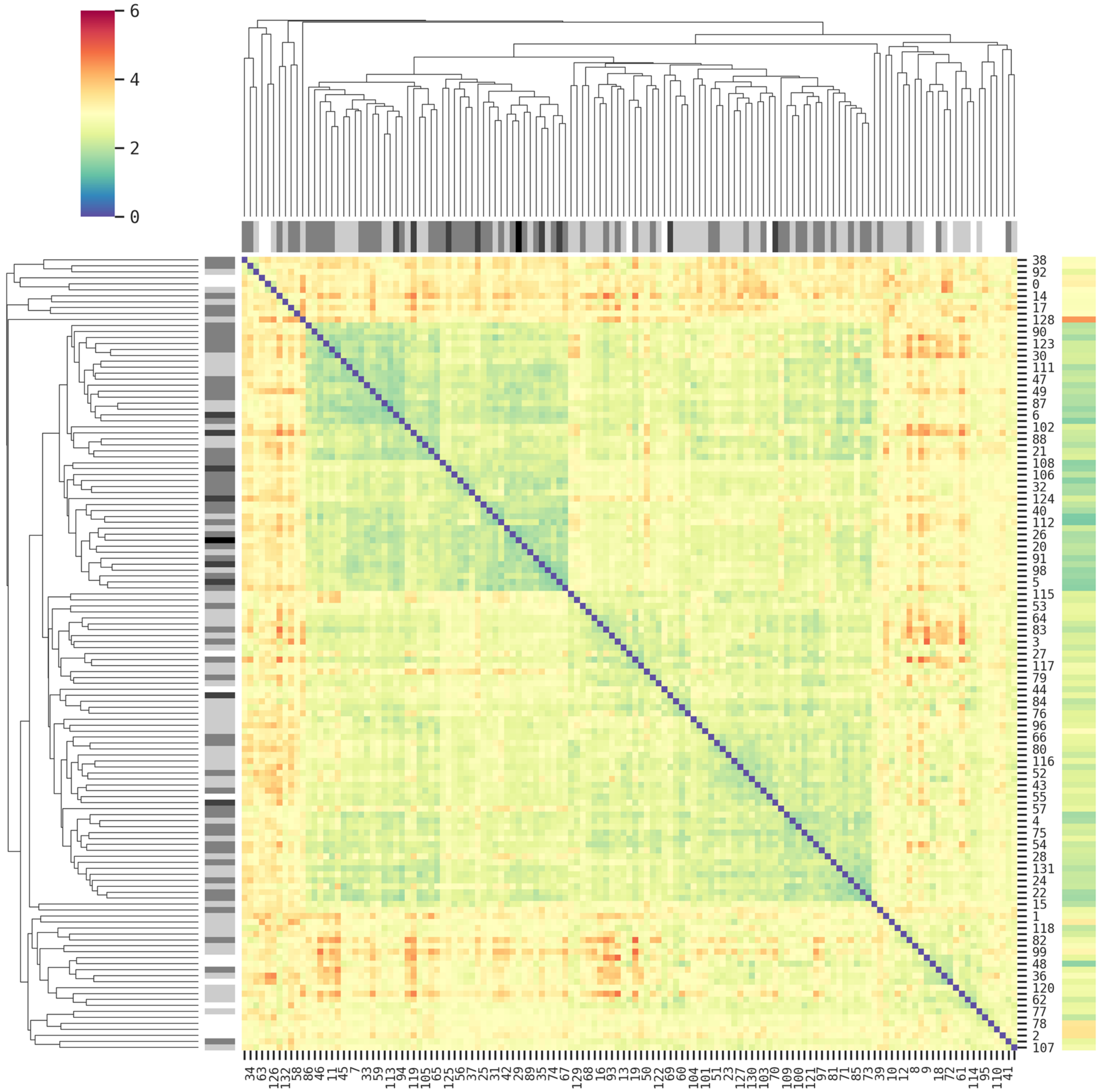

Figure S2. Heatmap and cluster analysis of EMD values between shape-context matrices of the positive electrostatic potentials on *cut* MEMS calculated of Hirshfeld surfaces of 133 molecules utilized in the deep-learning prediction of water solubility. Evenly indexed molecules are marked on the right and oddly indexed ones are marked on the bottom. The indices can be found in Table S1. The gray bar under each dendrogram is of solubility with white, light gray, mid-gray, dark gray, and black marking LogS > -2.0, > -4.0 and <= -2.0, > -6.0 and <= -4.0, >-8.0 and <= -6.0, and <-8.0, respectively. The color bar on the far right is of the averaged EMD of each molecule among close and its four cut MEMS, corresponding to the respective molecules by the rows.

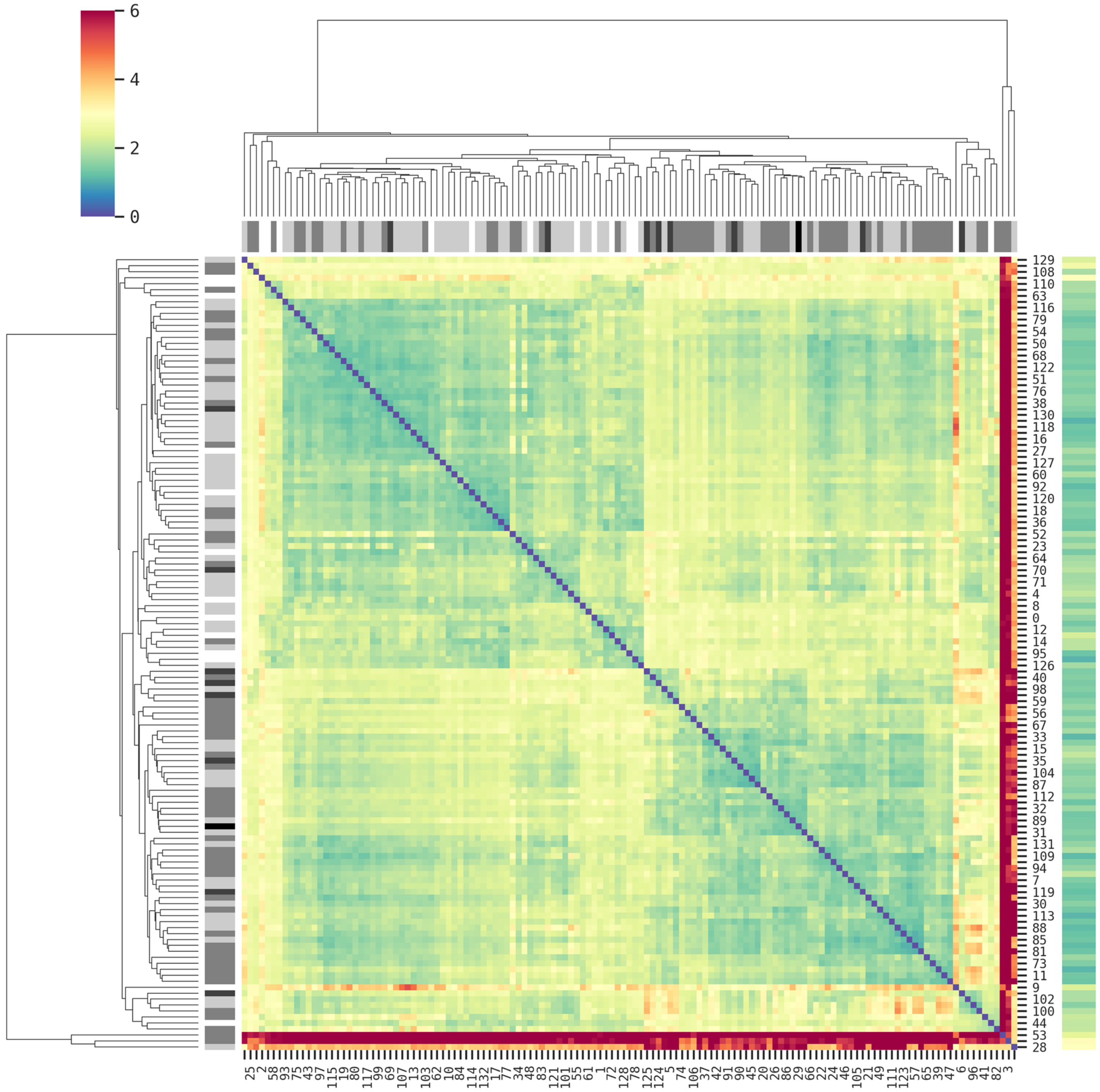

Figure S3. Heatmap and cluster analysis of EMD values between shape-context matrices of the nucleophilic Fukui functions on *close* MEMS calculated of Hirshfeld surfaces of 133 molecules utilized in the deep-learning prediction of water solubility. Evenly indexed molecules are marked on the right and oddly indexed ones are marked on the bottom. The indices can be found in Table S1. The gray bar under each dendrogram is of solubility with white, light gray, mid-gray, dark gray, and black marking LogS > -2.0, > -4.0 and <= -2.0, > -6.0 and <= -4.0, >-8.0 and <= -6.0, and <-8.0, respectively. The color bar on the far right is of the averaged EMD of each molecule among close and its four cut MEMS, corresponding to the respective molecules by the rows.

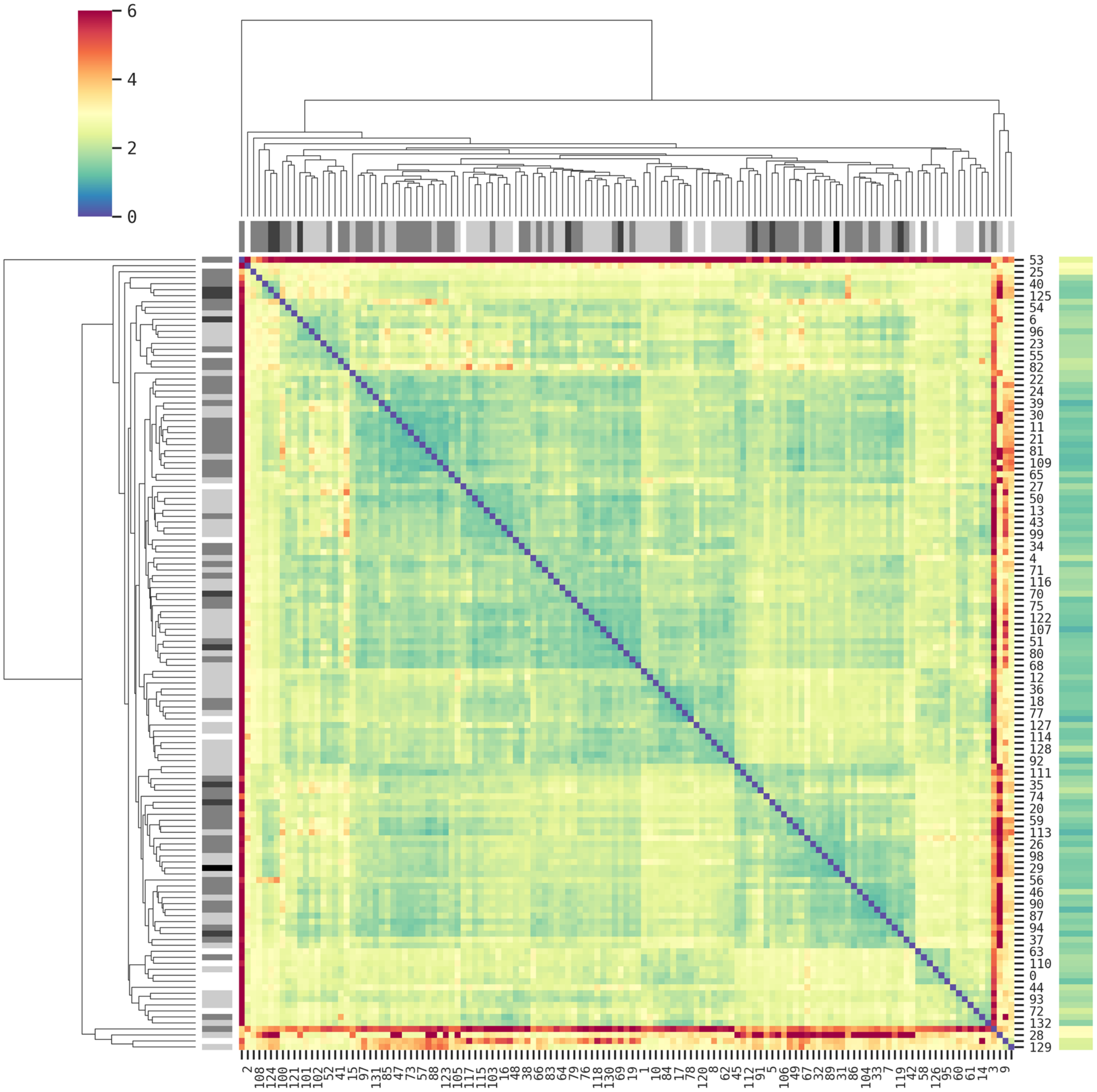

Figure S4. Heatmap and cluster analysis of EMD values between shape-context matrices of the nucleophilic Fukui functions on *cut* MEMS calculated of Hirshfeld surfaces of 133 molecules utilized in the deep-learning prediction of water solubility. Evenly indexed molecules are marked on the right and oddly indexed ones are marked on the bottom. The indices can be found in Table S1. The gray bar under each dendrogram is of solubility with white, light gray, mid-gray, dark gray, and black marking LogS > -2.0, > -4.0 and <= -2.0, > -6.0 and <= -4.0, >-8.0 and <= -6.0, and <-8.0, respectively. The color bar on the far right is of the averaged EMD of each molecule among close and its four cut MEMS, corresponding to the respective molecules by the rows.

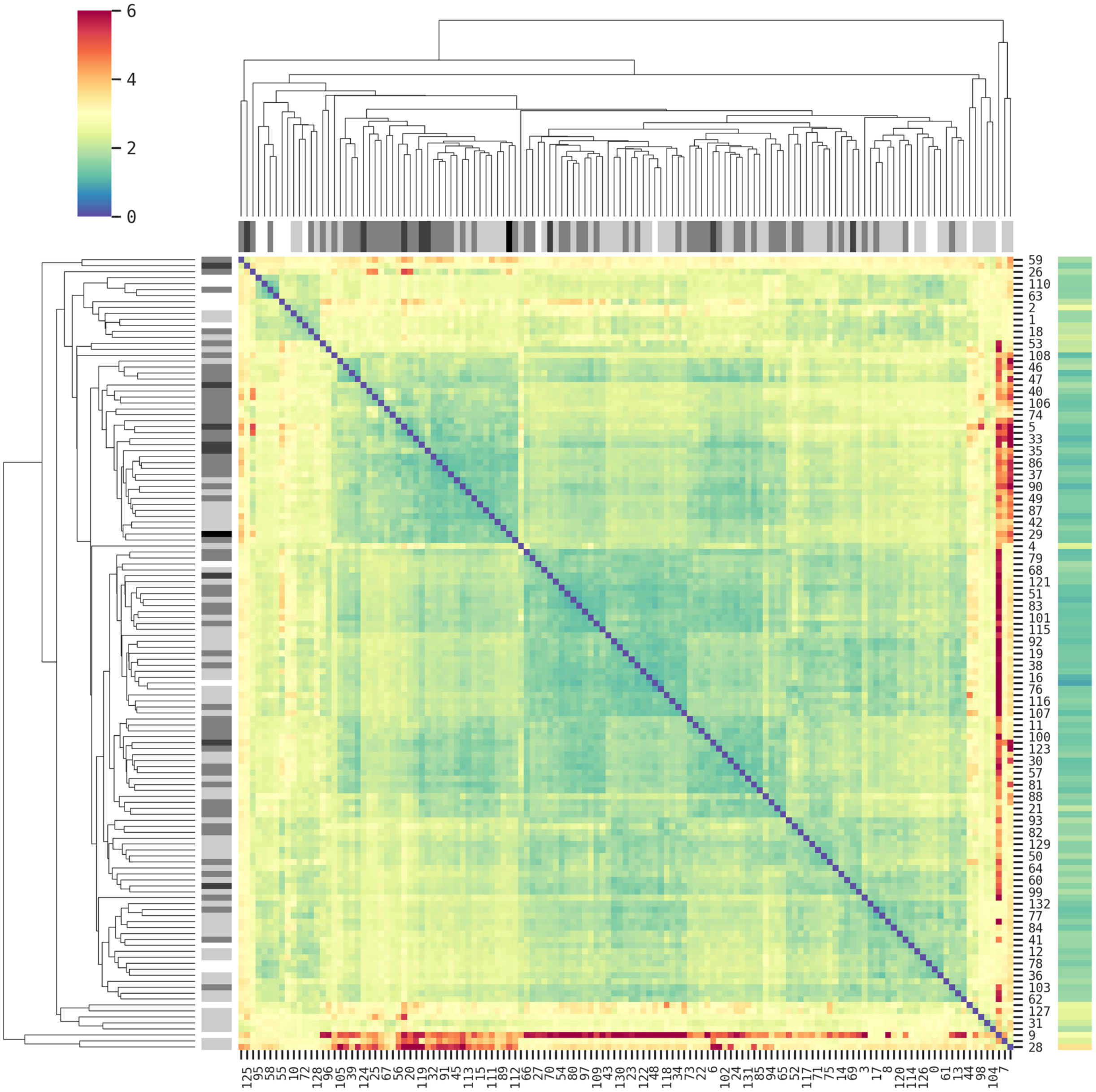

Figure S5. Heatmap and cluster analysis of EMD values between shape-context matrices of the electrophilic Fukui functions on *close* MEMS calculated of Hirshfeld surfaces of 133 molecules utilized in the deep-learning prediction of water solubility. Evenly indexed molecules are marked on the right and oddly indexed ones are marked on the bottom. The indices can be found in Table S1. The gray bar under each dendrogram is of solubility with white, light gray, mid-gray, dark gray, and black marking LogS > -2.0, > -4.0 and <= -2.0, > -6.0 and <= -4.0, >-8.0 and <= -6.0, and <-8.0, respectively. The color bar on the far right is of the averaged EMD of each molecule among close and its four cut MEMS, corresponding to the respective molecules by the rows.

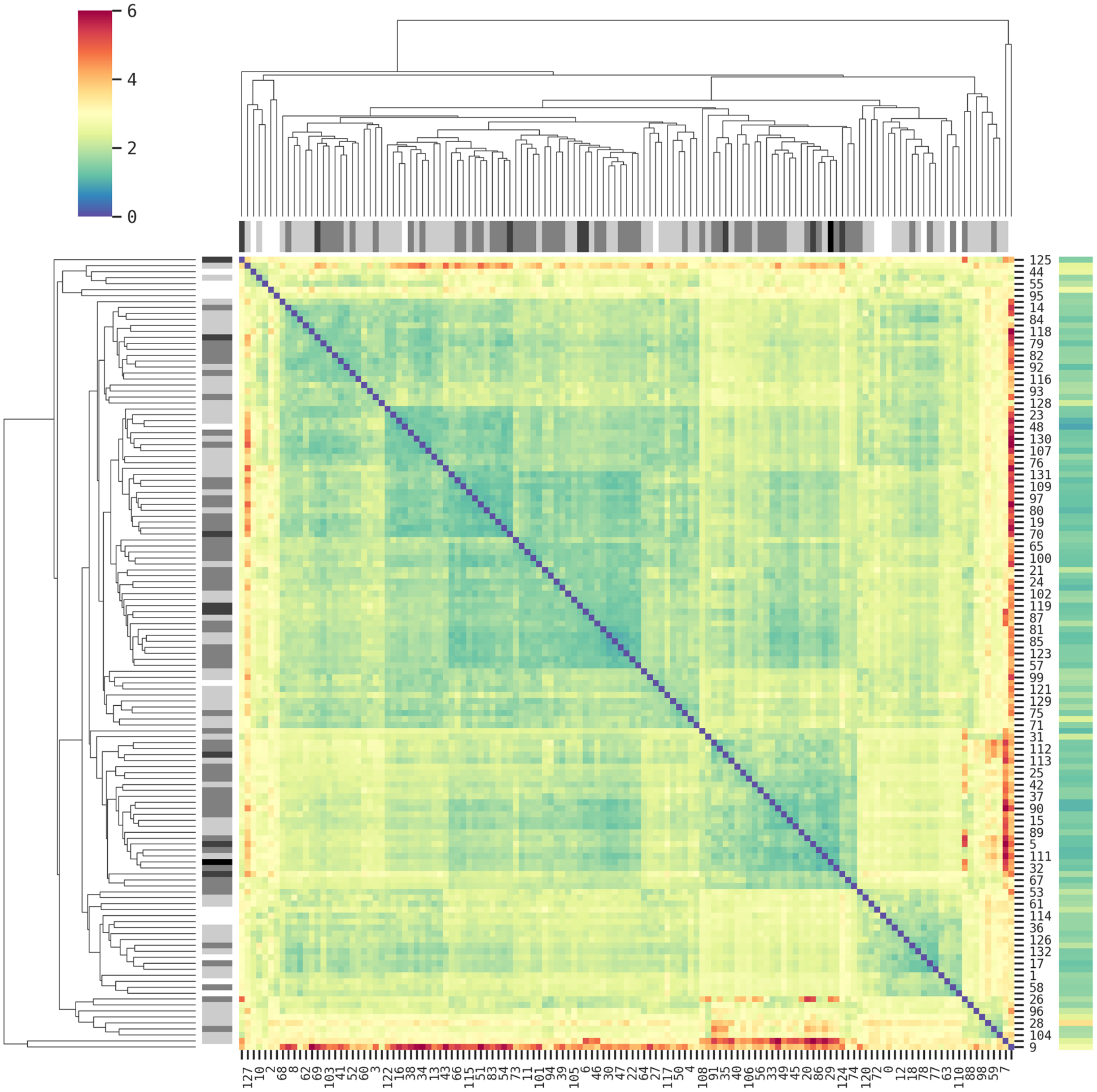

Figure S6. Heatmap and cluster analysis of EMD values between shape-context matrices of the electrophilic Fukui functions on *cut* MEMS calculated of Hirshfeld surfaces of 133 molecules utilized in the deep-learning prediction of water solubility. Evenly indexed molecules are marked on the right and oddly indexed ones are marked on the bottom. The indices can be found in Table S1. The gray bar under each dendrogram is of solubility with white, light gray, mid-gray, dark gray, and black marking LogS > -2.0, > -4.0 and <= -2.0, > -6.0 and <= -4.0, >-8.0 and <= -6.0, and <-8.0, respectively. The color bar on the far right is of the averaged EMD of each molecule among close and its four cut MEMS, corresponding to the respective molecules by the rows.

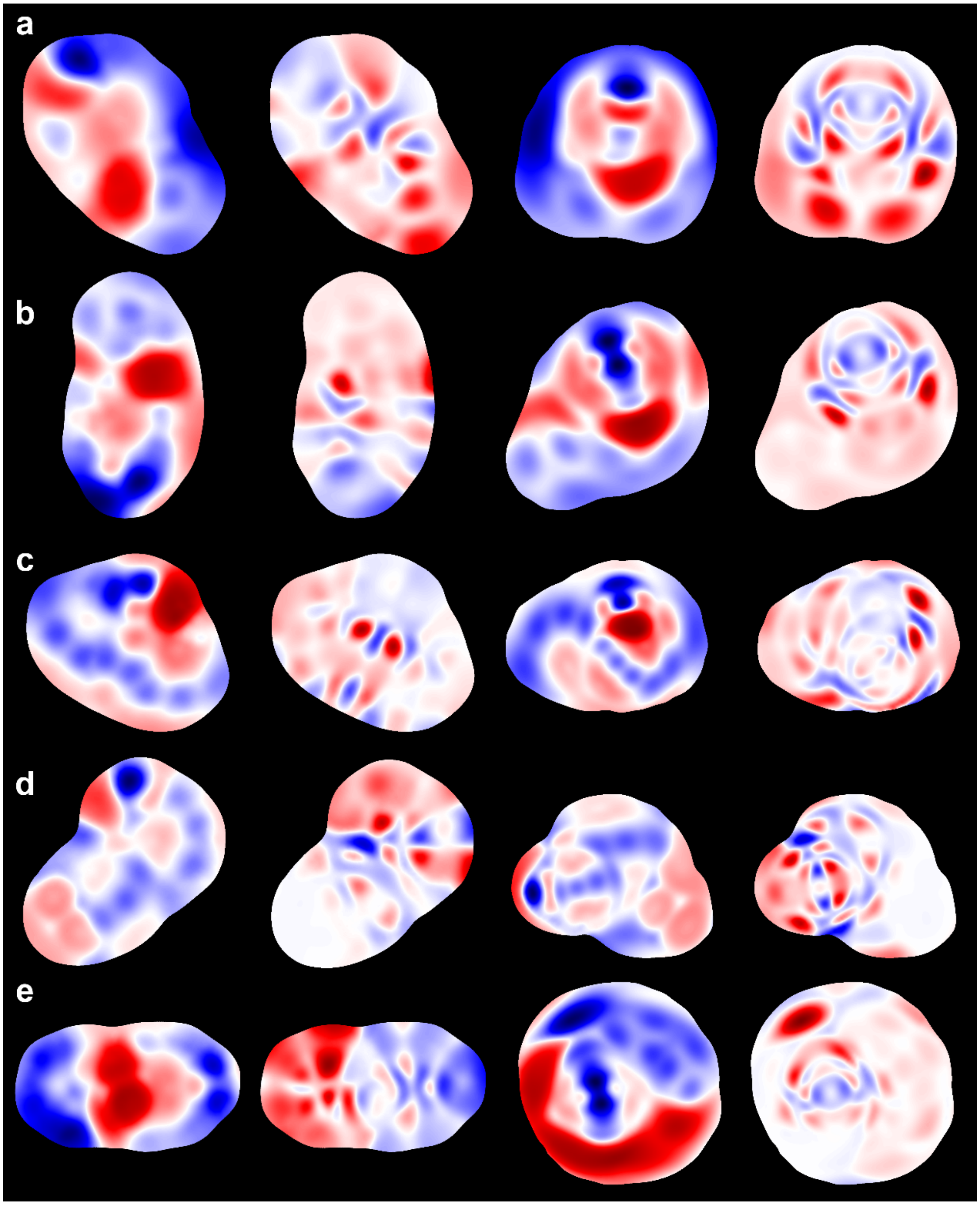

Figure S7. ESP and F^2 MEMS of four selected molecules that were generated from electronic iso-surfaces of acetaminophen (a), benzocaine (b), carbamazepine (c), flufenamic acid (d), and sulfisoxazole (e). Of each molecule, the first two are of closed (ESP and F^2) and the other two of cut MEMS. The color scheme is the same as that in Figure 2.

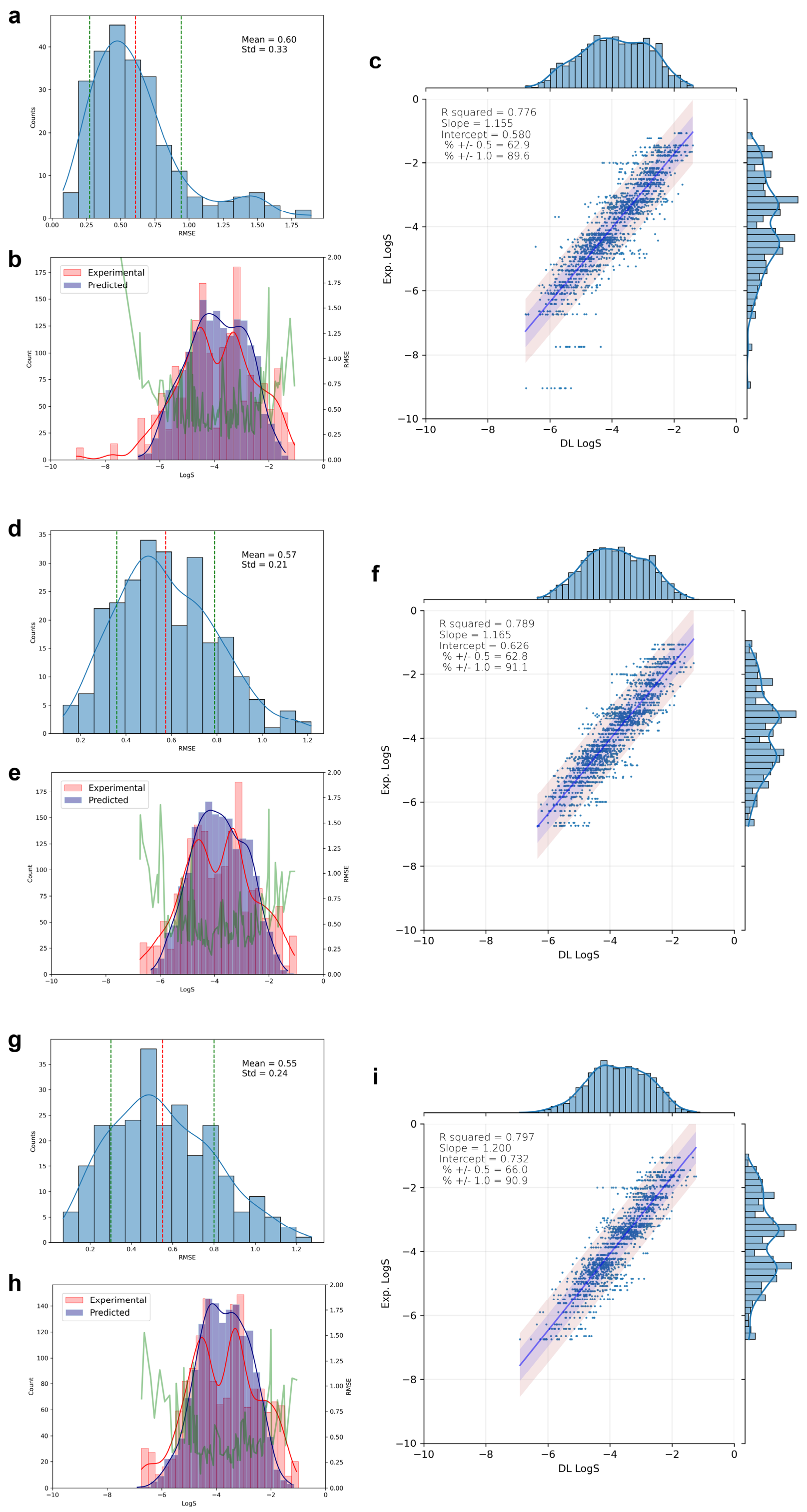

Figure S8. Deep-learning prediction results of three separated sets of deep learning with 95% of 133 molecules used in the training (a-c), 131 molecules excluding clofazimine and terfenadine (d-f), and 117 molecules excluding 16 with larger experimental errors (g-i).

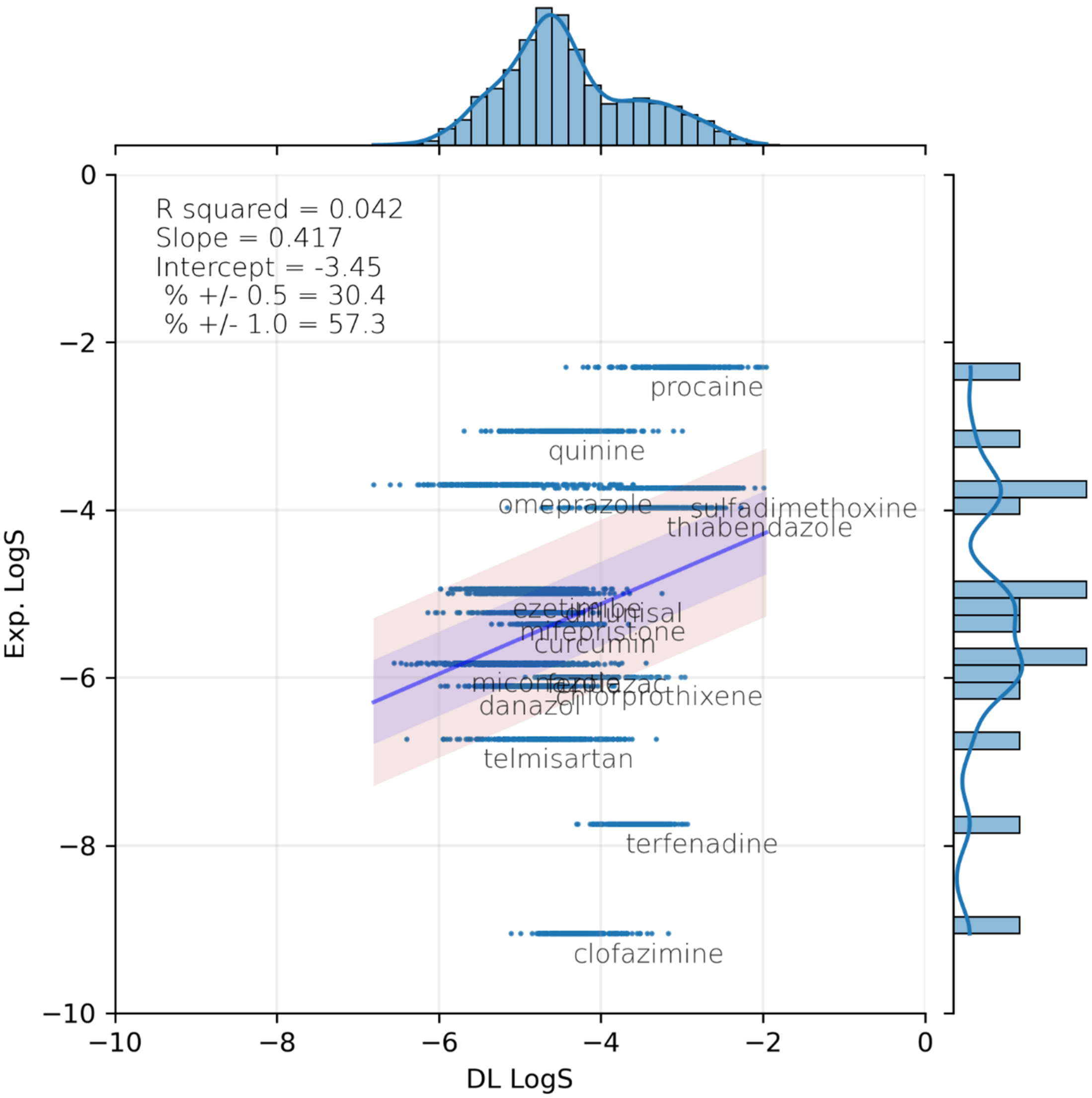

Figure S9. Deep-learning predicted vs. experimental solubility logarithm values of the 16 molecules in the second set of the second Solubility Challenge. Training was conducted with the remaining 117 molecules and repeated 64 times to keep the prediction results that had the smallest MAE. Shape-context matrices of four cut MEMS were utilized.

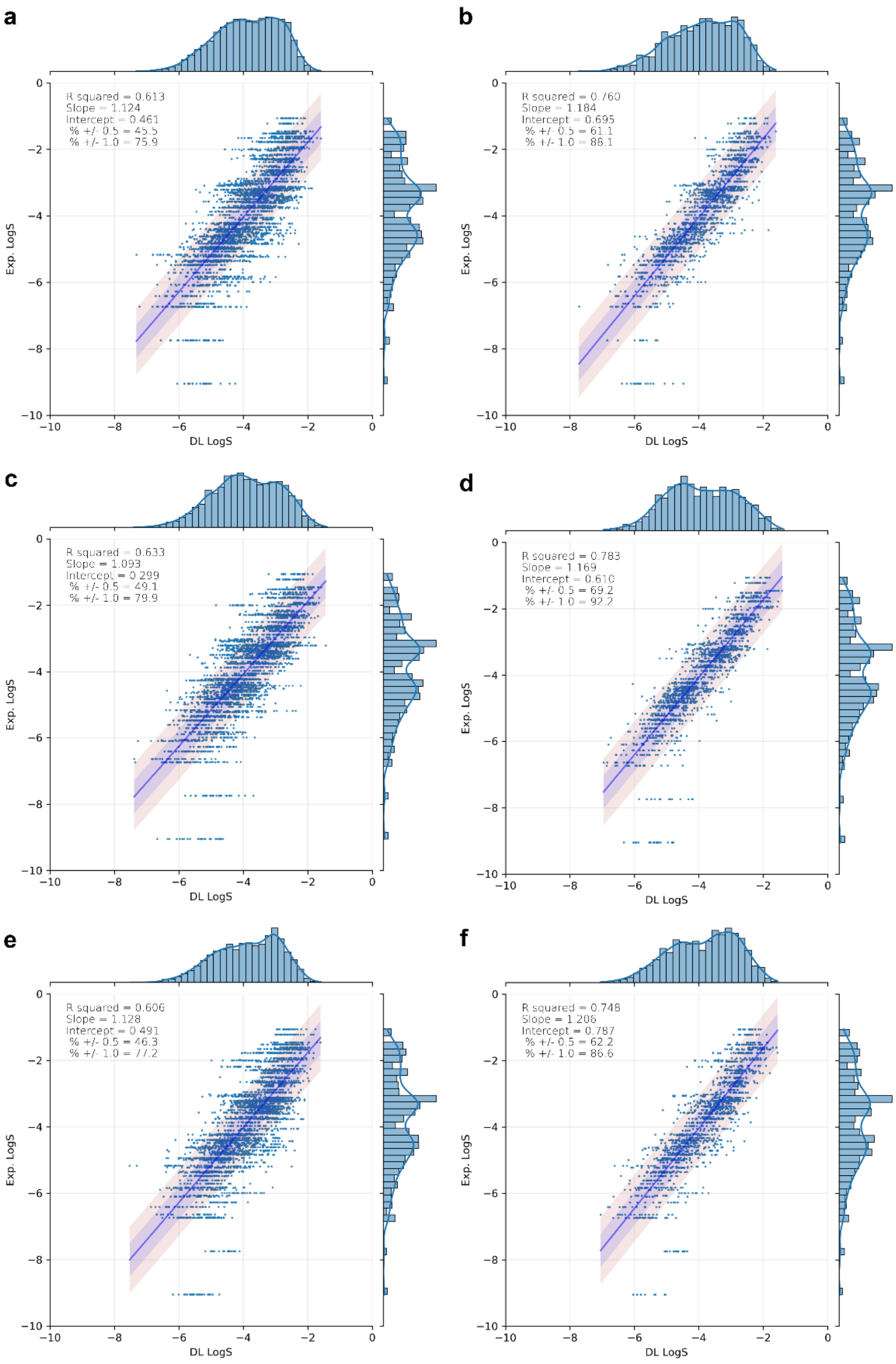

Figure S10. Deep-learning prediction versus experimental values of 133 molecules by closed MEMS with 90% and 95% data splitting (a and b), four cut MEMS of electron-density iso-surfaces with 90% and 95% splitting (c and d), close MEMS of iso-surfaces with 90% and 95% splitting (e and f).

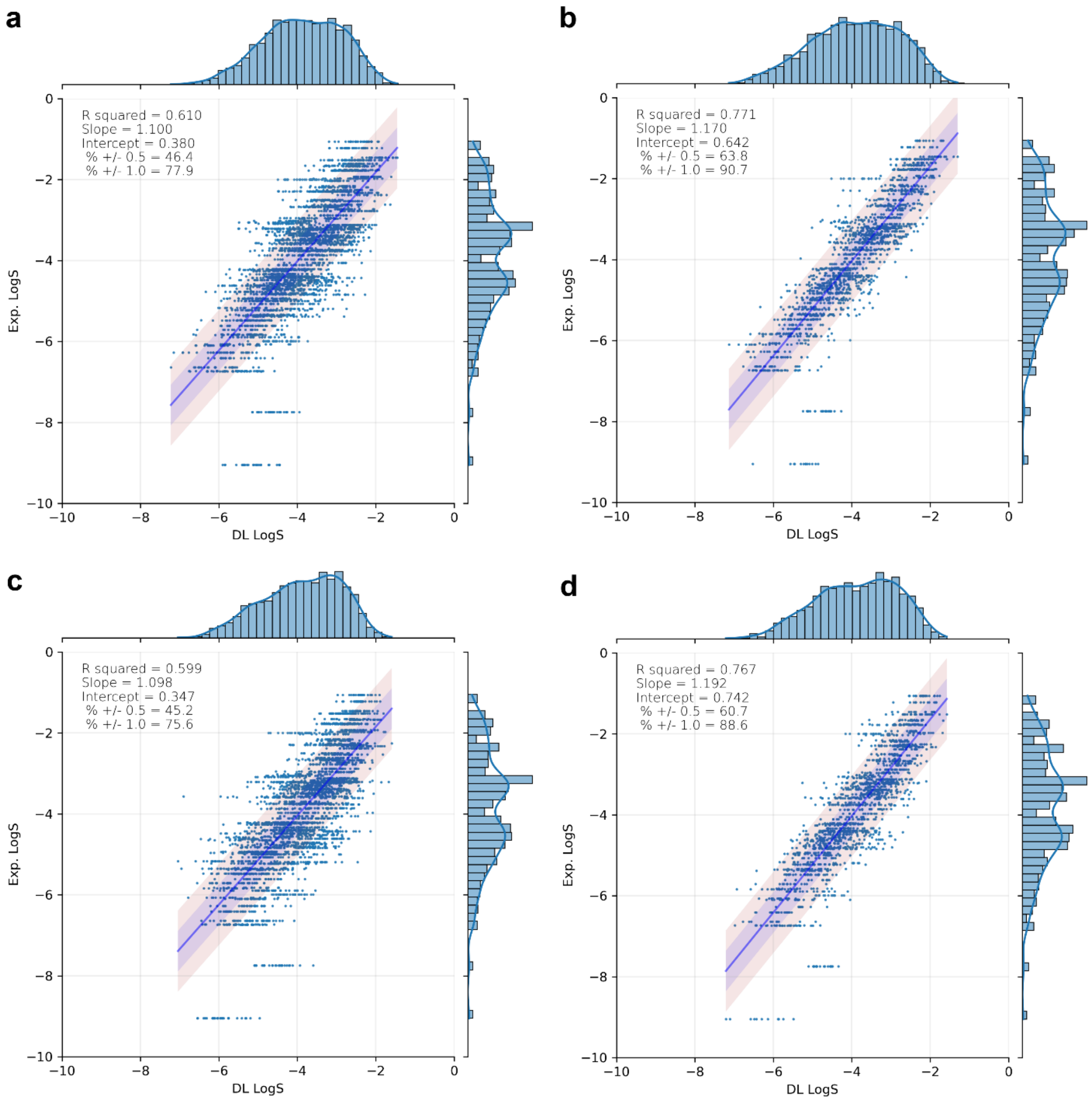

Figure S11. Deep-learning prediction versus experimental values by iso-surface MEMS of 133 fully optimized single molecules: four cut MEMS of each molecule (a), four cut trained with 95% of molecules (b), closed MEMS (c), and closed MEMS trained with 95% of molecules (f).

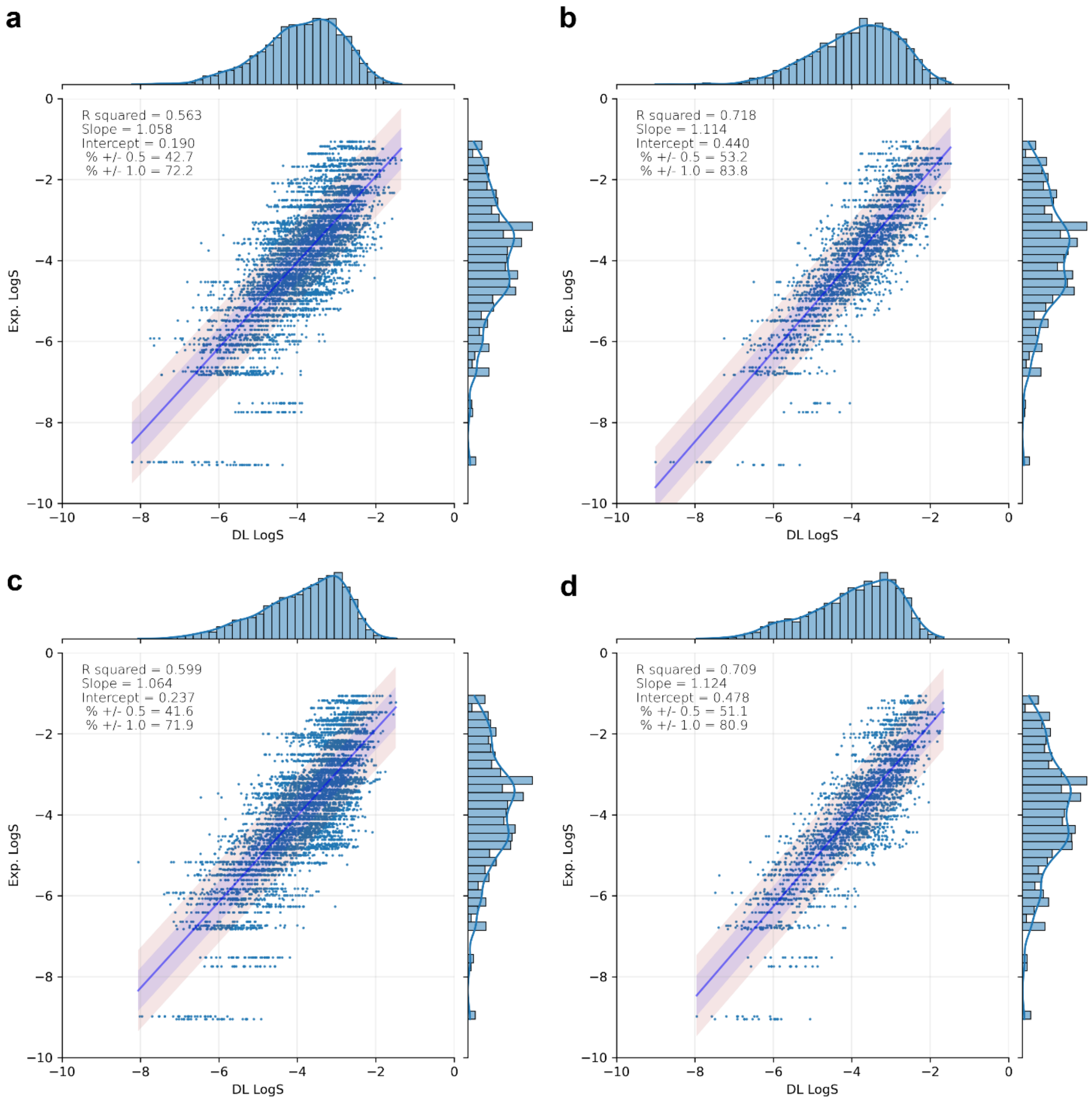

Figure S12. Deep-learning prediction versus experimental values by iso-surface MEMS of 200 fully optimized single molecules: four cut MEMS of each molecule (a), four cut trained with 95% of molecules (b), closed MEMS (c), closed MEMS trained with 95% of molecules (f).

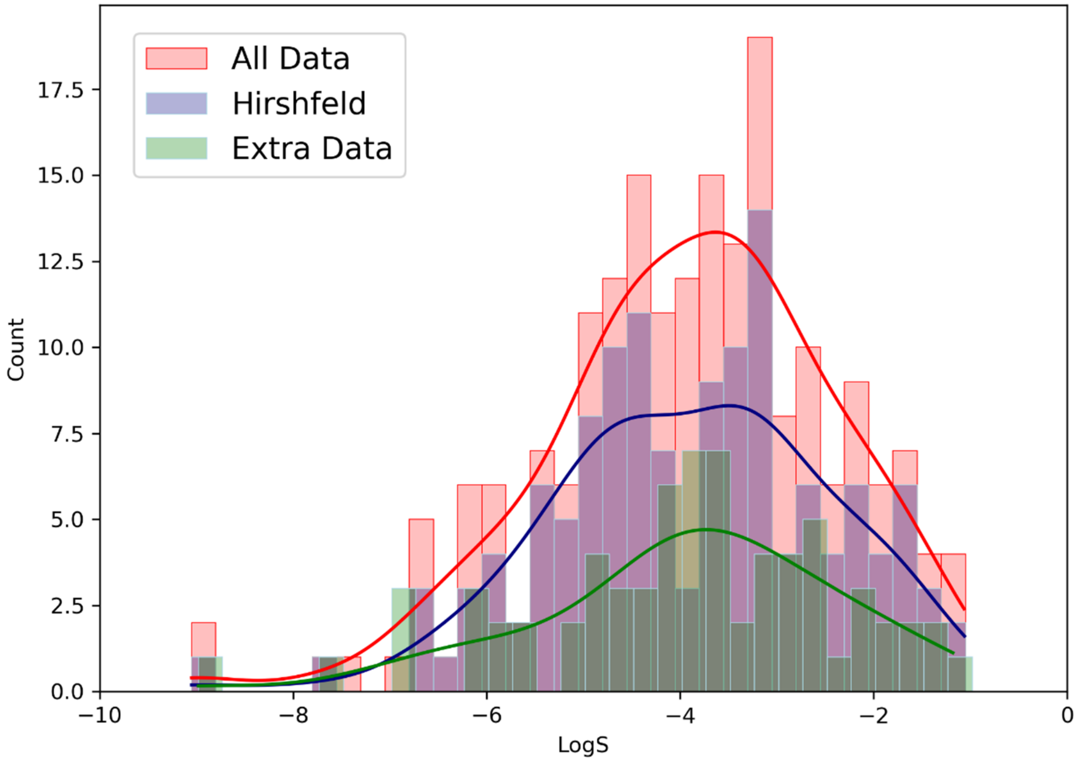

Figure S13. Data distributions of experimental values of solubility of all 200 molecules (red), 133 molecules with crystal structures (blue) and the extra 67 molecules without a proper crystal structure (green).

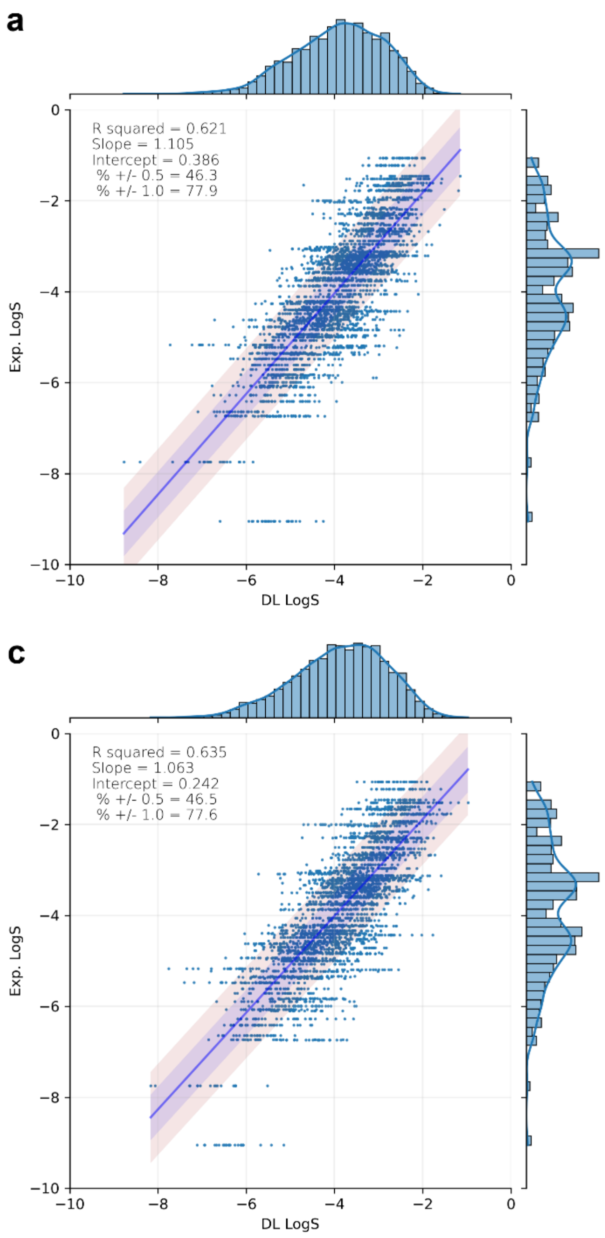

Figure S14. Shallow-learning prediction versus experimental values by Hirshfeld-surface MEMS of 133 partial optimized single molecules: four cut MEMS of each molecule (a), four cut trained with 95% of molecules (b), closed MEMS (c), closed MEMS trained with 95% of molecules (f).

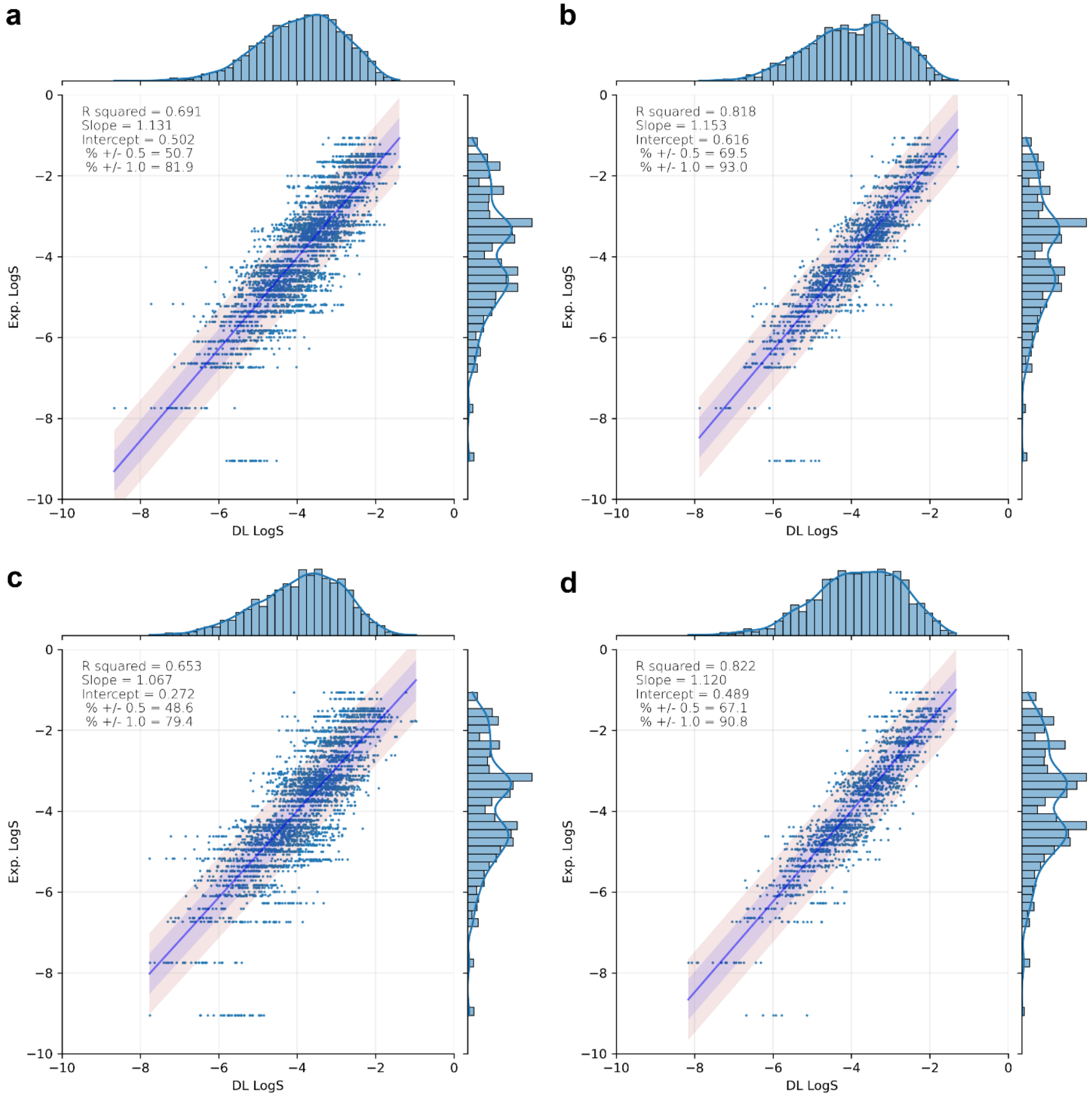

Figure S15. Shallow-learning prediction versus experimental values by iso-surface MEMS of 133 partial optimized single molecules: four cut MEMS of each molecule (a), four cut trained with 95% of molecules (b), closed MEMS (c), closed MEMS trained with 95% of molecules (f).

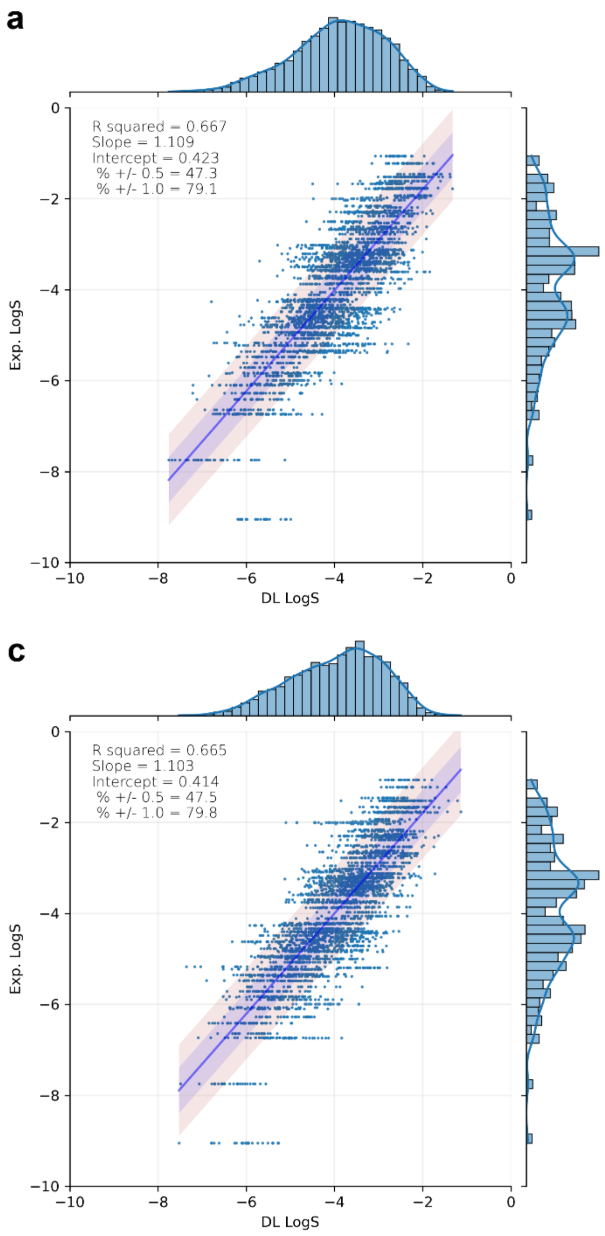

Figure S16. Shallow-learning prediction versus experimental values by iso-surface MEMS of 133 fully optimized single molecules: four cut MEMS of each molecule (a), four cut trained with 95% of molecules (b), closed MEMS (c), closed MEMS trained with 95% of molecules (f).

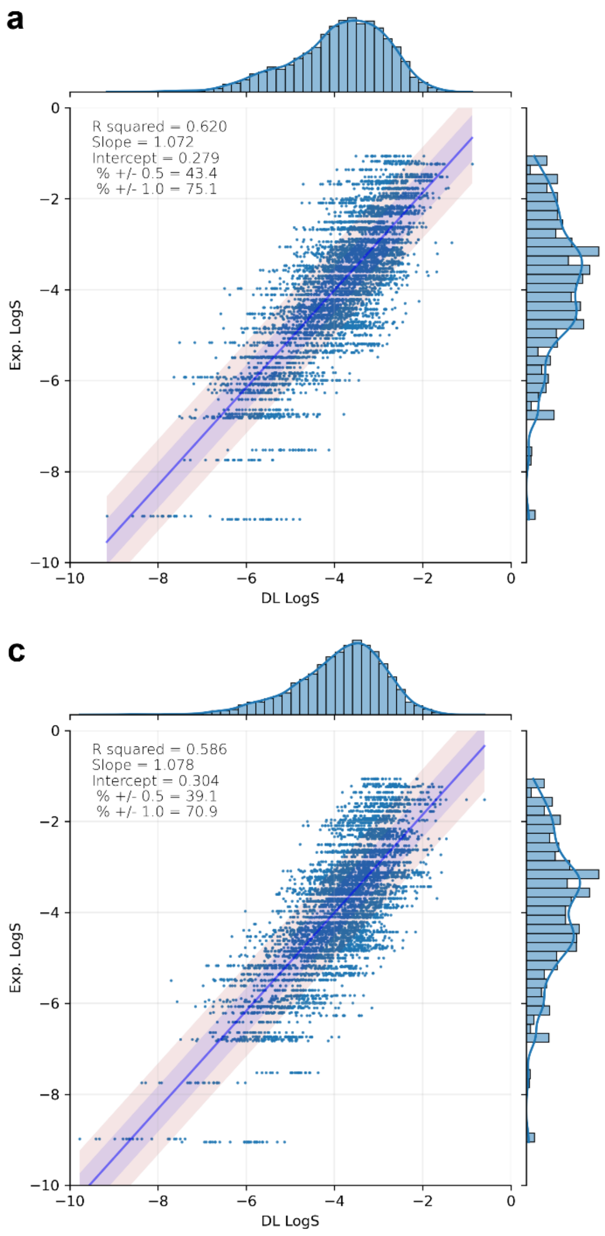

Figure S17. Shallow-learning prediction versus experimental values by iso-surface MEMS of 200 fully optimized single molecules: four cut MEMS of each molecule (a), four cut trained with 95% of molecules (b), closed MEMS (c), closed MEMS trained with 95% of molecules (f).

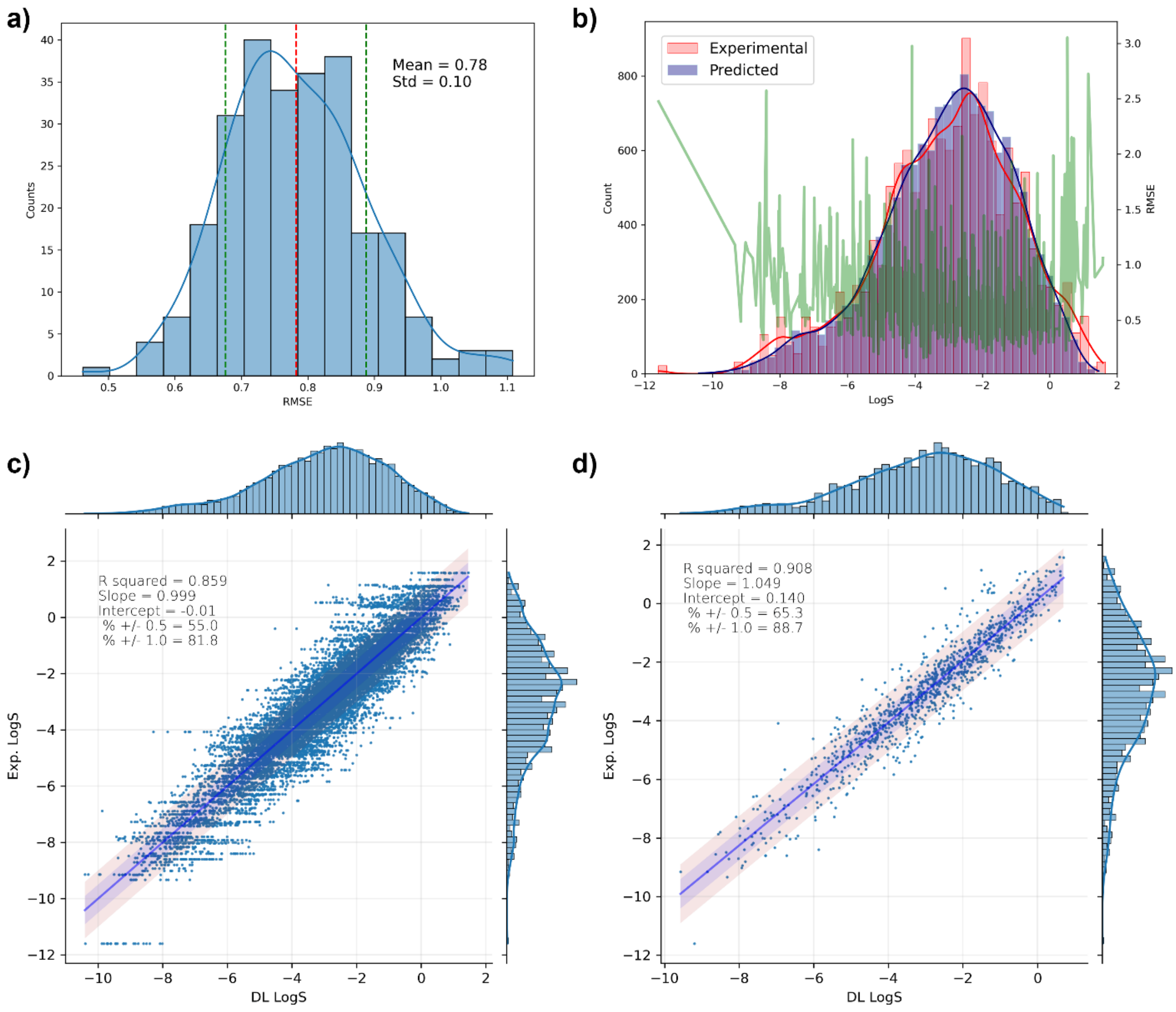

Figure S18. Deep-learning prediction results of ESOL by four cut iso-surface MEMS at 95:5 split of the dataset for training and testing, RMSE of cross validations (a), overlapped distributions of the predicted (blue) and experimental values (red) of LogS with individual RMSE values of the molecules plotted as green line (b), predicted versus experimental values of individual molecules (c), and mean predicted versus experimental values of individual molecules (d).

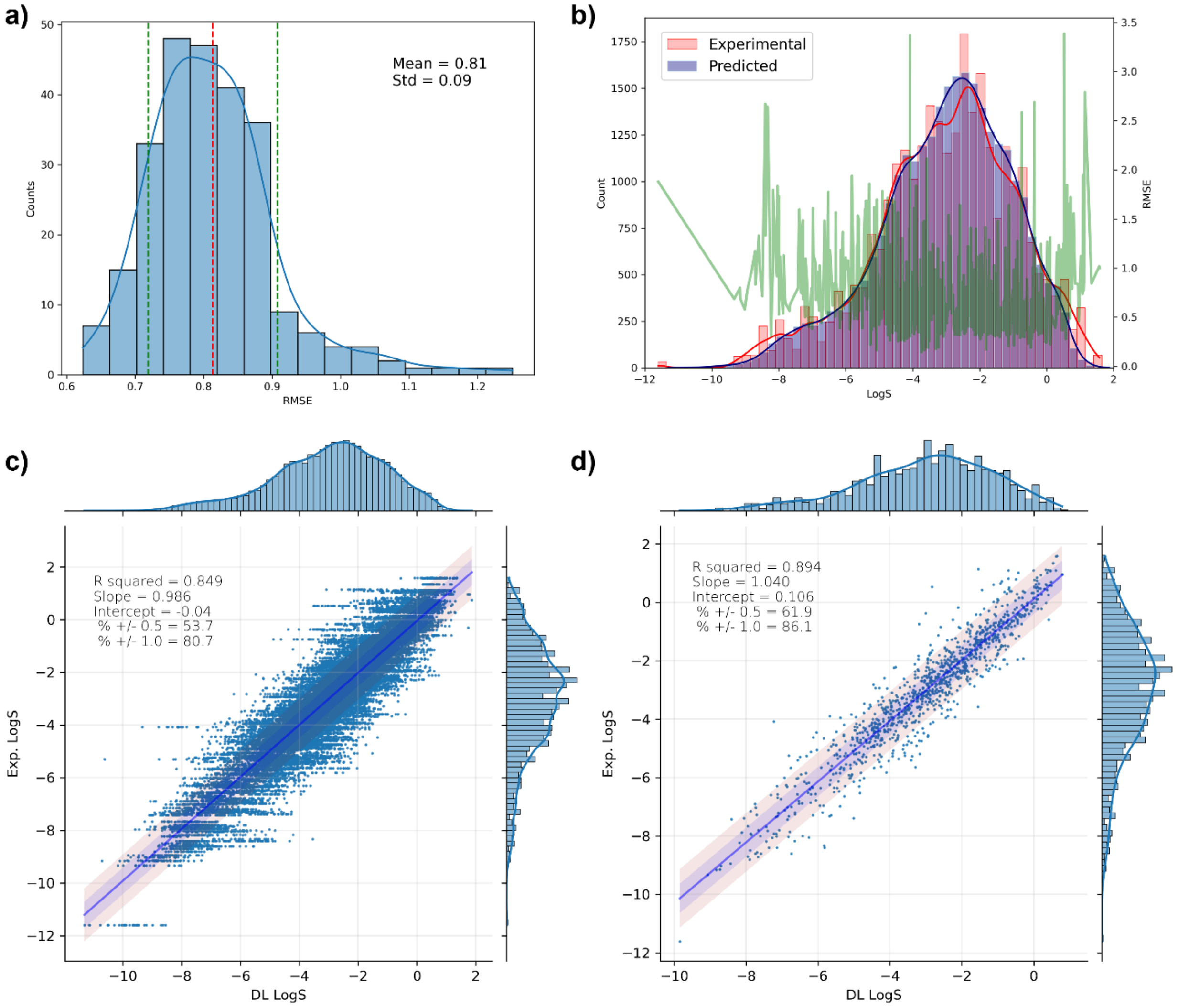

Figure S19. Deep-learning prediction results of ESOL by closed iso-surface MEMS at 90:10 split of the dataset for training and testing, RMSE of cross validations (a), overlapped distributions of the predicted (blue) and experimental values (red) of LogS with individual RMSE values of the molecules plotted as green line (b), predicted versus experimental values of individual molecules (c), and mean predicted versus experimental values of individual molecules (d).

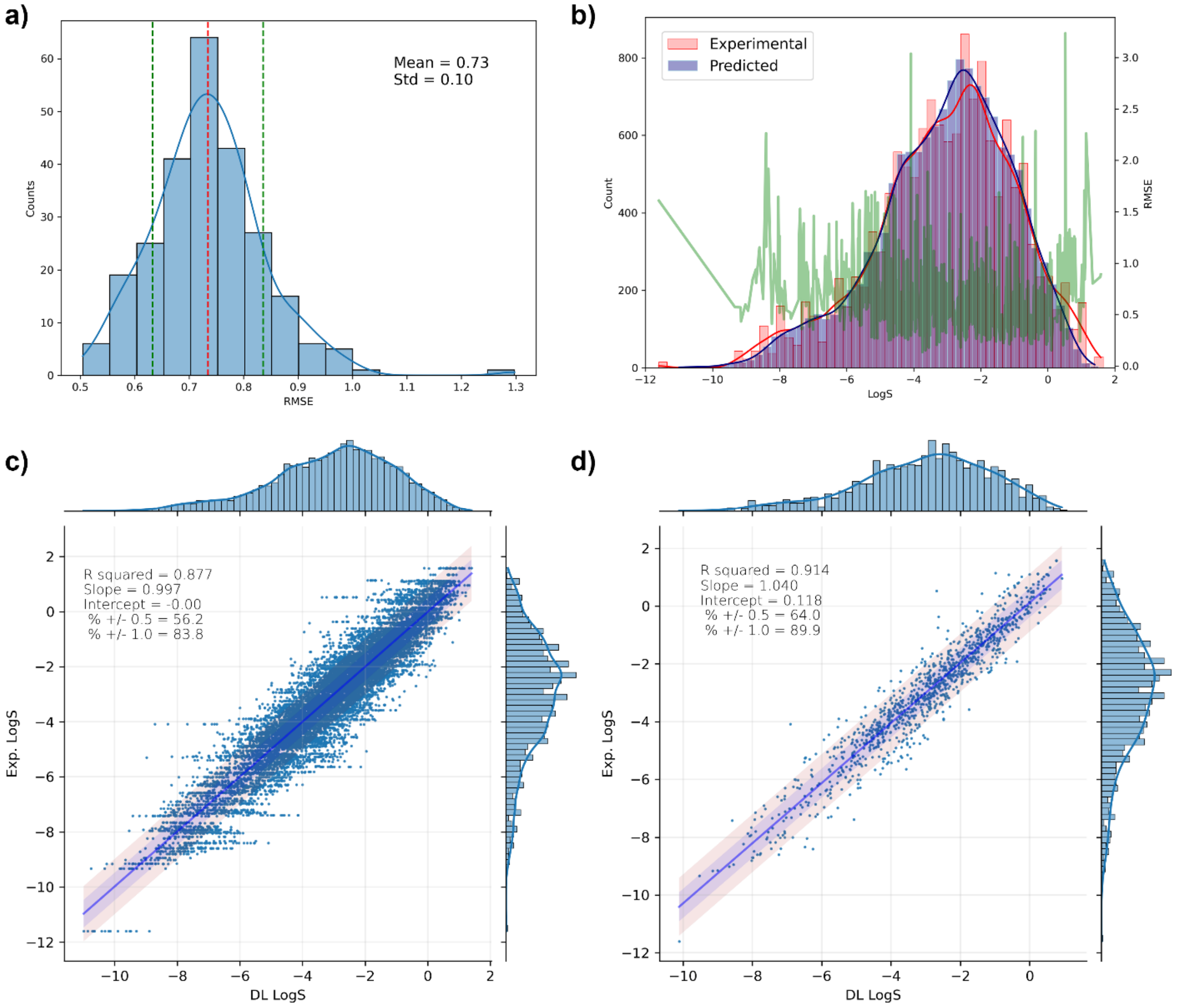

Figure S20. Deep-learning prediction results of ESOL by closed iso-surface MEMS at 95:5 split of the dataset for training and testing, RMSE of cross validations (a), overlapped distributions of the predicted (blue) and experimental values (red) of LogS with individual RMSE values of the molecules plotted as green line (b), predicted versus experimental values of individual molecules (c), and mean predicted versus experimental values of individual molecules (d).

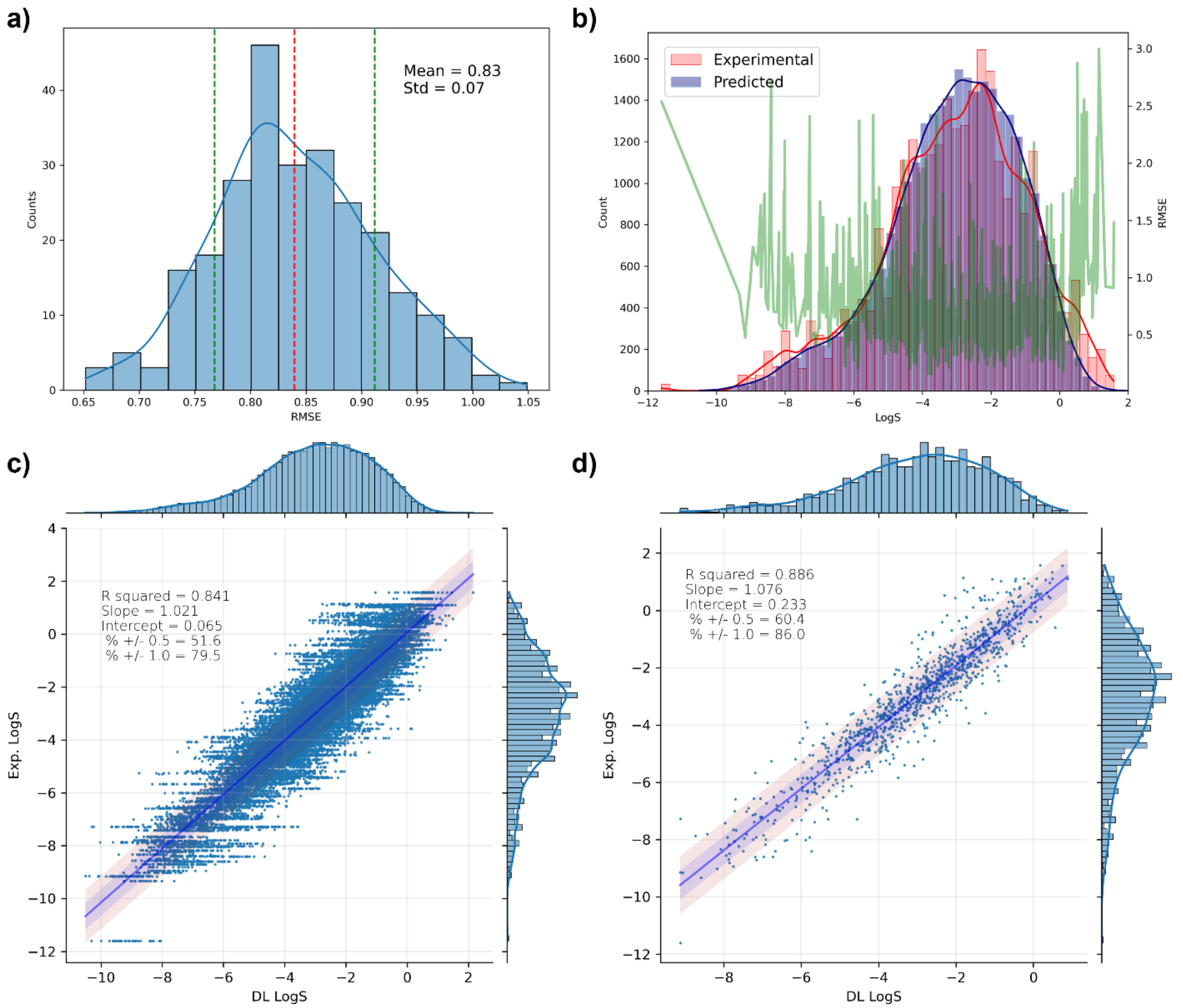

Figure S21. Shallow-learning prediction results of ESOL by four cut iso-surface MEMS at 90:10 split of the dataset for training and testing, RMSE of cross validations (a), overlapped distributions of the predicted (blue) and experimental values (red) of LogS with individual RMSE values of the molecules plotted as green line (b), predicted versus experimental values of individual molecules (c), and mean predicted versus experimental values of individual molecules (d).

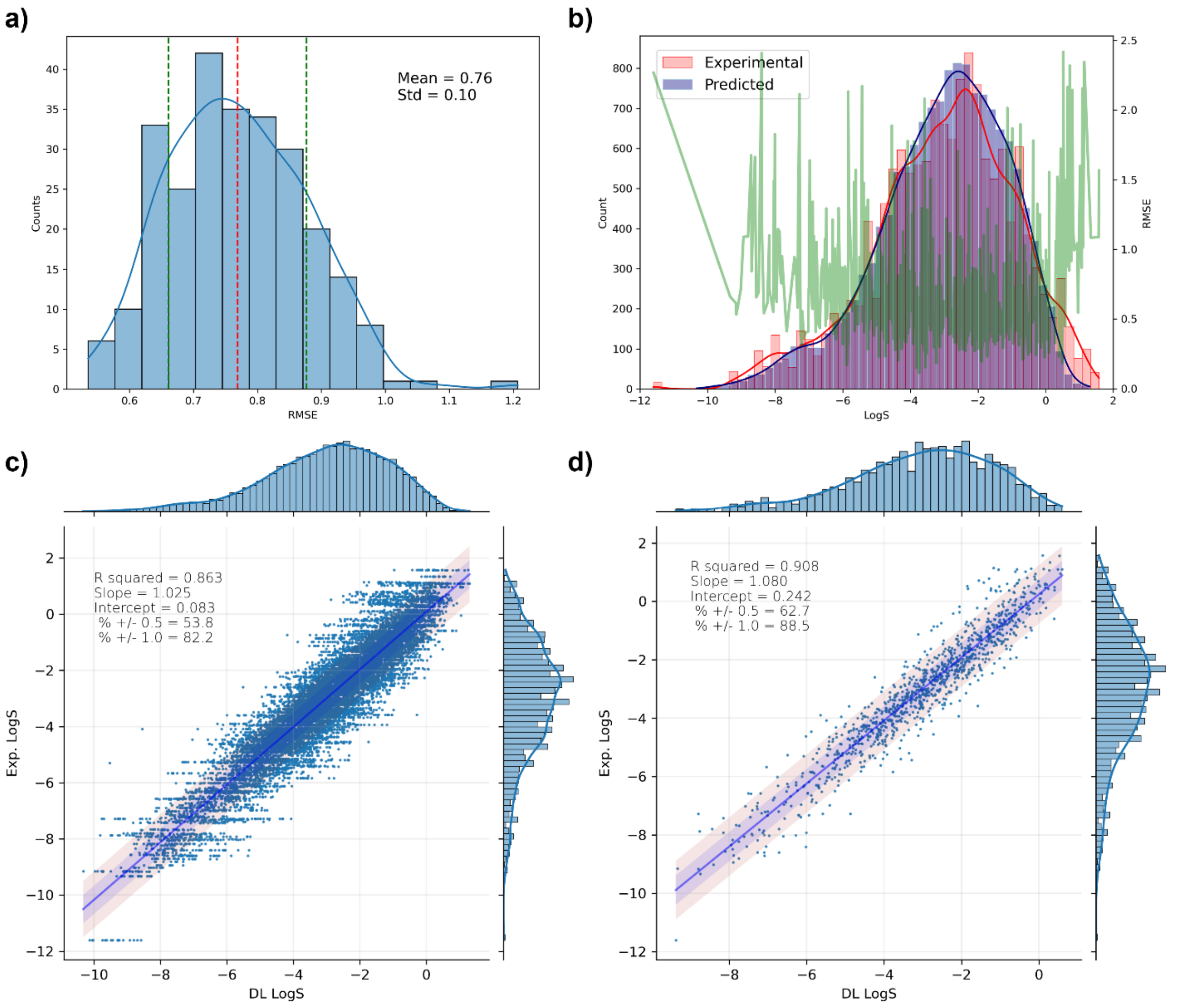

Figure S22. Shallow-learning prediction results of ESOL by four cut iso-surface MEMS at 95:5 split of the dataset for training and testing, RMSE of cross validations (a), overlapped distributions of the predicted (blue) and experimental values (red) of LogS with individual RMSE values of the molecules plotted as green line (b), predicted versus experimental values of individual molecules (c), and mean predicted versus experimental values of individual molecules (d).

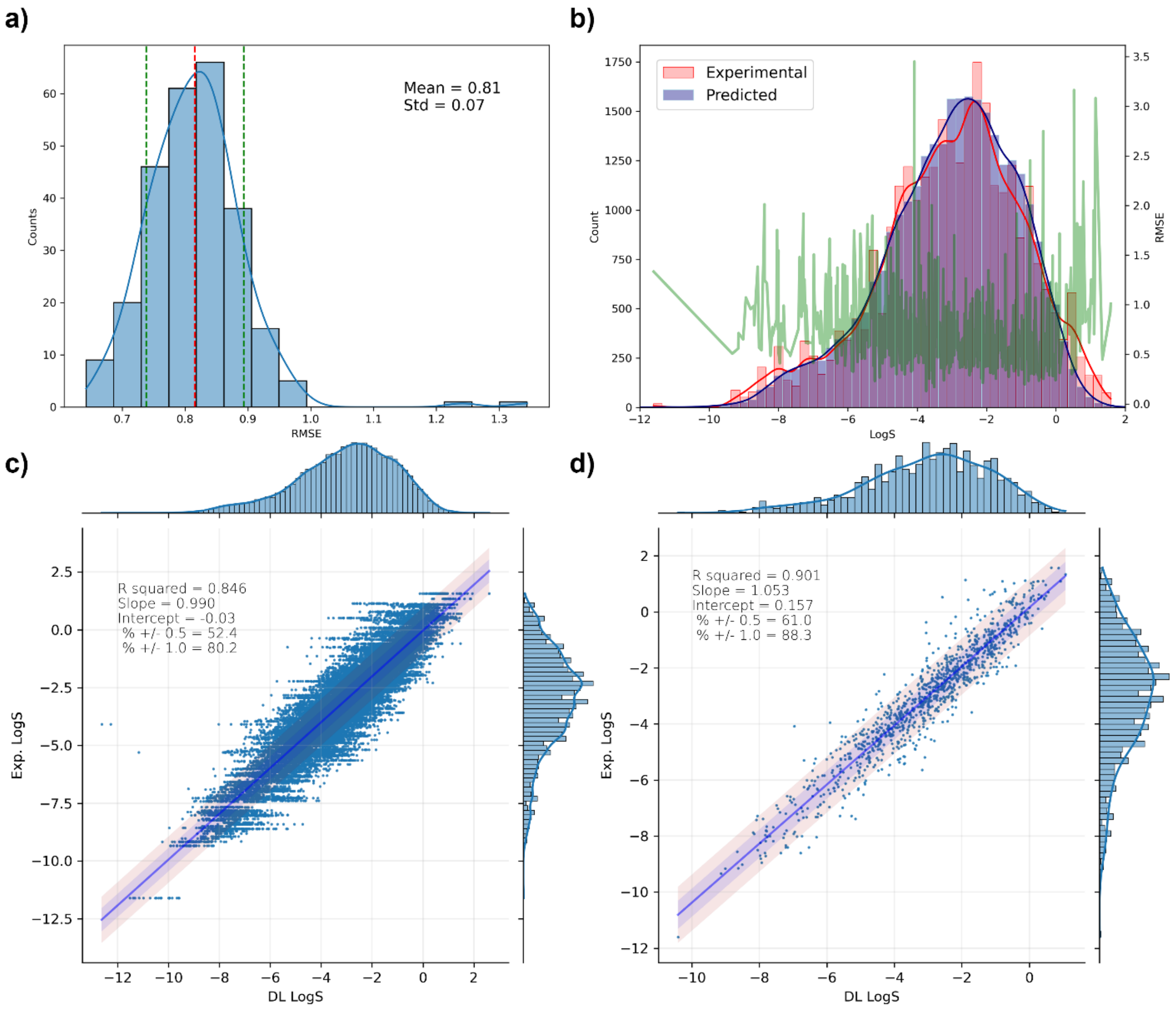

Figure S23. Shallow-learning prediction results of ESOL by one closed iso-surface MEMS at 90:10 split of the dataset for training and testing, RMSE of cross validations (a), overlapped distributions of the predicted (blue) and experimental values (red) of LogS with individual RMSE values of the molecules plotted as green line (b), predicted versus experimental values of individual molecules (c), and mean predicted versus experimental values of individual molecules (d).

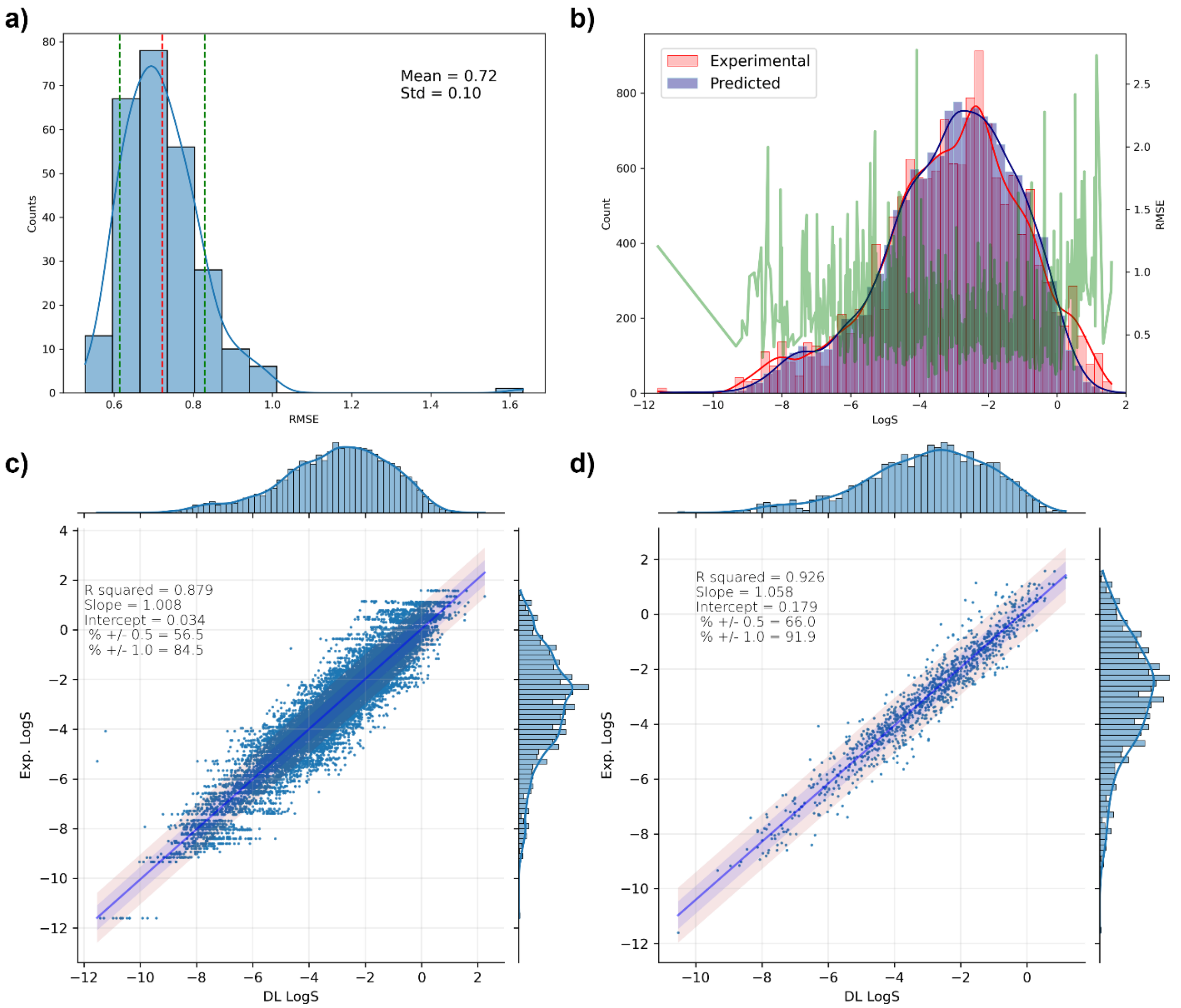

Figure S24. Shallow-learning prediction results of ESOL by one closed iso-surface MEMS at 95:5 split of the dataset for training and testing, RMSE of cross validations (a), overlapped distributions of the predicted (blue) and experimental values (red) of LogS with individual RMSE values of the molecules plotted as green line (b), predicted versus experimental values of individual molecules (c), and mean predicted versus experimental values of individual molecules (d).
